# Adaptive Molecular Evolution of *PHYE* in *Primulina*, a Karst Cave Plant

**DOI:** 10.1371/journal.pone.0127821

**Published:** 2015-06-01

**Authors:** Junjie Tao, Qingwen Qi, Ming Kang, Hongwen Huang

**Affiliations:** 1 Key Laboratory of Plant Resources Conservation and Sustainable Utilization, South China Botanical Garden, Chinese Academy of Sciences, Guangzhou, China; 2 University of Chinese Academy of Sciences, Beijing, China; Swiss Federal Institute of Technology (ETH Zurich), SWITZERLAND

## Abstract

Limestone Karst areas possess high levels of biodiversity and endemism. *Primulina* is a typical component of Karst endemic floras. The high species richness and wide distribution in various Karst microenvironments make the genus an idea model for studying speciation and local adaptation. In this study, we obtained 10 full-length sequences of the phytochrome *PHYE* from available transcriptome resources of *Primulina* and amplified partial sequences of *PHYE* from the genomic DNA of 74 *Primulina* species. Then, we used maximum-likelihood approaches to explore molecular evolution of *PHYE* in this Karst cave plant. The results showed that *PHYE* was dominated by purifying selection in both data sets, and two sites were identified as potentially under positive selection. Furthermore, the ω ratio varies greatly among different functional domains of *PHYE* and among different species lineages. These results suggest that potential positive selection in *PHYE* might have played an important role in the adaption of *Primulina* to heterogeneous light environments in Karst regions, and different species lineages might have been subjected to different selective pressures.

## Introduction

Light is not only the source of energy, but also a very important environmental factor for plant growth and survival. As sessile organisms, plants have evolved sophisticated photosensory systems to respond appropriately to their light environments. Phytochromes are specialized photosensors that perceive and interpret light signals from the environment to regulate plant growth and development throughout the whole life cycle [1]. Recent studies have revealed that phytochromes play an important role in modulating both biotic and abiotic stress [2]. In angiosperms, the phytochrome apoprotein genes have been classified into four or five gene subfamilies based on sequence similarity to the five phytochrome genes of *Arabidopsis*: *PHYA*, *PHYB*, *PHYC*, *PHYD*, and *PHYE* [3]. The *PHYB* and *PHYD* subfamilies are evolutionarily related to *PHYE*, whereas *PHYA* and *PHYC* are related to each other and formed an ancient evolutionary clade [3, 4]. As the three typical isoforms of phytochromes that are expressed widely in seed plants, the function and evolution of *PHYA*, *PHYB* and *PHYC* have been extensively studied [5, 6]. The crystal structure of *Arabidopsis* PHYB was resolved recently [7], and it provides a helpful scaffold for understanding the signaling and functional mechanism of plant phytochromes.

Previous studies have demonstrated that the evolutionary adaptation of phytochromes is associated with polymorphisms in the phytochrome genes regulating ecologically important traits [8–12]. A phylogenetic analysis suggested that positive selection in *PHYA* has played a major role in the adaptive evolution of early angiosperms [13]. Molecular evolutionary analysis of the phytochrome genes in Sorghum [14] and Brassicaceae [15, 16] have shown that the evolution of phytochromes is mainly constrained by purifying selection. More recently, population genetic studies of alpine plants have revealed positive selection in *PHYE*, suggesting its involvement in adaptation to local environments [16, 17].

*PHYE* is broadly distributed in flowering plants, expressed throughout the course of development and present in various organs [18]. At cooler temperatures, *PHYE* plays a prominent role in regulation of germination [19] and flowering [20, 21]. In addition, *PHYE* is an important contributor to germination [22, 23] and shade avoidance [24] under environments with a lower ratio of red light /far-red light (R/FR). However, the functions of *PHYE* are highly redundant with other phytochromes, especially with *PHYB* [24, 25]. In most cases, *PHYE* functions in the form of heterodimers with other phytochromes [26, 27], also mainly with *PHYB*, and fine-tuning *PHYB*-mediated physiological responses. *PHYB* is a principal mediator that responses to R and FR [5, 6], and evolves under constraints by purifying selection in *Arabidopsis* [15]. As *PHYE* has redundant function and heterodimerize with *PHYB*, functional constraints may be relaxed in *PHYE*, allowing the accumulation of amino acid replacements, thus *PHYE* may accumulate more mutations in the process of adaptive evolution. Therefore, *PHYE* should be a promising candidate gene for exploring local adaptation to different light intensity environments.

Due to the highly diverse and unique biota, limestone Karst areas in Southeast Asia have long been regarded as “natural laboratories” for ecological and evolutionary studies to understand natural selection and speciation [28]. The Karst areas in southern China have been recognized as one of the world’s centers of plant diversity [29]. The species richness in China’s Karst regions has been attributed to its large diversity of edaphic and climatic variability. *Primulina* (Gesneriaceae), a typical cave plant, is a monophyletic genus comprising more than 140 species of perennials that are widely distributed throughout the Karst regions of southern China and the adjacent countries of Southeast Asia. The genus occurs in a wide latitudinal range (18 °N-31 °N) with remarkably diverse light regime, from steep cliffs and cave entrances to twilight zones. As sessile organisms, the heterogeneous light environments exert a selection pressure on *Primulina* to survival in Karst habitats. Identifying genes targeted by natural selection can greatly improve our knowledge of the role of adaptation in species evolution. Despite recent advances in our understanding of ecophysiological adaptation to the Karst environment [30, 31], the molecular-genetic mechanisms by which *Primulina* adapts to heterogeneous light conditions have never been explored.

Although phytochromes play an important role in plant life cycle, little is known about the composition and evolution of the phytochrome gene family in *Primulina* to date. In this study, we chose *PHYE* as the target to explore whether the phytochrome involved in local adaptation of *Primulina* to the diverse light environments in Karst areas. For this purpose, we obtained 10 full-length sequences and 74 partial sequences of *PHYE* from *Primulina*, which were sampled from a wide geographic range of the genus. We used molecular evolutionary approaches to test whether positive selection or selective constraints arisen on this gene.

## Materials and Methods

### Ethics Statement

*P*. *tabacum* is listed in the Inventory of Rare and Endangered Plants of China and the Key Protected Inventory of Wild Plants of China (http://db.kib.ac.cn/eflora/View/plant/ZXBWSpecies.aspx). The leaf samples of this species were collected with permission from the greenhouse of South China Botanical Garden. All other species are not recognized as the endangered or protected species at the moment, the leaf samples used in this study were collected from open areas, and the location is not privately owned or protected in any way, so no specific permits were required for the sampling.

### Plant materials and amplification of *PHYE* genes

The plant materials used in this study were collected from fields throughout the geographic range of *Primulina* in China, as specified in S1 Table. These species are widely distributed across the phylogeny of the genus. One individual of each species was used, and a total of 74 *Primulina* species and two outgroups (*Didymocarpus hancei* and *Petrocodon dealbatus*) were included. Total genomic DNA was extracted from silica-gel dried leaves using modified CTAB methods.

Using the full-length coding sequences of *Arabidopsis thaliana PHYE* (AT4G18130) as query, we obtained 10 full-length coding sequences of *PHYE* (3369 bp) from transcriptome resources of *Primulina* species [32]. The queried sequences were checked carefully by eyes and made certain that there were no any ambiguity characters, no frame-shift mutations or premature stop codons. The stop codons were excluded in the following analysis. However, it is impractical to amplify the full-length sequences of *PHYE* (> 3369 bp) from genomic DNA for the remaining species without transcriptome resources. Thus, this study mainly focused on the conserved core signaling domains of the phytochrome, i.e. PAS and GAF [33]. This led to the two data sets analyzed in this study: full-length sequences for 10 species and partial sequences for 74 species.

The specific primers used in the amplification of the core signaling domain of the *PHYE* gene were designed according to the alignment of full length sequences using Primer Premier 5.0 (Premier Biosoft Interpairs, Palo Alto, CA), with the forward primer PHYE-F: 5’-CTGTTTTGTCATCCTCTGCTGC-3’ and the reverse primer PHYE-R: 5’-TGTGGTGAACGTAGGGTAGAATTAA-3’. Polymerase chain reaction (PCR) amplifications were performed as follows and reached 50 μl with sterile distilled water: 5 μl Takara 10×*Ex Taq* buffer (Mg^2+^ plus), 4 μl dNTP Mix (2.5 mM each),0.25 μl Takara *Ex Taq* DNA polymerase (5 U/μl), 2 μl 10 μm primers and about 20 ng DNA. Reaction conditions were as follows: 94 °C for 3 min, then 35 cycles of 94 °C for 30 s, 55°C for 30 s, and 72°C for 1 min; with a final extension of 72°C for 10 min. All of the PCR products were checked for length and yield by electrophoresis on 1% agarose gel stained with Goldview. Once purified, the PCR products were directly sequenced in both directions using the same primers as in amplification. All of the *PHYE* sequences were deposited in GenBank and accession numbers were listed in S1 Table.

### Recombination detection and phylogenetic reconstruction

The obtained sequences were aligned using Clustal W equipped in MEGA v5.2.2 [34] and proof read manually to make sure no ambiguity characters existed (S1 and S2 Files). As recombination can mislead phylogenetic and positive selection analyses [35], we used the genetic algorithm for recombination detection (GARD) [36] method implemented in the Datamonkey web-server (www.datamonkey.org) [37] to detect potential recombination breakpoints before analysis. Kishino-Hasegawa tests [38] were used to test statistical differences when potential breakpoints were detected.

**Fig 1:**
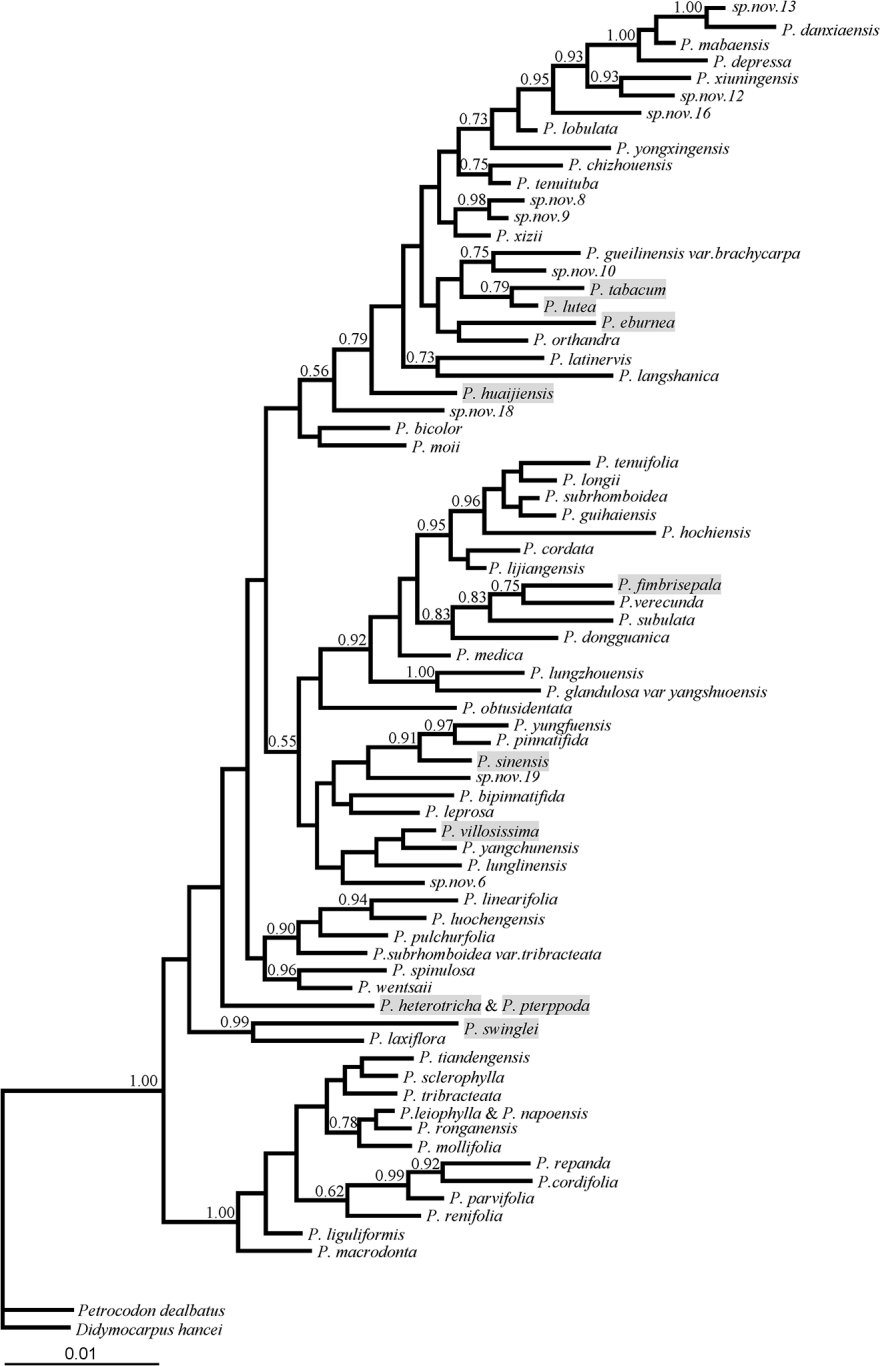
Bayesian phylogenetic tree of partial *PHYE* sequences for 74 *Primulina* species. Bayesian posterior probabilities above 0.5 were labeled at the nods. *Petrocodon dealbatus* and *Didymocarpus hancei* were used as outgroups. The species with full-length sequences are shaded.

The phylogenetic relationships were reconstructed by MrBayes v3.1.2 [39], and the best-fit nucleotide substitution models were indicated by the Akaike Information Criterion (AIC) implemented in MrModeltest v2.3 [40]. For the partial fragment of *PHYE*, the substitution models of first-, second- and third- codon position sites were F81, HKY and GTR+G, respectively. For the 10 full-length sequences, the substitution models of first-, second- and third- codon position sites were HKY+G, GTR+I and GTR+G, respectively. For the Bayesian analysis of the partial sequences, *Didymocarpus hancei* and *Petrocodon dealbatus* were set as outgroups according to our previous phylogenetic analysis of the genus[30], and the Markov Chain Monte Carlo (MCMC) search was run 8,000,000 generations and sampled every 100 generations. The first 25% of the generations were discarded as burn-in, and the remaining trees were concatenated to construct the majority rule consensus tree. For the Bayesian tree constructed for the full-length sequences, the tree was rooted at *P*. *swinglei*, as the position was supported by the larger set of taxa and located at basal position as shown in Fig 1. The MCMC was run for 100,000 generations and sampled every 100 generations, and the first 250 trees were discarded as burn-in.

### Positive selection analyses

To evaluate the influence of natural selection on *PHYE* and identify specific sites subject to positive selection, we used a variety of codon-based site-specific substitution models implemented in the CODEML program of the PAML v4.5 package [41]. The nonsynoymous/synonymous substitution ratio ω (ω = *d*_N_/*d*_S_) was estimated, where ω < 1, = 1 and > 1 indicated purifying selection, neutral evolution and positive selection, respectively. Three pairs of models with different assumed ω distributions were compared using the likelihood ratio tests (LRTs) framework to test statistical differences: one ratio model M0 versus discrete model M3, nearly neutral model M1a versus positive selection model M2a and beta model M7 versus beta & ω model M8. The Bayes empirical Bayes (BEB) analysis was used to calculate the posterior probabilities. Sites with greater posterior probability (PP > 95%) and the expected ω > 1 were inferred to be under positive selection. The analyses were run several times with different initial ω values to evaluate the convergence.

In addition, we also used other four different methods from the Datamonkey web-server to evaluate specific sites evolving under positive or purifying selection: the single likelihood ancestor counting (SLAC) method, the fixed effects likelihood (FEL) method, the random effects likelihood (REL) method [42] and the mixed effects model of evolution (MEME) [43]. For the SLAC, FEL and MEME methods, sites with *p*-values < 0.1 were accepted as candidates for selection, whereas for REL, the Bayes factor > 50 was applied.

In order to explore possible variations in selective pressure among different branches, we first tested whether the free-ratio model (M1) fits the data better than the one-ratio model (M0). M1 assumes an independent ω ratio for each branch, whereas M0 assumes all branches to have the same ω [44]. The models are compared through likelihood ratio tests (degrees of freedom = total number of branches-1). In order to detect evolutionary selection pressures acting upon individual branches, we employed the HyPhy branch site-random effects likelihood (BS-REL) method [45] as well as the PAML optimized branch-site model A method [46]. BS-REL does not require the identification of foreground branches (lineages under positive selection) and background branches (lineages lacking positive selection) *a priori*, while the branch-site models require the foreground and background branches to be defined *a priori*. Predefined biological hypotheses are unavailable, and it is difficult to define the foreground branches. Therefore, when performing branch-site model A, we treated each species branch in the phylogeny tree alternately as the foreground branch while the rest branches were considered as the background branches. LRT was constructed to compare an alternative model that allows ω to be greater than 1 in the foreground branch with a null model that restricts ω in the foreground branch equivalent to 1. The Bonferroni correction was employed to account for the problem of multiple hypotheses testing [47]. The BEB approach was also used to identify the sites that are most likely under positive selection (posterior probability > 95%).

### Sliding window analysis

To intuitively show selective variation in ω along the *PHYE* sequences, we further performed a sliding window analysis using the software SWAAP v1.0.2 [48], with window and step size of 30 bp and 3 bp, respectively. The values of ω were estimated using Nei-Gojobori [49].

## Results

### Sequence data

We obtained 10 full-length *PHYE* sequences (3369 bp) from the transcriptome resources of the *Primulina* species. The specific primer amplified partial fragment (861 bp) of *PHYE* from 74 *Primulina* species and the two outgroup species (*Didymocarpus hancei* and *Petrocodon dealbatus*). The partial length sequences correspond to positions 142-1002bp of the full-length sequences alignment, covering the complete PAS domain and part of GAF domain. The partial sequences amplified from *P*. *leiophylla* and *P*. *heterotricha* are identical with sequences from *P*. *napoensis* and *P*. *pterppoda*, respectively. We only kept unique sequences and removed those obtained from *P*. *napoensis* and *P*. *pterppoda*. Thus, a total of 10 full-length and 74 partial *PHYE* sequences were used in the following analysis.

### Phylogenetic and selection analyses

**Fig 2:**
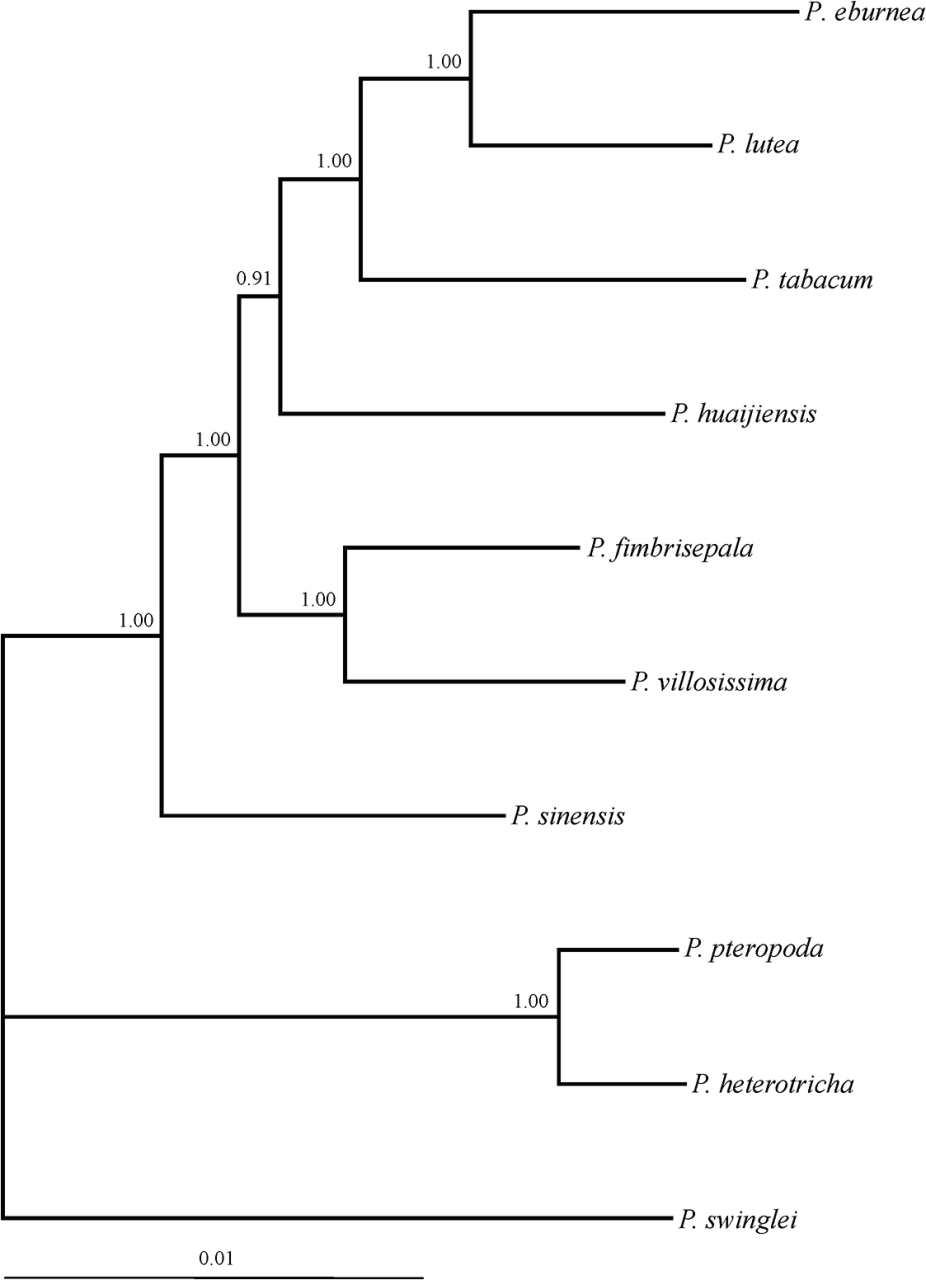
Bayesian phylogenetic tree of full-length *PHYE* sequences for 10 *Primulina* species. The tree was rooted at *P*. *swinglei*. Posterior probabilities were labeled at the nodes.

For the 74 incomplete sequences, the GARD test found no evidence of recombination. For the 10 full-length sequences, three breakpoints were identified, but they were not supported by the Kishino-Hasegawa test. Thus, the sequences can be used directly in phylogenetic reconstruction and evolutionary analysis. The Bayesian phylogenetic trees constructed by the 74 partial sequences (Fig 1) and the 10 full-length sequences (Fig 2) of *PHYE* were used in the following adaptive evolutionary analysis, respectively.

Tests for positive and purifying selection were conducted using several codon-based maximum likelihood methods. The site-specific models indicated that the 74 partial sequences were under strong purifying selection with ω = 0.128 in the one-ratio model (model M0). The discrete model M3 was significantly better than M0 (-2ΔlnL = 41.078, *p* < 0.001), indicating that the ω ratio was not homogeneous among the sites along the sequence. Positive selection model M2a was not significantly better fit to the data than the null model M1a (-2ΔlnL = 0.482, *p* = 0.786). Although one site (92) was detected under positive selection with posterior probability criterion at the 95% cutoff, the positive selection model M8 was not significantly better than the null model M7 (-2ΔlnL = 5.89, *p* = 0.053) (Table 1). Similar results were also obtained using different initial ω values. We further tested for evidence of positive selection using other four different methods implement in Datamonkey web-server. The REL analysis predicted four sites under selection (42, 92, 93 and 162), MEME found two sites (77, 92) while the SLAC and FEL each only predicted one site (92) under positive selection (Table 2). In total, these methods identified five sites (42, 77, 92, 93 and 162) under positive selection in the 74 partial *PHYE* sequences, and the site 92 was detected by almost all of the methods.

**Table 1:** Phylogenetic tests of positive selection for *PHYE* in *Primulina* using site models.

Data sets	Model	Np	lnL	Parameters	Models compared	-2ΔlnL	*p-*value	Positively selected sites (posterior probability)
**Partial sequences**	M0	147	-3166.733	ω = 0.128				None
	M3	151	-3146.194	p_0_ = 0.353, ω_0_ = 0	M0-M3	41.078^**^	2.6×10^–8^	42T(0.989); 73H(0.676); 77C(0.585)
				p_1_ = 0.613, ω_1_ = 0.142				92L(1.000); 93M(0.985); 134T(0.512)
				p_2_ = 0.033, ω_2_ = 1.196				162A(0.907)
	M1a	148	-3146.796	p_0_ = 0.946, ω_0_ = 0.079				Not allowed
				p_1_ = 0.054, ω_1_ = 1				
	M2a	150	-3146.555	p_0_ = 0.949, ω_0_ = 0.081; p_1_ = 0.048	M1a-M2a	0.482	0.786	42T(0.605); 77C(0.531)
				ω_1_ = 1; p_2_ = 0.003, ω_2_ = 3.052				92L(0.832); 93M (0.569)
	M7	148	-3149.285	p = 0.259, q = 1.627				Not allowed
	M8	150	-3146.34	p_0_ = 0.967, p = 1.138	M7-M8	5.89	0.052	42T(0.828); 73H(0.541); 77C(0.613); 92L(0.968)
				q = 11.09, p_1_ = 0.033, ω = 1.3				93M(0.792); 134T(0.548);162A(0.662)
**Full-length sequences**	M0	19	-6347.891	ω = 0.193				None
	M3	23	-6341.618	p_0_ = 0.62, ω_0_ = 0; p_1_ = 0.378	M0-M3	12.546*	0.014	28A(0.687)
				ω_1_ = 0.494; p_2_ = 0.002, ω_2_ = 5.409				
	M1a	20	-6341.718	p_0_ = 0.879, ω_0_ = 0.087				Not allowed
				p_1_ = 0.121, ω_1_ = 1				
	M2a	22	-6341.669	p_0_ = 0.888, ω_0_ = 0.093; p_1_ = 0.111	M1a-M2a	0.098	0.952	27A(0.515); 28A(0.647); 597G(0.502)
				ω_1_ = 1; p_2_ = 0.001, ω_2_ = 5.523				
	M7	20	-6341.805	p = 0.177, q = 0.719				Not allowed
	M8	22	-6341.635	p_0_ = 0.998, p = 0.245	M7-M8	0.34	0.844	19D(0.618); 27A(0.655); 28A(0.813)
				q = 1.039, p_1_ = 0.002, ω = 5.39				472M(0.566); 516K(0.614); 597G(0.639)
								611E(0.608); 694E(0.596); 1025P(P.604)
								1079T(0.576); 1083T(0.56)

For the partial sequences, the amino acids refer to *P*. *tenuifolia;* For the full-length sequences, the amino acids refer to *P*. *eburnea*

Np: number of estimated parameters; lnL: log likelihood score

^*^ Significant at *p* < 0.05

^**^ Significant at *p* < 0.01.

**Table 2:** Positive selection analysis using SLAC, FEL, REL and MEME methods.

Data sets	Positive selection sites				
	Mean *d*_N_/*d*_S_	SLAC^a^(*p*-value)	FEL^b^(*p*-value)	REL^c^ (Bayes Factor)	MEME^d^ (*p*-value)
**74 partial sequences**	0.162	92 (0.029)	92 (0.021)	42 (543.984); 92 (6825.22)	77 (0.012); 92 (0.025)
				93 (331.067); 162 (85.6759)	
**10 full-length sequences**	0.225	—	28 (0.084)	19 (71.725); 28 (127.283)	—
				198 (59.846); 516 (64.578)	
				597 (76.37); 611 (63.669)	
				694 (62.788); 1025 (62.025)	
				1079 (60.866); 1083 (58.532)	

For the partial sequences, the amino acids refer to *P*. *tenuifolia*; For the full-length sequences, the amino acids refer to *P*. *eburnea*

Sites identified by more than one method are shaded.

^a^ SLAC: single likelihood ancestor counting; codons with *p-*values < 0.1

^b^ FEL: fixed-effect likelihood; codons with *p-*values < 0.1

^c^ REL: random effect likelihood; codons with Bayes Factor > 50

^d^ MEME: mixed effects model of evolution; codons with *p*-values < 0.1.

In the analysis of the 10 full-length *PHYE* sequences, no evidence of positive selection was detected in the full length sequences using site models in CODEML. Similar to the partial sequences, the full-length sequences were under strong purifying selection with ω = 0.193 in the one-ratio model M0, and the ω was also not homogeneous among sites along the full-length sequences, as the discrete model M3 was significantly better than M0 (-2ΔlnL = 12.546, *p* = 0.014). The positive models M2a and M8 were not significantly better than the null models M1a and M7, respectively, and no sites were found to be under positive selection using the BEB approach with a posterior probability at the 95% level (Table 1). Given the methods from the Datamonkey web-server, 1 and 10 selected sites were detected under positive selection by FEL and REL, respectively, and only one site (28) was detected by both of the methods whereas no site was detected by SLAC and MEME (Table 2).

In order to explore possible variation in selective pressure among different lineages and to identify sites subject to episodic selection, which represents selection along one or a few lineages, we first compared the free-ratio model (M1) with one-ratio model (M0). The results showed that M1 fits the data better significantly than M0 for both of the data sets (Table 3), indicating that different *Primulina* species experienced variable levels of selective pressure. Then we used the branch site-random effects likelihood (BS-REL) method and branch-site model to identify specific lineages on which a subset of sites have evolved under positive selection. For the two data sets, BS-REL found no branches to be under selection at *p* < 0.05 level. The branch-site model also found no branches under positive selection in the full-length sequences, while for the partial sequences, the *P*. *eburnea* branch was found to be under selective pressure (-2ΔlnL = 9.656, *p* value = 0.002), and one site (77) was identified under episodic positive selection (Table 4). However, the LRT test was not statistically significant after performing Bonferroni correction (Bonferroni critical value = 0.0007) (Table 4).The episodic positive selection at this site was also detected by the MEME method (Table 2). MEME method allows the distribution of ω to vary from site to site and from branch to branch at a site, and is capable of identifying both pervasive and episodic positive selection [43].

**Table 3:** Likelihood ratio test (LRT) stasticts for models of variable selective pressure among branches.

Data sets	Model	−2ΔlnL	Degree of freedom	p-value
**Partial sequences**	M0 versus M1	255.462	144	3.1×10^–8^
**Full-length sequences**	M0 versus M1	27.037	16	0.041

M0 and M1 are one ratio model and free-ratio model, respectively.

lnL: log likelihood scores; -2ΔlnL: likelihood ratio test (LRT) to detect positive selection.

**Table 4:** PAML branch-sie model A analysis to identify branches under episodic positive selection.

Data set	Foreground branch	Parameters under null model	lnL (null)	Parameters under alternative model	lnL(alternative)	-2ΔlnL	*p*-value	Degree of freedom	Positively selected sites	Bonferroni critical value
**Partial sequences**	*P*. *eburnea*	p_0_ = 0.854; p_1_ = 0.042; p_2a_ = 0.098; p_2b_ = 0.005; ω_0_ = 0.079; ω_1_ = 1; ω_2_ = 1	-3146.039	p_0_ = 0.948; p_1_ = 0.047; p_2a_ = 0.004; p_2b_ = 0.0002; ω_0_ = 0.08; ω_1_ = 1; ω_2_ = 299.076	-3141.211	9.656	0.002	1	77C (0.967) 85S (0.626)	0.0007

lnL: log likelihood scores; -2ΔlnL: likelihood ratio test (LRT) to detect positive selection.

### Sliding window test results

**Fig 3:**
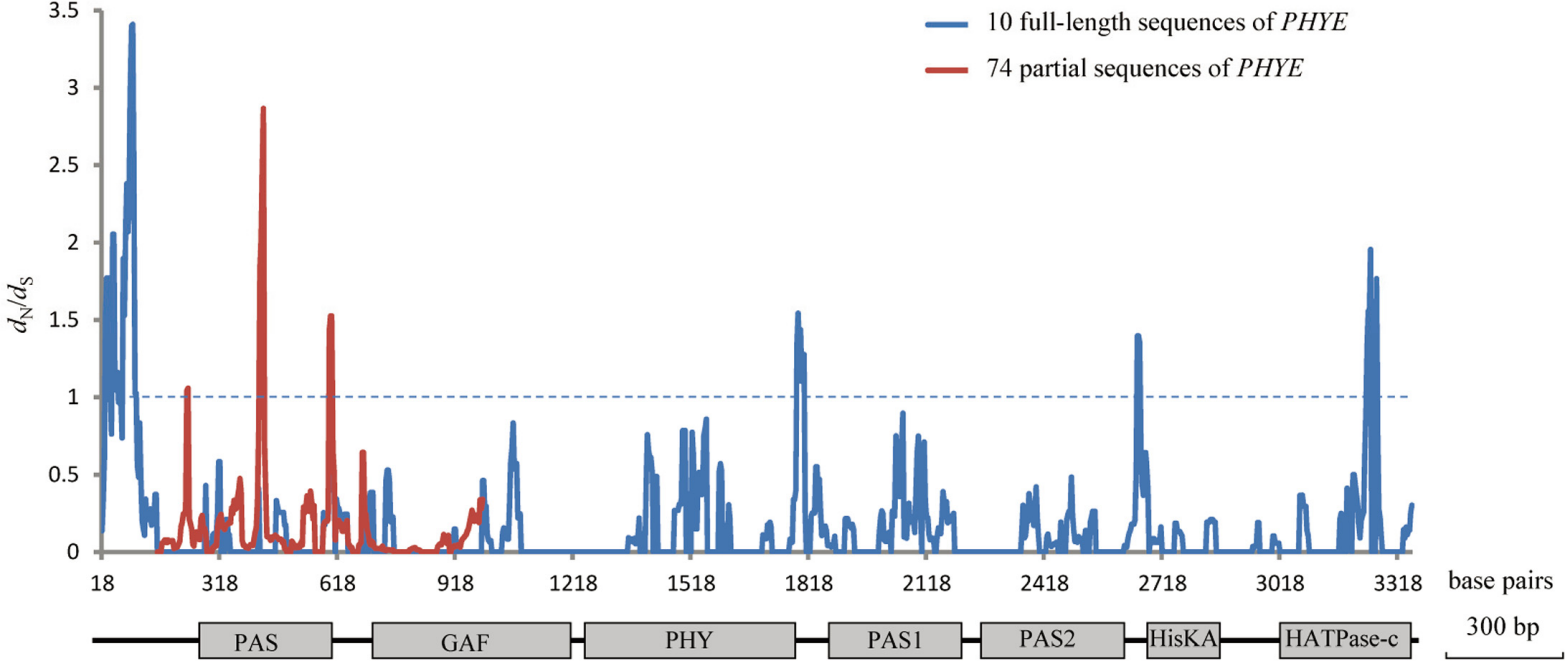
Sliding window analysis show variation of ω value along the *PHYE* gene from 74 partial sequences and 10 full-length sequences. The estimates were based on the Nei-Gojobori method. The window size was set at 30 bp and step size was set at 3 bp. Beneath the plot is a schematic of *PHYE*, which illustrates the distribution of the characteristic functional domains.

The results of the sliding window analyses of ω (*d*_N_/*d*_S_) variation across the partial and full-length sequences of *PHYE* are presented in Fig 3. Similar to the results of the site-specific and Datamonkey methods, the ω values were not homogeneous across the gene, and there were dramatic variations along the domain structure. For the 74 partial sequences, which contained the complete PAS domain and a portion of the GAF domain, the analysis revealed 3 peaks that exceed 1, mainly located in the PAS and nearby areas. The 10 full-length sequences had 4 peaks greater than 1 and were mainly located in the 5’-end region, the PHY-PAS1 domain and the HATPase-c domain. It is worth noting that the ω values of the 5’-end and the 3’ region were more variable than other domain regions. However, considering the results of selection analyses, the sliding window results may not be statistically significant. Nevertheless, it reflects the discrete ω (*d*_N_/*d*_S_) variation among the different gene functional domains.

## Discussion

In this study, we used several maximum-likelihood methods to explore the adaptive evolution of phytochrome *PHYE* in a diverse cave plant *Primulina* with two different data sets: 74 partial sequences and 10 full-length sequences. The results showed that the evolution of *PHYE* was mainly constrained by purifying selection within *Primulina*, and the selective pressure is variable among different species lineages. Two sites (77, 92) subject to episodic diversifying selection were identified from partial sequences.

For the 74 partial sequences, site 92 was identified as candidate site under positive selection with posterior probabilities > 0.95, whereas the additional required LRT with the null model for model M8 was not significant (-2ΔlnL = 5.89, *p* = 0.053) (Table 1). However, the four methods (SLAC, FEL, REL and MEME) implemented in Datamonkey web-server detected five candidate sites under positive selection and the site 92 was detected by all of the methods (Table 2). The results of the 10 full-length sequences were similar to those of the partial sequences, but the site-models failed to detect significant candidate sites under positive selection (Table 1). The methods implemented in Datamonkey detected 10 candidate sites under positive selection in full-length sequences and only site 28 was detected by two methods of FEL and REL (Table 2). The site 77 was identified by branch-site model A in partial length sequences but without statistical significance after Bonferroni correction, this site was also identified by MEME method. Combined the results from PAML and Datamonkey, one sites (92) in 74 partial sequences and one site (28) in 10 full-length sequences were detected under selection by at least two methods. Furthermore, MEME identified two sites (77 and 92) from partial sequences as potentially under episodic positive selection. These results together suggest positive selection at these sites may have played a major role during the adaptive evolution of *PHYE* to local Karst environments. The results of different selection test between the two data sets could be explained by the fact that the full-length data set (10 sequences) contained fewer sequences than the partial data set (74 sequences), and therefore decreased the power of detecting positive selection at individual sites. Alternatively, the adaptive importance of *PHYE* may be heterogeneous across species lineages, as evidenced by our branch-site test (Table 4), which demonstrated that the *P*. *eburnea* branch might be under selective pressure while others subject to selective constraints. *P*. *eburnea* is the most widespread species of the genus, and is distributed in diverse light environment conditions, while most other species are narrow endemics and single-site endemics are very common. Although no evidence of widespread positive selection acting on *PHYE* in *Primulina* was identified in the present study, the signature of potential positive selection at a few sites may suggest the involvement of *PHYE* in local adaptation. Nevertheless, sequencing longer fragments of *PHYE* from more species should allow for a much more robust test of natural selection on this gene.

The crystal structure of *Arabidopsis thaliana* PHYB was resolved recently [7]. The phytochrome molecule consists of a conserved N-terminal photosensory core domain and a C-terminal regulatory domain. The photosensory domain can be further divided into three consecutive subdomains, PAS, GAF and PHY, which are conserved among phytochromes [33]. The core signaling domain of phytochrome comprises the PAS and GAF domains. The PAS domain is involved in the incorporation of chromophore and the GAF domain is connected to the bilin chromophore and is much conserved. The PHY domain is necessary for fine-tuning phytochrome activity. The regulatory domain harbors consecutive PAS1, PAS2 and histidine kinase-related domains. Previous works on the adaptive evolution of phytochromes have mainly focused on the GAF and PHY domains. For example, two positively selected sites were identified in the GAF domains of phytochromes in Gymnosperm [50]. Moreover, positive selections detected in the GAF and PHY domains of *PHYA* were assumed to be involved in the adaptive evolution of early angiosperms [13]. The positively selected sites in the phytochromes of angiosperms demonstrated that positive selection might have driven functional divergence after gene duplication [46]. In a study on *Cardamine nipponica*, the nonsynonymous substitution detected in the PHY domain provided evidence of the involvement of *PHYE* in local adaption in alpine plants [16]. In this study, the molecular evolutionary analysis of 74 partial sequences mainly focused on the PAS and GAF domains, and found that the higher ω values fluctuate across the PAS domain and nearby regions, while the GAF domain was highly conserved. Although accumulated ω was detected in the PAS domain across partial sequences, this did not rule out other regions of *PHYE* as targets of positive selection in *Primulina*. The tendency toward ω accumulation across the 10 entire sequences was not the same as that for the partial sequences in the PAS domain (Fig 3); ω values in the 5’-end and the 3’-end was more variable than other domain regions. The differences in the two sliding-window analyses are probably due to the limited number of sequences sampled in the data set of entire sequences, since the 10 species with entire sequences might simply not include the ones that happened to contain the variants leading to the ω peaks found in the larger data set of partial sequences.

The signature of positive selection in the *PHYE* across the *Primulina* phylogeny suggests that phytochromes might have been involved in adaptation to local light environments for *Primulina* species in Karst cave habitats. This novel explanation for plant adaption may yield insights into the species richness and endemism of cave plants in Karst regions. However, to better understand the involvement of phytochromes in local adaptation, other members of phytochrome gene family should be included in future works.

## Supporting Information

S1 Table The species name, sampling sites and GenBank accession numbers used in this study. (DOCX)

S1 File Alignment of 74 partial sequences of *PHYE*. (PDF)

S2 File Alignment of 10 full-length sequences of *PHYE*. (PDF)
