# Supplementary material for: Adaptive Molecular Evolution of PHYE in Primulina, a Karst Cave Plant
Source: PLoS One. 2015 Jun 1;10(6):e0127821. doi: 10.1371/journal.pone.0127821 (PMC4452542; doi:10.1371/journal.pone.0127821)
Supplement: S1 Table — (DOCX) [file pone.0127821.s001.docx]

**Table S1.** **The species name, sampling sites and GenBank accession numbers used in this study.**

| Species name | Coordinates | GenBank accession number | |
| --- | --- | --- | --- |
|  |  | *PHYE* (partial) | *PHYE* (full-length) |
| *P. bicolor* | 24°46'N/111°16'E | KJ028139 |  |
| *P. bipinnatifida* | 25°02'N/110°20'E | KJ028177 |  |
| *P. chizhouensis* | 29°48'N/117°62'E | KJ028164 |  |
| *P. cordata* | 24°77'N/110°46'E | KJ028119 |  |
| *P. cordifolia* | 24°74'N/109°66'E | KJ028147 |  |
| *P. danxiaensis* | 25°04'N/113°75'E | KJ028150 |  |
| *P. depressa* | 26°11'N/113°15'E | KJ028151 |  |
| *P. dongguanica* | 23°64'N/114°34'E | KJ028125 |  |
| *P.* *eburnea* | 29°10'N/107°21'E | KJ028161 | KJ028186 |
| *P. fimbrisepala* | 24°76'N/114°22'E | KJ028128 | KJ028192 |
| *P. glandulosa* | 24°53'N/112°85'E | KJ028124 |  |
| *P. gueilinensis var. brachycarpa* | 26°32'N/116°83'E | KJ028153 |  |
| *P. guihaiensis* | 24°72'N/110°50'E | KJ028182 |  |
| *P. heterotricha* | 18°70'N/109°80'E | KJ028174 | KJ028191 |
| *P. hochiensis* | 24°67'N/108°06'E | KJ028127 |  |
| *P. huaijiensis* | 23°96'N/112°02'E | KJ028189 | KJ028189 |
| *P. langshanica* | 26°09'N/110°64'E | KJ028166 |  |
| *P. latinervis* | 26°74'N/111°12'E | KJ028163 |  |
| *P. laxiflora* | 24°25'N/106°62'E | KJ028173 |  |
| *P. leiophylla* | 23°93'N/106°63'E | KJ028142 |  |
| *P. leprosa* | 23°59'N/108°32'E | KJ028181 |  |
| *P. liguliformis* | 24°69'N/107°84'E | KJ028141 |  |
| *P. lijiangensis* | 24°91'N/110°53'E | KJ028135 |  |
| *P. linearifolia* | 22°91'N/107°92'E | KJ028168 |  |
| *P. lobulata* | 24°12'N/112°77'E | KJ028158 |  |
| *P. longii* | 25°11'N/109°74'E | KJ028118 |  |
| *P. lunglinensis* | 25°14'N/104°95'E | KJ028116 |  |
| *P. lungzhouensis* | 22°89'N/106°36'E | KJ028123 |  |
| *P. luochengensis* | 24°84'N/108°54'E | KJ028185 |  |
| *P. lutea* | 24°39'N/111°55'E | KJ028190 | KJ028190 |
| *P. mabaensis* | 24°67'N/113°58'E | KJ028159 |  |
| *P. macrodonta* | 25°45'N/110°23'E | KJ028138 |  |
| *P. medica* | 24°47'N/110°16'E | KJ028121 |  |
| *P. moii* | 23°34'N/114°17'E | KJ028171 |  |
| *P. mollifolia* | 23°11'N/105°99'E | KJ028184 |  |
| *P. napoensis* | 23°00'N/106°67'E | KJ028143 |  |
| *P. obtusidentata* | 27°84'N/108°77'E | KJ028132 |  |
| *P. orthandra* | 24°18'N/112°56'E | KJ028179 |  |
| *P. parvifolia* | 23°08'N/109°40'E | KJ028148 |  |
| *P. pinnatifida* | 24°98'N/112°89'E | KJ028133 |  |
| *P. pterppoda* | 19°00'N/109°15'E | KJ028188 | KJ028188 |
| *P. pulchurfolia* | 22°36'N/107°10'E | KJ028167 |  |
| *P. renifolia* | 24°34'N/107°91'E | KJ028172 |  |
| *P. repanda* | 25°03'N/109°63'E | KJ028146 |  |
| *P. ronganensis* | 25°02'N/109°34'E | KJ028145 |  |
| *P. sclerophylla* | 24°34'N/107°91'E | KJ028180 |  |
| *P. sinensis* | 22°57'N/114°21'E | KJ028134 | KM403159 |
| *P. spinulosa* | 22°55'N/107°78'E | KJ028169 |  |
| *P. subrhomboidea* | 25°11'N/110°45'E | KJ028117 |  |
| *P. subrhomboidea var. tribracteata* | 23°10'N/106°42'E | KJ028183 |  |
| *P. subulata* | 23°03'N/112°00'E | KJ028122 |  |
| *P. swinglei* | 22°82'N/110°65'E | KJ028187 | KJ028187 |
| *P. tabacum* | 23°17'N/113°36'E | KJ028193 | KJ028193 |
| *P. tenuifolia* | 24°30'N/107°10'E | KJ028115 |  |
| *P. tenuituba* | 26°35'N/107°48'E | KJ028165 |  |
| *P. tiandengensis* | 22°92'N/106°97'E | KJ028144 |  |
| *P. tribracteata* | 24°39'N/107°07'E | KJ028140 |  |
| *P. verecunda* | 23°96'N/110°12'E | KJ028129 |  |
| *P. villosissima* | 23°08'N/112°48'E | KJ028130 | KM403160 |
| *P. wentsaii* | 23°36'N/107°10'E | KJ028170 |  |
| *P. xiuningensis* | 28°62'N/118°59'E | KJ028155 |  |
| *P. xizii* | 30°22'N/120°15'E | KJ028162 |  |
| *P. yangchunensis* | 22°44'N/111°94'E | KJ028137 |  |
| *P. yongxingensis* | 26°12'N/113°14'E | KJ028152 |  |
| *P. yungfuensis* | 25°02'N/110°20'E | KJ028131 |  |
| *P. sp.6* | 23°73'N/111°97'E | KJ028120 |  |
| *P. sp.8* | 25°08'N/113°44'E | KJ028160 |  |
| *P. sp.9* | 24°03'N/113°36'E | KJ028178 |  |
| *P. sp.10* | 24°52'N/114°76'E | KJ028157 |  |
| *P. sp.12* | 29°03'N/117°47'E | KJ028156 |  |
| *P. sp.13* | 25°04'N/113°73'E | KJ028149 |  |
| *P. sp.16* | 26°34'N/117°65'E | KJ028154 |  |
| *P. sp.18* | 24°17'N/112°93'E | KJ028126 |  |
| *P. sp.19* | 25°63'N/110°64'E | KJ028136 |  |
| *Didymocarpus hancei* | 25°29'N/113°06'E | KJ028176 |  |
| *Petrocodon dealbatus* | 25°39'N/113°19'E | KJ028175 |  |
