# Supplementary material for: Adaptive Molecular Evolution of PHYE in Primulina, a Karst Cave Plant
Source: PLoS One. 2015 Jun 1;10(6):e0127821. doi: 10.1371/journal.pone.0127821 (PMC4452542; doi:10.1371/journal.pone.0127821)
Supplement: S1 File — (PDF) [file pone.0127821.s002.pdf]

S2. Alignment of 74 partial sequences of *PHYE*.

	10	20	30	40	50	60
P. bicolor	CAGTACAATG	CCGATGCGAG	GCTGATGGCT	GAATTTGAGC	AGTCCGGTAA	GTCTGGTAAG
P. bipinnatifida	CAGTACAATG	CCGATGCGAG	GCTGATGGCT	GAATTTGAGC	AGTCCGGTAA	GTCTGGTAAG
P. chizhouensis	CAGTACAATG	CCGATGCGAG	GCTGATGGCT	GAATTTGAGC	AG--------	-TCCGGTAAG
P. cordata	CAGTACAATG	CCGATGCGAG	GCTGATGGCT	GAATTTGAGC	AGTCCGGTAA	GTCTGGTAAG
P. depressa	CAGTACAATG	CCGATGCGAG	GCTGATGGCT	GAATTTGAGC	AGTCCGGTAA	GTCTGGTAAG
P. dongguanica	CAGTACAATG	CCGATGCGAG	GCTGATGGCT	GAATTTGAGC	AGTCCGGTAA	GTCTGGTAAG
P. eburnea	CAGTACAATG	CCGATGCGAG	GCTGATGGCT	GAATTTGAGC	AGTCCGGTAA	GTCTGGTAAG
P. fimbrisepala	CAATACAATG	CCGATGCGAG	GCTGATGGCT	GAATTTGAGC	AGTCCGGTAA	GTCTGGTAAG
P. glandulosa var. yangshuoens	CAGTACAATG	CCGATGCGAG	GCTGATGGCT	GAATTTGAGC	AGTCCGGTAA	GTCTGGTAAG
P. gueilinensis var. brachycar	CAGTACAATG	CCGACGCGAG	GCTGATGGCT	GAATTTGAGC	AGTCCGATAA	GTCTGGTAAG
P.guihaiensis	CAGTACAATG	CCGATGCGAG	GCTGATGGCT	GAATTTGAGC	AGTCCGGTAA	GTCTGGTAAG
P. heterotricha & P. pterppoda	CAGTACAATG	CCGATGCGAG	GCTGATGGCT	GAATTTGAGC	AGTCCGGTAA	GTCGGGTAAG
P. hochiensis	CAGTACAATG	CCGATGCGAG	GCTGATGGCT	GAATTTGAGC	AGTCCGGCAA	GTCTGGTAAG
P. huaijiensis	CAGTACAATG	CCGATGCGAG	GCTGATGGCT	GAATTTGAGC	AGTCCGGTAA	GTCTGGTAAG
P. langshanica	CAGTACAATG	CCGATGCGAG	GCTGATGGCT	GAATTTGAGC	AGTCCGGTAA	GTCTGGTAAG
P. latinervis	CAGTACAATG	CCGATGCGAG	GCTGATGGCT	GAATTTGAGC	AGTCCGGTAA	GTCTGGTAAG
P. laxiflora	CAGTACAATG	CCGATGCGAG	GCTGATGGCT	GAATTTGAGC	TGTCCGGTAA	GTCGGGTAAG
P. leprosa	CAGTACAATG	CCGATGCGAG	GCTGATGGCT	GAATTTGAGC	AGTCCGGTAA	GTCTGGTAAG
P. liguliformis	CAGTACAATG	CCGATGCGAG	GCTGATGGCT	GAATTTGAGC	AGTCCGGTAA	GTCTGGTAAG
P. lijiangensis	CAGTACAATG	CCGATGCGAG	GCTGATGGCT	GAATTTGAGC	AGTCCGGTAA	GTCTGGTAAG
P. linearifolia	CAGTACAATG	CCGATGCGAG	GCTGATGGCT	GAATTCGAGC	AGTCCGGTAA	GTCTGGTAAG
P. lobulata	CAGTACAATG	CCGATGCGAG	GCTGATGGCT	GAATTTGAG-	--------AA	GTCTGGTAAG
P. longii	CAGTACAATG	CCGATGCGAG	GCTGATGGCT	GAATTTGAGC	AGTCCGGTAA	GTCTGGTAAG
P. lunglinensis	CAGTACAATG	CCGATGCGAG	GCTGATGGCT	GAATTTGAGC	AGTCCGGTAA	GTCTGGTAAG
P. lungzhouensis	CAGTACAATG	CCGATGCGAG	GCTGATGGCT	GAATTTGAGC	AGTCCGGTAA	GTCTGGTAAG
P. luochengensis	CAGTACAATG	CCGATGCGAG	GCTGATGGCT	GAATTCGAGC	AGTCCGGTAA	GTCTGGTAAG
P. lutea	CAGTACAATG	CCGATGCGAG	GCTGATGGCT	GAATTTGAGC	AGTCTGGTAA	GTCTGGTAAG
P. mabaensis	CAGTACAATG	CCGATGCGAG	GCTGATGGCT	GAATTTGAGC	AGTCCGGTAA	GTCTGGTAAG
P. macrodonta	CAGTACAATG	CCGATGCGAG	GCTGATGGCT	GAATTTGAGC	AGTCCGGTAA	GTCTGGTAAG
P. medica	CAGTACAATG	CCGATGCGAG	GCTGATGGCT	GAATTTGAGC	AGTCCGGTAA	GTCTGGTAAG
P. moii	CAGTACAATG	CCGATGCGAG	GCTGATGGCT	GAATTTGAGC	AGTCCGGTAA	GTCTGGTAAG
P. mollifolia	CAGTACAATG	CCGATGCGAG	GCTGATGGCT	GAATTTGAGC	AGTCCGGTAA	GTCTGGTAAG
P. obtusidentata	CAGTACAATG	CCGATGCGAG	GCTGATGGCT	GAATTTGAGC	AGTCCGGTAA	GTCTGGTAAG
P. orthandra	CAGTACAATG	CCGATGCGAG	GCTGATGGCT	GAATTTGAGC	AGTCCGGTAA	GTCTGGTAAG
P. parvifolia	CAGTACAACG	CCGATGCGAG	GCTGATGGCT	GAATTTGAGC	AGTCCGGTAA	GTCTGGTAAG
P. pinnatifida	CAGTACAATG	CCGATGCGAG	GCTGATGGCT	GAATTTGAGC	AGTCCGGTAA	GTCTGGTAAG
P. pulchurfolia	CAGTACAATG	CCGATGCGAG	GCTGATGGCT	GAATTTGAGC	AGTCCGGTAA	GTCTGGTAAG
P. renifolia	CAGTACAATG	CCGATGCGAG	GCTGATGGCT	GAATTTGAGC	AGTCCGGTAA	GTCTGGTAAG
P. repanda	CAGTACAACG	CCGATGCGAG	GCTGATGGCT	GAATTTGAGC	AGTCCGGTAA	GTCTGGTAAG
P. ronganensis	CAGTACAATG	CCGATGCGAG	GCTGATGGCT	GAATTTGAGC	AGTCCGGTAA	GTCTGGTAAG
P. sclerophylla	CAGTACAATG	CCGATGCGAG	GCTGATGGCT	GAATTTGAGC	AGTCCGGTAA	GTCTGGTAAG
P. sinensis	CAGTACAATG	CCGATGCGAG	GCTGATGGCT	GAATTTGAGC	AGTCCGGTAA	GTCTGGTAAG
P. spinulosa	CAGTACAATG	CCGATGCGAG	GCTTATGGCT	GAATTTGAGC	AGTCCGGTAA	GTCTGGTAAG
P. subrhomboidea	CAGTACAATG	CCGATGCGAG	GCTGATGGCT	GAATTTGAGC	AGTCCGGTAA	GTCTGGTAAG
P. subrhomboidea var. tribract	CAGTACAATG	CCGATGCGAG	GCTGATGGCT	GAATTTGAGC	AGTCCGGTAA	GTCTGGTAAG
P. subulata	CAATACAATG	CCGATGCGAG	GCTGATGGCT	GAATTTGAGC	AGTCTGGTAA	GTCTGGTAAG
P. swinglei	CAGTACAATG	CCGATGCGAG	GCTGATGGCT	GAATTTGAGC	AGTCCGGTAA	GTCAGGTAAG
P. tabacum	CAGTACAATG	CCGATGCGAG	GCTGATGGCT	GAATTTGAGC	AGTCTGGTAA	GTCTGGTAAG
P. tenuifolia	CAGTACAATG	CCGATGCGAG	GCTGATGGCT	GAATTTGAGC	AGTCCGGTAA	GTCTGGTAAG
P. tenuituba	CAGTACAATG	CCGATGCGAG	GCTGATGGCT	GAATTTGAGC	AGTCCGGTAA	GTCTGGTAAG
P. tiandengensis	CAGTACAATG	CCGATGCGAG	GCTGATGGCT	GAATTTGAGC	AGTCCGGTAA	GTCTGGTAAG
P. tribracteata	CAGTACAATG	CCGATGCGAG	GCTGATGGCT	GAATTTGAGC	AGTCCGGTAA	GTCTGGTAAG
P. villosissima	CAGTACAATG	CCGATGCGAG	GCTGATGGCT	GAATTTGAGC	AGTCCGGTAA	GTCTGGTAAG
P. wentsaii	CAGTACAATG	CCGATGCGAG	GCTTATGGCT	GAATTTGAGC	AGTCCGGTAA	GTCTGGTAAG
P. xiuningensis	CAGTACAATG	CCGATGCGAG	GCTGATGGCT	GAATTTGAGC	AGTCCGGTAA	GTCTGGTAAG
P. xizii	CAGTACAATG	CCGATGCGAG	GCTGATGGCT	GAATTTGAGC	AGTCCGGTAA	GTCTGGTAAG
P. yangchunensis	CAGTACAATG	CCGATGCGAG	GCTGATGGCT	GAATTTGAGC	AGTCCGGTAA	GTCTGGTAAG
P. yongxingensis	CAGTACAATG	CCGACGCGAG	GCTGATGGCT	GAATTTGAGC	AGTCCGGTAA	GTCTGGTAAG
P. yungfuensis	CAGTACAATG	CCGATGCGAG	GCTGATGGCT	GAATTTGAGC	AGTCCGGTAA	GTCTGGTAAG
P. cordifolia	CAGTACAACG	CCGATGCGAG	GCTGATGGCT	GAATTTGAGC	AGTCCGGTAA	GTCTGGTAAG
P. danxiaensis	CAGTACAATG	CCGATGCGAG	GCTGATGGCT	GAATTTGAGC	AGTCCGGTAA	GTCTGGTAAG
P. leiophylla & P. napoensis	CAGTACAATG	CCGATGCGAG	GCTGATGGCT	GAATTTGAGC	AGTCCGGTAA	GTCTGGTAAG
P. verecunda	CAATATAATG	CCGATGCGAG	GCTGATGGCT	GAATTTGAGC	AGTCCGGTAA	GTCTGGTAAG
sp. nov. 6	CAGTACAATG	CCGATGCGAG	GCTGATGGCT	GAATTTGAGC	AGTCCGGTAA	GTCTGGTAAG
sp. nov. 8	CAGTACAATG	CCGATGCGAG	GCTGATGGCT	GAATTTGAGC	AGTCCGGTAA	GTCTGGTAAG
sp. nov. 9	CAGTACAATG	CCGATGCGAG	GCTGATGGCT	GAATTTGAGC	AGTCCGGTAA	GTCTGGTAAG
sp. nov. 10	CAGTACAATG	CCGATGCGAG	GCTGATGGCT	GAATTTGAGC	AGTCCGGTAA	GTCTGGTAAG
sp. nov. 12	CAGTACAATG	CCGATGCGAG	GCTGATGGCT	GAATTTGAGG	AGTCCGGTAA	GTCTGGTAAG
sp. nov. 13	CAGTACAATG	CCGATGCGAG	GCTGATGGCT	GAATTTGAGC	AGTCCGGTAA	GTCTGGTAAG
sp. nov. 16	CAGTACAATG	CCGATGCGAG	GCTGATGGCT	GAATTTGAGC	AGTCCGGTAA	GTCTGGTAAG
sp. nov. 18	CAGTACAATG	CCGATGCGAG	GCTGATGGCT	GAATTTGAGC	AGTCCGGTAA	GTCTGGTAAG
sp. nov. 19	CAGTACAATG	CCGATGCGAG	GCTGATGGCT	GAATTTGAGC	AGTCCGGTAA	GTCTGGTAAG
Didymocarpus hancei	CAGTACAATG	CCGATGCGAG	GCTGATGGCT	GAATTTGAGC	AGTCCGGTAA	GTCTGGTAAG
Petrocodon dealbatus	CAGTACAATG	CCGATGCGAG	GCTGATGGCT	GAATTTGAGC	AGTCCGGTAA	GTCTGGTAAG

	61–70	71–80	81–90	91–100	101–110	111–120
	70	80	90	100	110	120
P. bicolor	TTCTTTAACT	ACTCAAAGTC	AGTTTCTCAT	GCTCCGAATA	CTTTGAGCAC	TGAGGAGGAG
P. bipinnatifida	TTCTTCAACT	ACTCAAAGTC	AGTTTCTCAT	GCTCTGAATA	CTTTGAGCAC	TGAGGAGGAG
P. chizhouensis	TTCTTTAACT	ACTCAAAGTC	AGTTTCTCAT	GCTCCGAATA	CTTTGAGCAC	TGAGGAGGAG
P. cordata	TTCTTTAACT	ACTCAAAGTC	AGTTTCTCAT	GCTCCGAATA	CTTTGAGCAC	TGAGGAGGAG
P. depressa	TTCTTTAACT	ACTCAAAGTC	AGTTTCTCAT	GCTCCGAATA	CTTTGAGCAC	TGAGGAGGAG
P. dongguanica	TTCTTTAACT	ACGCAAAGTC	AGTTTCTCAT	GCTCCGAATA	CTTTGAGCAC	TGAGGAGGAG
P. eburnea	TTCTTTAACT	ACTCAAAGTC	AGTTTCTCAT	GCTCCGAATA	CTTTGAGCAC	TGAGGAGGAG
P. fimbrisepala	TTCTTTAACT	ACTCAAAGTC	AGTTTCTCAT	GCTCCGAATA	CTTTGAGCAC	TGAGGAGGAG
P. glandulosa var. yangshuoens	TTCTTTAACT	ACTCAAAGTC	AGTTTCTCAT	GCTCCGAATA	CTTTGAGCAC	TGAGGAGGAG
P. gueilinensis var. brachycar	TTCTTTAACT	ACTCAAAGTC	AGTTTCTCAT	GCTCCGAATA	CTTTGAGCAC	TGAGGAGGAG
P.guihaiensis	TTCTTTAACT	ACTCAAAGTC	AGTTTCTCAT	GCTCCGAATA	CTTTGAGCAC	TGAGGAGGAG
P. heterotricha & P. pterppoda	TTCTTTAACT	ACTCAAAGTC	AGTTTCTCAT	GCTCCGAATA	CTTTGAGCAC	TGAGGAGGAG
P. hochiensis	TTCTTTAACT	ACTCAAAGTC	AGTTTCTCAT	GCTCCGAATA	CTTTGAGCAC	TGAGGAGGAG
P. huaijiensis	TTCTTTAACT	ACTCAAAGTC	AGTTTCTCAT	GCTCCGAATA	CTTTGAGCAC	TGAGGAGGAG
P. langshanica	TTCTTTAACT	ACTCAAAGTC	AGTTTCACAT	GCTCCGAATA	CTTTGAGCAC	TGAGGAGGAG
P. latinervis	TTCTTTAACT	ACTCAAAGTC	AGTTTCTCAT	GCTCCGAATA	CTTTGAGCAC	TGAGGAGGAG
P. laxiflora	TTCTTTAACT	ACTCAAAGTC	AGTTTCTCAT	GCTCCGAATA	CTTTGAGCAC	TGAGGAGGAG
P. leprosa	TTCTTTAACT	ACTCAAAGTC	AGTTTCTCAT	GCTCCGAATA	CTTTGAGCAC	TGAGGAGGAG
P. liguliformis	TTCTTTAACT	ACTCAAAGTC	AGTTTCTCAT	GCTCCGAATA	CTTTGAGCAC	CGAGGAGGAG
P. lijiangensis	TTCTTTAACT	ACTCAAAGTC	AGTTTCTCAT	GCTCCGAATA	CTTTGAGCAC	TGAGGAGGAG
P. linearifolia	TTCTTTAACT	ACTCGAAGTC	AGTTTCTCAT	GCTCCGAATA	CTTTGAGCAC	TGAGGAGGAG
P. lobulata	TTCTTTAACT	ACTCAAAGTC	AGTTTCTCAT	GCTCCGAATA	CTTTGAGCAC	TGAGGAGGAG
P. longii	TTCTTTAACT	ACTCAAAGTC	AGTTTCTCAT	GCTCCGAATA	CTTTGAGCAC	TGAGGAGGAG
P. lunglinensis	TTCTTTAACT	ACTCAAAGTC	AGTTTCTCAT	GCTCCGAATA	CTTTGAGCAC	TGAGGAGGAG
P. lungzhouensis	TTCTTTAACT	ACTCGAAGTC	AGTTTCTCAT	GCTCCGAATA	CTTTGAGCAC	TGAGGAGGAG
P. luochengensis	TTCTTTAACT	ACTCGAAGTC	AGTTTTTCAT	GCTCCGAATA	CTTTGAGCAC	TGAGGAGGAG
P. lutea	TTCTTTAACT	ACTCAAAGTC	AGTTTCTCAT	GCTCCGAATA	CTTTGAGCAC	TGAGGAGGAG
P. mabaensis	TTCTTTAACT	ACTCAAAGTC	AGTTTCTCAT	GCTCCGAATA	CTTTGAGCAC	TGAGGAGGAG
P. macrodonta	TTCTTTAACT	ACTCAAAGTC	AGTTTCTCGT	GCTCCGAATA	CTTTGAGAAC	TGAGGAGGAG
P. medica	TTCTTTAACT	ACTCAAAGTC	AGTTTCTCAT	GCTCCGAATA	CTTTGAGCAC	TGAGGAGGAG
P. moii	TTCTTTAACT	ACTCAAAGTC	AGTTTCTCAT	GCTCCGAATA	CTTTGAGCAC	TGAGGAGGAG
P. mollifolia	TTCTTTAACT	ACTCAAAGTC	AGTTTCTCGT	GCTCCGAATA	CTTTGAGCAC	CGAGGAGGAG
P. obtusidentata	TTCTTTAACT	ACTCAAAGTC	AGTTTCTCAT	GCTCCGAATA	CTTTGAGCAC	TGAGGAGGAG
P. orthandra	TTCTTTAACT	ACTCGAAGTC	AGTTTCTCAT	GCTCCGAATA	CTTTGAGCAC	TGAGGAGGAG
P. parvifolia	TTCTTTAACT	ACTCAAAGTC	AGTTTCTCAT	GCTCCGAATA	CTTTGAGCAC	TGAGGAGGAG
P. pinnatifida	TTCTTTAACT	ACTCAAAGTC	AGTTTCTCAT	GCTCCGAATA	CTTTGAGCAC	TGAGGAGGAG
P. pulchurfolia	TTCTTTAACT	ACTCAAAGTC	AGTTTCTCAT	GCTCCGAATA	CTTTGAGCAC	TGAGGAGGAG
P. renifolia	TTCTTTAACT	ACTCAAAGTC	AGTTTCTCAT	GCTCCGAATA	CTTTGAGCAC	CGAGGAGGAG
P. repanda	TTCTTTAACT	ACTCAAAGTC	AGTTTCTCAT	GCTCCGAATA	CTTTGAGCAC	TGAGGAGGAG
P. ronganensis	TTCTTTAACT	ACTCAAAGTC	AGTTTCTCGT	GCTCCGAATA	CTTTGAGCAC	CGAGGAGGAG
P. sclerophylla	TTCTTTAACT	ACTCAAAGTC	AGTTTCTCAT	GCTCCGAATA	CTTTGAGCAC	CGAGGAGGAG
P. sinensis	TTCTTTAACT	ACTCTAAGTC	AGTTTCTCAT	GCTCCGAATA	CTTTGAGCAC	TGAGGAGGAG
P. spinulosa	TTCTTTAACT	ACTCAAAGTC	AGTTTCTCAT	GCTCCGAATA	CTTTGAGCAC	TGAGGAGGAG
P. subrhomboidea	TTCTTTAACT	ACTCAAAGTC	AGTTTCTCAT	GCTCCGAATA	CTTTGAGCAC	TGAGGAGGAG
P. subrhomboidea var. tribract	TTCTTTAACT	ACTCAAAGTC	AGTTTCTCAT	GCTCCGAATA	CTTTGAGCAC	TGAGGAGGAG
P. subulata	TTCTTTAACT	ACTCAAAGTC	AGTTTCTCAT	GCTCCGAATA	CTTTGAGCAC	TGAGGAGGAG
P. swinglei	TTCTTTAACT	ACTCGAAGTC	AGTTTCTCAT	GCTCCCAATA	CTTTGAGCAC	TGAGGAGGAG
P. tabacum	TTCTTTAACT	ACTCAAAGTC	AGTTTCTCAT	GCTCCGAATA	CTTTGAGCAC	TGAGGAGGAG
P. tenuifolia	TTCTTTAACT	ACTCAAAGTC	AGTTTCTCAT	GCTCCGAATA	CTTTGAGCAC	TGAGGAGGAG
P. tenuituba	TTCTTTAACT	ACTCAAAGTC	AGTTTCTCAT	GCTCCGAATA	CTTTGAGCAC	TGAGGAGGAG
P. tiandengensis	TTCTTTAACT	ACTCAAAGTC	AGTTTCTCAT	GCTCCGAATA	CTTTGAGCAC	CGAGGAGGAG
P. tribracteata	TTCTTTAACT	ACTCAAAGTC	AGTTTCTCAT	GCTCCGAATA	CTTTGAGCAC	CGAGGAGGAG
P. villosissima	TTCTTTAACT	ACTCAAAGTC	AGTTTCTCAT	GCTCCGAATA	CTTTGAGCAC	TGAGGAGGAG
P. wentsaii	TTCTTTAACT	ACTCAAAGTC	AGTTTCTCAT	GCTCCGAATA	CTTTGAGCAC	TGAGGAGGAG
P. xiuningensis	TTCTTTAACT	ACTCAAAGTC	AGTTTCTCAT	GCTCCGAATA	CTTTGAGCAC	TGAGGAGGAG
P. xizii	TTCTTTAACT	ACTCAAAGTC	AGTTTCTCAT	GCTCCGAATA	CTTTGAGCAC	TGAGGAGGAG
P. yangchunensis	TTCTTTAACT	ACTCGAAGTC	AGTTTCTCAT	GCTCCGAATA	CTTTGAGCAC	TGAGGAGGAG
P. yongxingensis	TTCTTTAACT	ACTCAAAGTC	AGTTTCTCAT	GCTCCGAATA	CTTTGAGCAC	TGAGGAGGAG
P. yungfuensis	TTCTTTAACT	ACTCAAAGTC	AGTTTCTCAT	GCTCCGAATA	CTTTGAGCAC	TGAGGAGGAG
P. cordifolia	TTCTTTAACT	ACTCAAAGTC	AGTTTCTCAT	GCTCCGAATA	CTTTGAGCAC	TGAGGAGGAG
P. danxiaensis	TTCTTTAACT	ACTCAATGTC	AGTTTCTCAT	GCTCCGAATA	CTTTGAGCAC	TGAGGAGGAG
P. leiophylla & P. napoensis	TTCTTTAACT	ACTCAAAGTC	AGTTTCTCGT	GCTCCGAATA	CTTTGAGCAC	CGAGGAGGAG
P. verecunda	TTCTTTAACT	ACTCAAAGTC	AGTTTCTCAT	GCTCCGAATA	CTTTGAGCAC	TGAGGAGGAG
sp. nov. 6	TTCTTTAACT	ACTCAAAGTC	AGTTTCTCAT	GCTCCGAATA	CTTTGAGCAC	TGAGGAGGAG
sp. nov. 8	TTCTTTAACT	ACTCAAAGTC	AGTTTCTCAT	GCTCCGAATA	CTTTGAGCAC	TGAGGAGGAG
sp. nov. 9	TTCTTTAACT	ACTCAAAGTC	AGTTTCTCAT	GCTCCGAATA	CTTTGAGCAC	TGAGGAGGAG
sp. nov. 10	TTCTTTAACT	ACTCAAAGTC	AGTTTCTCAT	GCTCCGAATA	CTTTGAGCAC	TGAGGAGGAG
sp. nov. 12	TTCTTTAACT	ACTCAAAGTC	AGTTTCTCAT	GCTCCGAATA	CTTTGAGCAC	TGAGGAGGAG
sp. nov. 13	TTCTTTAACT	ACTCAAAGTC	AGTTTCTCAT	GCTCCGAATA	CTTTGAGCAC	TGAGGAGGAG
sp. nov. 16	TTCTTTAACT	ACTCAAAGTC	AGTTTCTCAT	GCTCCGAATA	CTTTGAGCAC	TGAGGAGGAG
sp. nov. 18	TTCTTTAACT	ACTCAAAGTC	AGTTTCTCAT	GCTCCGAATA	CTTTGAGCAC	TGAGGAGGAG
sp. nov. 19	TTCTTTAACT	ACTCTAAGTC	AGTTTCTCAT	GCTCCGAATA	CTTTGAGCAC	TGAGGAGGAG
Didymocarpus hancei	TTCTTTAACT	ACTCAAAGTC	AGTTTCTCAT	GCTCCGAATA	CTTTGAGCAC	CGAGGAGGAG
Petrocodon dealbatus	TTCTTTAACT	ACTCAAAGTC	AGTTTCTCAT	GCTCCGAATA	CTTTGAGCAC	TGAGGAGGAG

	121–130	131–140	141–150	151–160	161–170	171–180
P. bicolor	ATGACTGCTT	ATTTGTCGAA	AATCCAGAGG	GGGAGTCTTG	TGCAGGCCTT	TGGTTGTATG
P. bipinnatifida	ATGACTGCTT	ATTTGTCGAA	AATCCAGAGG	GGGAGTCTTG	TGCAGGCCTT	TGGTTGTATG
P. chizhouensis	ATGACTGCTT	ATTTGTCGAA	AATCCAGAGG	GGGAGTCTTG	TGCAGGCCTT	TGGTTGTATG
P. cordata	ATGACTGCTT	ATTTGTCGAA	AATCCAGAGG	GGGAGTCTTG	TGCAGGCCTT	TGGTTGTATG
P. depressa	ATGACTGCTT	ATTTGTCGAA	AATCCAGAGG	GGGAGTCTTG	TGCAGGCCTT	TGGTTGTATG
P. dongguanica	ATGATTGCTT	ATTTGTCGAA	AATCCAGAGG	GGGAGTCTTG	TGCAGGCCTT	TGGTTGTATG
P. eburnea	ATGACTGCTT	ATTTATCGAA	AATCCAGAGG	GGGAGTCTTG	TGCAGGCCTT	TGGTTGTATG
P. fimbrisepala	ATGATTGCTT	ATTTGTCGAA	AATCCAGAGG	GGGAGTCTTG	TGCAGGCCTT	TGGTTGTATG
P. glandulosa var. yangshuoens	ATGACTGCTT	ATTTGTCGAA	AATCCAGAGG	GGGAGTCTTG	TGCAGGCCTT	TGGTTGTATG
P. gueilinensis var. brachycar	ATGACTGCTT	ATTTGTCGAA	AATCCAGAGG	GGGAGTCTTG	TGCAGGCCTT	TGGTTGTATG
P.guihaiensis	ATGACTGCTT	ATTTGTCGAA	AATCCAGAGG	GGGAGTCTTG	TGCAGGCCTT	TGGTTGTATG
P. heterotricha & P. pterppoda	ATGACTGCTT	ATTTGTCGAA	AATCCAGAGG	GGGAGTCTTG	TGCAGGCCTT	TGGTTGTATG
P. hochiensis	ATGATTGCTT	ATTTGTCGAA	AATCCAGAGG	GGGAGTCTTG	TGCAGGCCTT	TGGTTGTATG
P. huaijiensis	ATGACTGCTT	ATTTGTCGAA	AATCCAGAGG	GGGAGTCTTG	TGCAGGCCTT	TGGTTGTATG
P. langshanica	ATGACTGCTT	ATCTGTCGAA	AATCCAGAGG	GGGAGTCTTG	TGCAGGCCTT	TGGTTGTATG
P. latinervis	ATGACTGCTT	ATTTGTCGAA	AATCCAGAGG	GGGAGTCTTG	TGCAGGCCTT	TGGTTGTATG
P. laxiflora	ATGACTGCTT	ATTTGTCGAA	AATCCAGAGG	GGGAGTCTTG	TGCAGGCCTT	TGGTTGTATG
P. leprosa	ATGACTGCTT	ATTTGTCGAA	AATCCAAAGG	GGGAGTCTTG	TGCAGGCCTT	CGGTTGTATG
P. liguliformis	ATGACTGCTT	ATTTGTCGAA	AATCCAGAGG	GGGAGTCTTG	TGCAGGCCTT	TGGTTGTATG
P. lijiangensis	ATGACTGCTT	ATTTGTCGAA	AATCCAGAGG	GGGAGTCTTG	TGCAGGCCTT	TGGTTGTATG
P. linearifolia	ATGACTGCTT	ATTTGTCGAA	AATCCAGAGG	GGGAGTCTTG	TGCAGGCCTT	TGGTTGTATG
P. lobulata	ATGACTGCTT	ATTTGTCGAA	AATCCAGAGG	GGGAGTCTTG	TGCAGGCCTT	TGGTTGTATG
P. longii	ATGACTGCTT	ATTTGTCGAA	AATCCAGAGG	GGGAGTCTTG	TGCAGGCCTT	TGGTTGTATG
P. lunglinensis	ATGACTGCTT	ATTTGTCGAA	AATCCAGAGG	GGGAGTCTTG	TGCAGGCCTT	TGGTTGTATG
P. lungzhouensis	ATGACTGCTT	ATTTGTCGAA	AATCCAGAGG	GGGAGTCTTG	TGCAGGCCTT	TGGTTGTATG
P. luochengensis	ATGACTGCTT	ATTTGTCGAA	AATCCAGAGG	GGGAGTCTTG	TGCAGGCCTT	TGGTTGTATG
P. lutea	ATGACTGCTT	ATTTGTCGAA	AATCCAGAGG	GGGAGTCTTG	TGCAGGCCTT	TGGTTGTATG
P. mabaensis	ATGACTGCTT	ATTTGTCGAA	AATCCAGAGG	GGGAGTCTTG	TGCAGGCCTT	TGGTTGTATG
P. macrodonta	ATGACTGCTT	ATTTGTCGAA	AATCCAGAGG	GGGAGTCTTG	TGCAGGCCTT	TGGTTGTATG
P. medica	ATGACTGCTT	ATTTGTCGAA	AATCCAGAGG	GGGAGTCTTG	TGCAGGCCTT	TGGTTGTATG
P. moii	ATGACTGCTT	ATTTGTCGAA	AATCCAGAGG	GGGAGTCTTG	TGCAGGCCTT	TGGTTGTATG
P. mollifolia	ATGACTGCTT	ATTTGTCGAA	AATCCAGAGG	GGGAGTCTTG	TGCAGGCCTT	TGGTTGTATG
P. obtusidentata	ATGACTGCTT	ATTTGTCGAA	AATCCAGAGG	GGGAGTCTTG	TGCAGGCCTT	TGGTTGTATG
P. orthandra	ATGACTGCTT	ATTTGTCGAA	AATCCAGAGG	GGGAGTCTTG	TGCAGGCCTT	TGGTTGTATG
P. parvifolia	ATGACTGCTT	ATTTGTCGAA	AATCCAGAGG	GGGAGTCTTG	TGCAGGCCTT	TGGTTGTATG
P. pinnatifida	ATGACTGCTT	ATTTGTCGAA	AATCCAGAGG	GGGAGTCTTG	TGCAGGCCTT	TGGTTGTATG
P. pulchurfolia	ATGACTGCTT	ATTTGTCGAA	AATCCAGAGG	GGGAGTCTTG	TGCAGGCCTT	TGGTTGTATG
P. renifolia	ATGACTGCTT	ATTTGTCGAA	AATCCAGAGG	GGGAGTCTTG	TGCAGGCCTT	TGGTTGTATG
P. repanda	ATGACTGCTT	ATTTGTCGAA	AATCCAGAGG	GGGAGTCTTG	TGCAGGCCTT	TGGTTGTATG
P. ronganensis	ATGACTGCTT	ATTTGTCGAA	AATCCAGAGG	GGGAGTCTTG	TGCAGGCCTT	TGGTTGTATG
P. sclerophylla	ATGACTGCTT	ATTTGTCGAA	AATCCAGAGG	GGGAGTCTTG	TGCAGGCCTT	TGGTTGTATG
P. sinensis	ATGACTGCTT	ATTTGTCGAA	AATCCAGAGG	GGGAGTCTTG	TGCAGGCCTT	TGGTTGTATG
P. spinulosa	ATGACTGCTT	ATTTGTCGAA	AATCCAGAGG	GGGAGTCTTG	TGCAGGCCTT	TGGTTGTATG
P. subrhomboidea	ATGACTGCTT	ATTTGTCGAA	AATCCAGAGG	GGGAGTCTTG	TGCAGGCCTT	TGGTTGTATG
P. subrhomboidea var. tribract	ATGACTGCTT	ATTTGTCGAA	AATCCAGAGG	GGGAGTCTTG	TGCAGGCCTT	TGGTTGTATG
P. subulata	ATGACTGCTT	ATTTGTCGAA	AATCCAGAGG	GGGAGTCTTG	TGCAGGCCTT	TGGTTGTATG
P. swinglei	ATGACTGCTT	ATTTGTCGAA	AATCCAGAGG	GGAAGTCTTG	TTCAGGCCAT	TGGTTGTATG
P. tabacum	ATGACTGCTT	ATTTGTCGAA	AATCCAGAGG	GGGAGTCTTG	TGCAGGCCTT	TGGTTGTATG
P. tenuifolia	ATGACTGCTT	ATTTGTCGAA	AATCCAGAGG	GGGAGTCTTG	TGCAGGCCTT	TGGTTGTATG
P. tenuituba	ATGACTGCTT	ATTTGTCGAA	AATCCAGAGG	GGGAGTCTTG	TGCAGGCCTT	TGGTTGTATG
P. tiandengensis	ATGACTGCTT	ATTTGTCGAA	AATCCAGAGG	GGGAGTCTTG	TGCAGGCCTT	TGGTTGTATG
P. tribracteata	ATGACTGCTT	ATTTGTCGAA	AATCCAGAGG	GGGAGTCTTG	TGCAGGCCTT	TGGTTGTATG
P. villosissima	ATGATTGCTT	ATTTGTCGAA	AATCCAGAGG	GGGAGTCTTG	TGCAGGCCTT	TGGTTGTATG
P. wentsaii	ATGACTGCTT	ATTTGTCGAA	AATCCAGAGG	GGGAGTCTTG	TGCAGGCCTT	TGGTTGTATG
P. xiuningensis	ATGACTGCTT	ATTTGTCGAA	AATCCAGAGG	GGGAGTCTTG	TGCAGGCCTT	TGGTTGTATG
P. xizii	ATGACTGCTT	ATTTGTCGAA	AATCCAGAGG	GGGAGTCTTG	TGCAGGCCTT	TGGTTGTATG
P. yangchunensis	ATGACTGCTT	ATTTGTCGAA	AATCCAGAGG	GGGAGTCTTG	TGCAGGCCTT	TGGTTGTATG
P. yongxingensis	ATGACTGCTT	ATTTGTCGAA	AATCCAGAGG	GGGAGTCTTG	TGCAGGCCTT	TGGTTGTATG
P. yungfuensis	ATGACTGCTT	ATTTGTCGAA	AATCCAGAGG	GGGAGTCTTG	TGCAGGCCTT	TGGTTGTATG
P. cordifolia	ATGACTGCTT	ATTTGTCGAA	AATCCAGAGG	GGGAGTCTTG	TGCAGGCCTT	TGGTTGTATG
P. danxiaensis	ATGACTGCTT	ATTTGTCGAA	AATCCAGAGG	GGGAGTCTTG	TGCAGGCCTT	TGGTTGTATG
P. leiophylla & P. napoensis	ATGACTGCTT	ATTTGTCGAA	AATCCAGAGG	GGGAGTCTTG	TGCAGGCCTT	TGGTTGTATG
P. verecunda	ATGACTGCTT	ATTTGTCGAA	AATCCAGAGG	GGGAGTCTTG	TGCAGGCCTT	TGGTTGTATG
sp. nov. 6	ATGACTGCTT	ATTTGTCGAA	AATCCAGAGG	GGGAGTCTTG	TGCAGGCCTT	TGGTTGTATG
sp. nov. 8	ATGACTGCTT	ATTTGTCGAA	AATCCAGAGG	GGCAGTCTTG	TGCAGGCCTT	TGGTTGTATG
sp. nov. 9	ATGACTGCTT	ATTTGTCGAA	AATCCAGAGG	GGCAGTCTTG	TGCAGGCCTT	TGGTTGTATG
sp. nov. 10	ATGACTGCTT	ATTTGTCGAA	AATCCAGAGG	GGAAGTCTTG	TGCAGGCCTT	TGGTTGTATG
sp. nov. 12	ATGACTGCTT	ATTTGTCGAA	AATCCAGAGG	GGGAGTCTTG	TGCAGGCCTT	TGGTTGTATG
sp. nov. 13	ATGACTGCTT	ATTTGTCGAA	AATCCAGAGG	GGGAGTCTTG	TGCAGGCCTT	TGGTTGTATG
sp. nov. 16	ATGACTGCTT	ATTTGTCGAA	AATCCAAAGG	GGGAGTCTTG	TGCAGGCCTT	TGGTTGTATG
sp. nov. 18	ATGATTGCTT	ATTTGTCGAA	AATCCAGAGG	GGGAGTCTTG	TGCAGGCCTT	TGGTTGTATG
sp. nov. 19	ATGACTGCCT	ATTTGTCGAA	AATCCAGAGG	GGGAGTCTTG	TGCAGGCCTT	TGGTTGTATG
Didymocarpus hancei	ATGACTGCTT	ATTTGTCGAA	AATCCAGAGG	GGGAGTCTTG	TGCAGGCCTT	TGGTTGTATG
Petrocodon dealbatus	ATGACTGCTT	ATTTGTCGAA	AATCCAGAGG	GGGAGTCTTG	TGCAGGCCTT	TGGTTGTATG

	190	200	210	220	230	240
P. bicolor	CTTGTGGTCG	AGGAGCCCAG	TTTGAAGATC	ATAGGTTATA	GTGAGAATTG	CTTTGATATG
P. bipinnatifida	CTTGCGGTCG	AGGAACCCAG	TTTGAAGATC	ATAGGTTATA	GTGAGAATTG	CTTTGATATG
P. chizhouensis	CTTGCGGTCG	AGGAACCCAG	TTTGAAGATC	ATAGGTTATA	GTGAGAATTG	CTTTGATATG
P. cordata	CTTGCGGTCG	AGGAGCCCAG	TTTGAAGATC	ATAGGTTATA	GTGAGAATTG	CTTTGATATG
P. depressa	CTTGCGGTCG	AGGAGCCCAG	TTTGAAGATC	ATAGGTTATA	GTGAGAATTG	CTTTGATATG
P. dongguanica	CTTGCGGTCG	AGGAGCCCAG	TTTGAAGATC	ATAGGTTATA	GTGAGAATTG	CTTTGATATG
P. eburnea	CTTGCGGTCG	AGGAGCCCAG	TTTGAAGATC	ATAGGTTATA	GTGAGAATAT	CTTTGATATG
P. fimbrisepala	CTTGCGGTCG	AGGAGCCCAG	TTTGAAGATC	ATAGGTTATA	GTGAGAATTG	CTTTGATATG
P. glandulosa var. yangshuoens	CTTGCGGTCG	AGGAGCCCAG	TTTGAAGATC	ATAGGTTATA	GTGAGAATTG	TTTTGATATG
P. gueilinensis var. brachycar	CTTGCGCTCG	AGGAGCCCAG	TTTGAAGATC	ATAGGTTATA	GTGAGAATTG	CTTTGATATG
P.guihaiensis	CTTGCGGTCG	AGGAGCCCAG	TTTGAAGATC	ATAGGTCACA	GTGAGAATTG	CTTTGATATG
P. heterotricha & P. pterppoda	CTTGCGGTCG	AGGAGCCCAG	TTTGAAGATC	ATAGGTTATA	GTGAGAATTG	CTTTGATATG
P. hochiensis	CTTGCGGTCG	AGGAGCCCAG	TTTGAAGATC	ATAGGTCATA	GTGAGAATTG	CTTTGATATG
P. huaijiensis	CTTGCGGTCG	AGGAGCCCAG	TTTGAAGATC	ATAGGTTATA	GTGAGAATTG	CTTTGATATG
P. langshanica	CTTGCGGTCG	AGGAGCCCAG	TTTGAAGATC	ATAGGTTATA	GCGAGAATTG	CTGTGATATG
P. latinervis	CTCGCGGTCG	AGGAGCCCAG	TTTGAAGATC	ATAGGTTATA	GTGAGAATTG	CTTTGATATG
P. laxiflora	CTTGCGGTCG	AGGAGCCCAG	TTTGAAGATC	ATAGGTTATA	GTGAGAATTG	CTTTGATATG
P. leprosa	CTTGCGGTCG	AGGAGCCCAG	TTTGAAGATC	ATAGGTTATA	GTGAGAATTG	CTTTGATATG
P. liguliformis	CTTGCGGTCG	AGGAGCCCAG	TTTGAAGATC	ATAGGTTATA	GTGAGAATTG	CTTTGATATA
P. lijiangensis	CTTGCGGTCG	AGGAGCCCAG	TTTGAAGATC	ATAGGTTATA	GTGAGAATTG	CTTTGATATG
P. linearifolia	CTTGCGGTCG	AGGAGCCCAG	TTTGAAGATC	ATAGGTTATA	GTGAGAATTG	CTTTGATATG
P. lobulata	CTTGCGGTCG	AGGAGCCCAG	TTTGAAGATC	ATAGGTTATA	GTGAGAATTG	CTTTGATATG
P. longii	CTTGCGGTCG	AGGAGCCCAG	TTTGAAGATC	ATAGGTCATA	GTGAGAATTG	CTTTGATATG
P. lunglinensis	CTTGCGGTCG	AGGAGCCCAG	TTTGAAGATC	ATAGGTCATA	GTGAGAATTG	CTTTGATATG
P. lungzhouensis	CTTGCGGTCG	AGGAGCCCAG	TTTGAGGATC	ATAGGTTATA	GTGAGAATTG	CTTTGATATG
P. luochengensis	CTTGCGGTCG	AGGAGCCCAG	TTTGAAGATC	ATAGGTTATA	GTGAGAATTG	CTTTGATATG
P. lutea	CTTGCGGTCG	AGGAGCCCAG	TTTGAAGATC	ATAGGTTATA	GTGAGAATTG	CTTTGATATG
P. mabaensis	CTTGCGGTCG	AGGAGCCCAG	TTTGAAGATC	ATAGGTTATA	GTGAGAATTG	CTTTGATATG
P. macrodonta	CTTGCGGTCG	AGGAGCCCAG	TTTGAAGATC	ATAGGTTATA	GTGAGAATTG	CTTTGATATG
P. medica	CTTGCGGTCG	AGGAGCCCAG	TTTGAAGATC	ATAGGTTATA	GTGAGAATTG	CTTTGATATG
P. moii	CTTGCGGTCG	AGGAGCCCAG	TTTGAAGATC	ATAGGTTATA	GTGAGAATTG	CTTTGATATG
P. mollifolia	CTTGCGGTCG	AGGAGCCCAG	TTTGAAGATC	ATAGGTTATA	GTGAGAATTG	CTTTGATATG
P. obtusidentata	CTTGCGGTCG	AGGAGCCCAG	TTTGAAGATC	ATAGGTTATA	GTGAGAATTG	CTTTGATACG
P. orthandra	CTTGCGGTCG	AGGAGCCCAG	TTTGAAGATC	ATAGGTTATA	GTGAGAATTG	CTTTGATATG
P. parvifolia	CTTGCGGTCG	AGGAGCCCAG	TTTGAAGATC	ATAGGTTATA	GTGAGAATTG	CTTTGATATG
P. pinnatifida	CTTGCGGTCG	AGGAGCCCAG	TTTGAAGATC	ATAGGTTATA	GTGAGAATTG	CTTTGATATG
P. pulchurfolia	CTTGCGGTCG	AGGAGCCCAG	TTTGAAGATC	ATAGGTTATA	GTGAGAATTG	CTTTGATATG
P. renifolia	CTTGCGGTCG	AGGAGCCCAG	TTTGAAGATC	ATAGGTTATA	GTGAGAATTG	CTTTGATATG
P. repanda	CTTGCGGTCG	AGGAGCCCAG	TTTGAAGATC	ATAGGTTATA	GTGAGAATTG	CTTTGATATG
P. ronganensis	CTTGCGGTCG	AGGAGCCCAG	TTTGAAGATC	ATAGGTTACA	GTGAGAATTG	CTTTGATATG
P. sclerophylla	CTTGCGGTCG	AGGAGCCCAG	TTTGAAGATC	ATAGGTTATA	GTGAGAATTG	CTTTGATATG
P. sinensis	CTTGCGGTCG	AGGAGCCCAG	TTTGAAGATC	ATAGGTTATA	GTGAGAATTG	CTTTGATATG
P. spinulosa	CTTGCGGTCG	AGGAGCCCAG	TTTGAAGATC	GTAGGTTATA	GTGAGAATTG	CTTTGATATG
P. subrhomboidea	CTTGCGGTCG	AGGAGCCCAG	TTTGAAGATC	ATAGGTCATA	GTGAGAATTG	CTTTGATATG
P. subrhomboidea var. tribract	CTTGCGGTCG	AGGAGCCCAG	TTTGAAGATC	ATAGGTTATA	GTGAGAATTG	CTTTGATATG
P. subulata	GTTGCGGTCG	AGGAGCCCAG	TTTGAAGATC	ATAGGTTATA	GTGAGAATTG	CTTTGATATG
P. swinglei	CTTGCGGTCG	AGGAGCCCAG	TTTAAAGATC	ATAGGTTATA	GTGAGAATTG	CTTTGATATG
P. tabacum	CTTGCGGTCG	AGGAGCCCAG	TTTGAAGATT	ATAGGTTATA	GTGAGAATTG	CTTTGATATG
P. tenuifolia	CTTGCGGTCG	AGGAGCCCAG	TTTGAAGATC	ATAGGTCATA	GTGAGAATTG	CTTTGATATG
P. tenuituba	CTTGCGGTCG	AGGAGCCCAG	TTTGAAGATC	ATAGGTTATA	GTGAGAATTG	CTTTGATATG
P. tiandengensis	CTTGCGGTCG	AGGAGCCCAG	TTTGAAGATC	ATAGGTTATA	GTGAGAATTG	CTTTGATATG
P. tribracteata	CTTGCGGTCG	AGGAGCCCAG	TTTGAAGATC	ATAGGTTATA	GTGAGAATTG	CTTTGATATG
P. villosissima	CTTGCGGTCG	AGGAGCCCAG	TTTGAAGATC	ATAGGTTATA	GTGAGAATTG	CTTTGATATG
P. wentsaii	CTTGCGGTTG	AGGAGCCCAG	TTTGAAGATC	ATAGGTTATA	GTGAGAATTG	CTTTGATATG
P. xiuningensis	CTTGCGGTCG	AGGAGCCCAG	TTTGAAGATC	ATAGGTTATA	GTGAGAATTG	CTTTGATATG
P. xizii	CTTGCGGTCG	AGGAGCCCAG	TTTGAAGATC	ATAGGTTATA	GTGAGAATTG	CTTTGATATG
P. yangchunensis	CTTGCGGTCG	AGGAGCCCAG	TTTGAAGATC	ATAGGTTATA	GTGAGAATTG	CTTTGATATG
P. yongxingensis	CTTGCGGTCG	AGGAGCCCAG	TTTGAAGATC	ATAGGTTATA	GTGAGAATTG	CTTTGATATG
P. yungfuensis	CTTGCGGTCG	AGGAGCCCAG	TTTGAAGATC	ATAGGTTATA	GTGAGAATTG	CTTTGATATG
P. cordifolia	CTTGCGGTCG	AGGAGCCCAG	TTTGAAGATC	ATAGGTTATA	GTGAGAATTG	CTTTGATATG
P. danxiaensis	CTTGCGGTCG	AGGAGCCCAG	TTTGAAAATC	ATAGGTTATA	GTGAGAATTG	CTTTGATATG
P. leiophylla & P. napoensis	CTTGCGGTCG	AGGAGCCCAG	TTTGAAGATC	ATAGGTTATA	GTGAGAATTG	CTTTGATATG
P. verecunda	CTTGCCGTCG	AGGAGCCCAG	TTTGAAGATC	ATAGGTTATA	GTGAGAATTG	CTTTGATATG
sp. nov. 6	CTTGCGGTCG	AGGAGCCCAG	TTTGAAGATC	ATAGGTCATA	GTGAGAATTG	CTTTGATATG
sp. nov. 8	CTTGCGGTCG	AGGAGCCCAG	TTTGAAGATC	ATAGGTTATA	GTGAGAATTG	CTTTGATATG
sp. nov. 9	CTTGCGGTCG	AGGAGCCCAG	TTTGAAGATC	ATAGGTTATA	GTGAGAATTG	CTTTGATATG
sp. nov. 10	CTTGCGGTCG	AGGAGCCCAG	TTTGAAGATC	ATAGGTTATA	GTGAGAATTG	CTTTGATATG
sp. nov. 12	CTTGCGGTCG	AGGAGCCCAG	TTTGAAGATC	ATAGGTTATA	GTGAGAATTG	CTTTGATATG
sp. nov. 13	CTTGCGGTCG	AGGAGCCCAG	TTTGAAGATC	ATAGGTTATA	GTGAGAATTG	CTTTGATATG
sp. nov. 16	CTTGCGGTCG	AGGAGCCCAG	TTTGAAGATC	ATAGGTTATA	GTGAGAATTG	CTTTGATATG
sp. nov. 18	CTTGCGGTCG	AGGAGCCCAG	TTTGAAGATC	ATAGGTTATA	GTGAGAATTG	CTTTGATATG
sp. nov. 19	CTTGCGGTCG	AGGAGCCCAG	TTTGAAGATC	ATAGGTTATA	GTGAGAATTG	CTTTGATATG
Didymocarpus hancei	CTTGCGGTCG	AGGAGCCCAG	TTTTAAGATC	ATAGGTTATA	GTGAGAATTG	CGTTGATATG
Petrocodon dealbatus	CTTGCGGTCG	AGGAGCCCAG	TTTTAAGATC	ATAGGTTATA	GTGAGAATTG	CGTTGATATG

	250	260	270	280	290	300
P. bicolor	CTGGGCTTGA	AGAGTGTCGT	CGAGCCAAAA	AAATTGATGG	GTCTTATTGG	GGTTGATGCA
P. bipinnatifida	CTGGGTTTGA	AGAGCGTCGT	TGAGCCAAAA	AAATTGATGG	GTCTTATTGG	GGTTGATGCA
P. chizhouensis	CTGGGCTTGA	AGAGTGTCGT	TGAGCCAAAA	AAATTGATGG	GTCTTATTGG	GGTTGATGCA
P. cordata	CTGGGTTTGA	AGAGCGTCGT	TGAGCAAAAA	AAATTGATGG	GTCTTATTGG	GGTTGATGCA
P. depressa	CTGGGCTTGA	AGAGTGTCGT	TGATCCAAAA	AAATTGATGG	GTCTTATTGG	GGTTGATGCA
P. dongguanica	CTGGGTTTGA	AGAGCGTCGT	TGAGCCAAAA	AAATGGATGG	GTCTTATTGG	GGTTGATGCA
P. eburnea	CTGGGCTTGA	AAAGGGTCGT	GGAGCCAAAA	AAATTGATGG	GTCTTATTGG	GGTTGATGCA
P. fimbrisepala	CTGGGTTTGA	AGAGCGTCGT	CGAACCAAAA	AAATTGATGG	GTCTTATTGG	GGTTGATGCA
P. glandulosa var. yangshuoens	CTGGGCTTGA	AGAGTGTAGT	TGAGCCAAAA	AAATGGATGG	GTCTTATTGG	GGTTGATGCA
P. gueilinensis var. brachycar	CTGGGGTTGA	AGAGTGTCGT	TGAGCCAAAA	AAATTGATGG	GTCTTATTGG	GGTTGATGCA
P.guihaiensis	CTGGGTTTGA	AGAGCGTCGT	TGAGCCAAAA	AAATTGATGG	GTCTTATTGG	GGTTGATGCA
P. heterotricha & P. pterppoda	CTGGGTTTGA	AGAGTGTCGT	TGAGCCAAAA	AAATTGATGG	GTCTTCTTGG	GGTTGATGCA
P. hochiensis	CTGGGTTTGA	AGAGTGTCGT	TGAGCCAAAA	AAATTGATGG	GTCTTATTGG	GGTTGATGCA
P. huaijiensis	CTGGGTTTGA	AGAGCGTCGT	TGAGCCAAAA	AAATTGATGG	GTCTTATTGG	GGTTGATGCA
P. langshanica	CTGGGCTTGA	AGAGTGTCGT	TGAGCCAAAA	AAATTGATGG	GTCTTATTGG	GGTTGATGCA
P. latinervis	CTGGGCTTGA	AGAGTGTCGT	TGAGCCAAAA	AAAATGATGG	GTCTTATTGG	GGTTGATGCA
P. laxiflora	CTGGGTTTGA	AGAGTGTTGT	TGAGCCAAAA	AAATTGATGG	GTCTTATTGG	GGTTGATGCA
P. leprosa	CTGGGTTTGA	AGAGCGTCGT	TGAGCCAAAA	AAATTGATGG	GTCTTATTGG	GGTTGATGCA
P. liguliformis	CTGGGTTTGA	AGAGTGTTGT	TGAGCCAAAA	AAATTGACGG	GTCTTATTGG	GGTTGATGCA
P. lijiangensis	CTGGGTTTGA	AGAGCGTCGT	TGAGCCAAAA	AAATTGATGG	GTCTTATTGG	GGTTGATGCA
P. linearifolia	CTGGGTTTGA	AGAGTGTCGT	TGAGCCAAAA	AAATTGATGG	GTCTTATTGG	GGTTGATGCA
P. lobulata	CTGGGCTTGA	AGAGTGTCGT	TGATCCAAAA	AAATTGATGG	GTCTTATTGG	GGTTGATGCA
P. longii	CTGGGTTTGA	AGAGCGTCGT	TGAGCCAAAA	AAATTGATGG	GTCTTATTGG	GGTTGATGCA
P. lunglinensis	CTGGGTTTGA	AGAGCGTCGT	TGAGCCAAAA	AAATTGGTGG	GTCTTATTGG	GGTTGATGCA
P. lungzhouensis	CTGGGCTTGA	AGAGTTTAGT	TGAGCCAAAA	AAATTGATGG	GTCTTATTGG	GGTTGATGCA
P. luochengensis	CTGGGTTTGA	AGAGTGTCGT	TGAGCCAAAA	AAATTGATGG	GTCTTATTGG	GGTTGATGCA
P. lutea	CTGGGCTTGA	AGAGTGTCGT	TGAGCCAAAA	AAATTGATGG	GTCTTATTGG	GGTTGATGCA
P. mabaensis	CTGGGATTGA	AGAGTGTCGT	TGATCCAAAA	AAATTGATGG	GTCTTATTGG	GGTTGATGCA
P. macrodonta	CTGGGTTTGA	AGAGTGTTGT	TGAGCCAAAA	AAATTGTCGG	GTCTTATTGG	GGTTGATGCA
P. medica	CTGGGTTTGA	AGAGCGTCGT	TGAGCCAAAA	AAATTGATGG	GTCTTATTGG	GGTTGATGCA
P. moii	CTGGGCTTGA	AGAATGTCGT	TGAGCCAAAA	AAATTGATGG	GTCTTATTGG	GGTTGATGCA
P. mollifolia	CTGGGTTTGA	AGAGTGTTGT	TGAGCCAAAA	AAATTGACGG	GTCTTGTTGG	GGTTGATGCA
P. obtusidentata	CTGGGTTTGA	AGAGCGTCGT	TGAGCCAAAA	AAATTGATGG	GTCTTATTGG	AGTTGATGCA
P. orthandra	CTGGGCTTGA	AGAGTGTCGT	CGAGCCAAAA	AAATTGATGG	GTCTTATTGG	GGTTGATGCA
P. parvifolia	CTGGGTTTGA	AGAGTGTTGT	TGAGCCAAAA	AAATTGATGG	GTCTTATTGG	GGTTGATGCA
P. pinnatifida	CTGGGTTTGA	AGAGCGTCGT	TGAGCCAAAA	AAATTGATGG	GTCTTATTGG	GGTTGATGCA
P. pulchurfolia	CTGGGTTTGA	AGAGTGTCGT	TGAGCCAAAA	AAATTGATGG	GTCTTATTGG	GGTTGATGCA
P. renifolia	CTGGGTTTGA	AGAGTGTTGT	TGAGCCAAAA	AAATTGACGG	GTCTTATTGG	GGTTGATGCA
P. repanda	CTGGGTTTGA	AGAGTGTTGT	TGAGCCAAAA	AAATTGACGG	GTCTTATTGG	GGTTGATGCA
P. ronganensis	CTGGGTTTGA	AGAGTGTTGT	TGAGCCAAAA	AAATTGACGG	GTCTTATTGG	GGTTGATGCA
P. sclerophylla	CTGGGTTTGA	AGAGTGTTGT	TGAGCCAAAA	AAATTGACGG	GTCTTATTGG	GGTTGATGCA
P. sinensis	CTGGGTTTGA	AGAGCGTCGT	TGAGCCAAAA	AAATTGATGG	GTCTTATTGG	GGTTGATGCA
P. spinulosa	CTGGGTTTGA	AGAGTGTCGT	TGAGCCAAAA	AAATTGATGG	GTCTTATTGG	GGTTGATGCA
P. subrhomboidea	CTGGGTTTGA	AGAGCGTCGT	TGAGCCAAAA	AAATTGATGG	GTCTTATTGG	GGTTGATGCA
P. subrhomboidea var. tribract	CTGGGTTTGA	AGAGTGTCGT	TGAGCCAAAA	AAATTGATGG	GTCTTATTGG	GGTTGATGCA
P. subulata	CTGGGTTTGA	AGAGCGTCGT	TGAGCCAAAA	AAATTGATGG	GACTTATTGG	GGTTGATGCA
P. swinglei	CTGGGTTTGA	AGAGTGTTGT	TGAGCCAAAA	AAATCGATGG	GTCTTATTGG	GGTTGATGCA
P. tabacum	CTGGGGTTGA	AGAGTGTCGT	TGAGCCAAAA	AAATTGATGG	GTCTTATTGG	GGTTGATGCA
P. tenuifolia	CTGGGTTTGA	AGAGCGTCGT	TGAGCCAAAA	AAATTGATGG	GTCTTATTGG	GGTTGATGCA
P. tenuituba	CTGGGCTTGA	AGAGTGTCGT	TGAGCCAAAA	AAATTGATGG	GTCTTATTGG	GGTTGATGCA
P. tiandengensis	CTGGGTTTGA	AGAGTGTTGT	GGAGCCAAAA	AAATTGACGG	GTCTTATTGG	GGTTGATGCA
P. tribracteata	CTGGGTTTGA	AGAGTGTTGT	TGAGCCAAAA	AAATTGACGG	GTCTTATTGG	GGTTGATGCA
P. villosissima	CTGGGTTTGA	AGAGCGTCGT	TGAGCCAAAA	AAATTGATGG	GTCTTATTGG	GGTTGATGCA
P. wentsaii	CTGGGTTTGA	AGAGTGTCGT	TGAGCCAAAA	AAATTGATGG	GTCTTATTGG	GGTTGATGCA
P. xiuningensis	CTGGGATTGA	AGAGTGTCGT	TGATCCAAAA	AAATTTATGG	GTCTTATTGG	GGTTGATGCA
P. xizii	CTGGGCTTGA	AGAGTGTCGT	TGAGCCAAAA	AAATTGATGG	GTCTTATTGG	GGTTGATGCA
P. yangchunensis	CTGGGTTTGA	AGAGCGTCGT	TGAGCCAAAA	AAATTGATGG	GTCTTATTGG	GGTTGATGCA
P. yongxingensis	CTGGGCTTGA	AGAGTGTCGT	TGATCCCAAA	AAATTGATGG	GTCTTATTGG	GGTTGATGCA
P. yungfuensis	CTGGGTTTGA	AGAGCGTCGT	TGAGCCAAAA	AGATTGATGG	GTCTTATTGG	GGTTGATGCA
P. cordifolia	CTGGGTTTGA	AGAGTGTTGT	TGAGCCAAAA	AAATTCACGG	GTCTTATTGG	GGTTGATGCA
P. danxiaensis	CTGGGATTGA	AGAGTGTCGT	TGATCCAAAA	AAATTGATGG	GTCTTATTGG	GGTTGATGCA
P. leiophylla & P. napoensis	CTGGGTTTGA	AGAGTGTTGT	TGAGCCAAAA	AAATTGACGG	GTCTTATTGG	GGTTGATGCA
P. verecunda	CTGGGTTTGA	AGAGCGTCGT	CGAGCCAAAA	AAATTGATGG	GTCTTATTGG	GGTTGATGCA
sp. nov. 6	CTGGGTTTGA	AGAGCGTCGT	TGAGCCAAAA	AAATTGATGG	GTCTTATTGG	GGTTGATGCA
sp. nov. 8	CTGGGCTTGA	AGAGTGTCGT	TGAGCCAAAA	AAATTGACGG	GTCTTATTGG	GGTTGATGCA
sp. nov. 9	CTGGGCTTGA	AGAGTGTCGT	TGAGCCAAAA	AAATTGATGG	GTCTTATTGG	GGTTGATGCA
sp. nov. 10	CTGGGGTTGA	AGAGTGTCGT	TGAGCCAAAA	AAATTGATGG	GTCTTATTGG	GGTTGATGCA
sp. nov. 12	CTGGGATTGA	AGAGTGTCGT	TGATCCAAAA	AAATTGATGG	GTCTTATTGG	GGTTGATGCA
sp. nov. 13	CTGGGATTGA	AGAGTGTCGT	TGATCCAAAA	AAATTGATGG	GTCTTATTGG	GGTTGATGCA
sp. nov. 16	CTGGGATTGA	AGAGTGTCGT	TGATCCAAAA	AAATTGATGG	GTCTTATTGG	GGTTGATGCA
sp. nov. 18	CTGGGCTTGA	AGAGTGTGGT	TGAGCCAAAA	AAATTGATGG	GTCTTATTGG	GGTTGATGCA
sp. nov. 19	CTGGGTTTGA	AGAGCGTCGT	TGAGCCAAAA	AAATTGATGG	GTCTTATTGG	GGTTGATGCA
Didymocarpus hancei	CTTGGTTTGA	AGAGTGTTGT	TGAGCCAAAA	AAATTGATGG	GTCTTATTGG	GGTTGATGCA
Petrocodon dealbatus	CTGGGTTTGA	AGAGTGTTGT	TGAGCCAAAA	AAATTGATGG	GTCTTATTGG	GGTTGATGCA

	310	320	330	340	350	360
P. bicolor	AGAACGCTTT	TTACCTCTTC	AAGTAGGGCT	TCATTGGATA	AAGCTGTGGC	ATCAAGGGAA
P. bipinnatifida	AGAACGCTTT	TTACCTCTTC	AAGTAGGGCT	TCATTGGATA	AAGCTGTGGC	ATCAAGGGAA
P. chizhouensis	AGAACGCTTT	TTACCTCTTC	AAGTAGGGCT	TCATTGGATA	AAGCTGTGGC	ATCAAGGGAA
P. cordata	AGAACGCTTT	TTACCTCTTC	AAGTAGGGCT	TCATTGGATA	AAGCTGTGGC	ATCAAGGGAA
P. depressa	AGGACGCTTT	TTACCTCTTC	AAGTAGGGCT	TCATTGGATA	AAGCTGTGGC	ATCAAGGGAA
P. dongguanica	AGAACGCTTT	TTACCTCTTC	AAGTAGGGCT	TCGTTGGATA	AAGCTGTGGC	ATCAAGGGAA
P. eburnea	AGAACGCTTT	TTACCTCTTC	AAGTAGGGCT	TCATTGGATA	AAGCTGTGGC	ATCCAGGGAA
P. fimbrisepala	AGAACGCTTT	TTACCTCTTC	AAGTAGGGCT	TCATTGGATA	AAGCTGTGGC	ATCAAGGGAA
P. glandulosa var. yangshuoens	AGGACGCTTT	TTACCTCTTC	AAGTAGGGCG	TCATTGGATA	AAGCTGTGGC	ATCAAGGGAA
P. gueilinensis var. brachycar	AGAACGCTTT	TTACCTCTTC	AAGTAGGGCT	TCATTGGATA	AAGCTGTGGC	ATCAAGGGAA
P.guihaiensis	AGAACGCTTT	TTACCTCTTC	AAGTAGGGCT	TCATTGGATA	AAGCTGTGGC	ATCAAGGGAA
P. heterotricha & P. pterppoda	AGAACGCTTT	TTACCTCTTC	AAGTAGGGAT	TCATTGGATA	AAGCTGTGGC	ATCAAGGGAA
P. hochiensis	AGAACGCTTT	TTACCTCTTC	AAGTAGGGCT	TCCTTGGATA	AAGCTGTGGC	ATCAAGGGAA
P. huaijiensis	AGAACGCTTT	TTACCTCTTC	AAGTAGGGCT	TCATTGGATA	AAGCTGTGGC	ATCAAGGGAA
P. langshanica	AGAACGCTTT	TTACCTCTTC	AAGTAGGGCT	TCATTGGATA	AAGCTGTGGC	ATCAAGGGAA
P. latinervis	AGAACGCTTT	TTACCTCTTC	AAGTAGGGCT	TCATTGGACA	AAGCTGTGGC	ATCAAGGGAA
P. laxiflora	AGAACGCTTT	TTACCTCTTC	AAGTAGGGCT	TCATTGGATA	AAGCTGTGGC	ATCAAGGGAA
P. leprosa	AGAACGCTTT	TTACCTCTTC	AAGTAGGGCT	TCATTGGATA	AAGCTGTGGC	ATCAAGGGAA
P. liguliformis	AGAACGCTTT	TTACCTCTTC	AAGTAGGGCT	TCATTGGATA	AAGCTGTGGC	ATCAAGGGAA
P. lijiangensis	AGAACGCTTT	TTACCTCTTC	AAGTAGGGCT	TCATTGGATA	AAGCTGTGGC	ATCAAGGGAA
P. linearifolia	AGAACGCTTT	TTACCTCTTC	AAGTAGGGCT	TCATTGGATA	AAGCTGTGGC	ATCAAGGGAA
P. lobulata	AGAACGCTTT	TTACCTCTTC	AAGTAGGGCT	TCATTGGATA	AAGCTGTGGC	ATCAAGGGAA
P. longii	AGAACGCTTT	TTACCTCTTC	AAGTAGGGCT	TCATTGGATA	AAGCTGTGGC	ATCAAGGGAA
P. lunglinensis	AGATCGCTTT	TTACCTCTTC	AAGTAGGGCT	TCATTGGATA	AAGCTGTGGC	ATCAAGGGAA
P. lungzhouensis	AGAACGCTTT	TTACCTCTTC	AAGTAGGGCT	TCATTGGATA	AAGCTGTGGC	ATCAAGGGAA
P. luochengensis	AGAACGCTTT	TTACCTCTTC	AAGTAGGGCT	TCATTGGATA	AAGCTGTGGC	ATCAAGGGAA
P. lutea	AGAACGCTTT	TTACCTCTTC	AAGTAGGGCT	TCATTGGATA	AAGCTGTGGC	ATCAAGGGAA
P. mabaensis	AGGACGCTTT	TTACCTCTTC	AAGTAGGGCT	TCATTGGATA	AAGCTGTGGC	ATCAAGGGAA
P. macrodonta	AGAACGCTTT	TTACCTCTTC	AAGTAGGGCT	TCATTGGATA	AAGCTGTGGC	ATCAAGGGAA
P. medica	AGGACGCTTT	TTACCTCTTC	AAGTAGGGCT	TCATTGGATA	AAGCTGTGGC	ATCAAGGGAA
P. moii	AGAACGCTTT	TTACCTCTTC	AAGTAGGGCT	TCATTGGATA	AAGCTGTGGC	ATCAAGGGAA
P. mollifolia	AGAACGCTTT	TTACCTCTTC	AAGTAGGGCT	TCATTGGATA	AAGCTGTGGC	ATCAAGGGAA
P. obtusidentata	AGAACGCTTT	TTACCTCTTC	AAGTAGGGCT	TCATTGGATA	AAGCTGTGGC	ATCAAGGGAA
P. orthandra	AGAACTCTTT	TTACCTCTTC	AAGTAGGGCT	TCATTGGATA	AAGCTGTGGC	ATCAAGGGAA
P. parvifolia	AGAACGCTTT	TTACCTCTTC	AAGTAGGGCT	TCATTGGATA	AAGCTGTGGC	ATCAAGGGAA
P. pinnatifida	AGAACGCTTT	TTACCTCTTC	AAGTAGGGCT	TCGTTGGATA	AAGCTGTGGC	ATCAAGGGAA
P. pulchurfolia	AGAACGCTTT	TTACCTCTTC	AAGTAGGGCT	TCATTGGATA	AAGCTGTGGC	ATCAAGGGAA
P. renifolia	AGAACGCTTT	TTACCTCTTC	AAGTAGGGCT	TCATTGGATA	AAGCTGTGGC	ATCAAGGGAA
P. repanda	AGAACGCTTT	TTACCTCTTT	AAGTAGGGCT	TCATTGGATA	AAGCTGTGGC	ATCAAGGGAA
P. ronganensis	AGAACGCTTT	TTACCTCTTC	AAGTAGGGCT	TCATTGGATA	AAGCTGTGGC	ATCAAGGGAA
P. sclerophylla	AGAACGCTTT	TTACCTCTTC	AAGTAGGGCT	TCATTGGATA	AAGCTGTGGC	ATCAAGGGAA
P. sinensis	AGAACGCTTT	TTACCTCTTC	AAGTAGGGCT	TCGTTGGATA	AAGCTGTGGC	ATCAAGGGAA
P. spinulosa	AGAACGCTTT	TTACCGCTTC	AAGTAGGGCT	TCATTGGATA	AAGCTGTGGC	ATCAAGGGAA
P. subrhomboidea	AGAACGCTTT	TTACCTCTTC	AAGTAGGGCT	TCATTGGATA	AAGCTGTGGC	ATCAAGGGAA
P. subrhomboidea var. tribract	ACAACGCTTT	TTACCTCTTC	CAGTAGGGCT	TCATTGGATA	AAGCTGTGGC	ATCAAGGGAA
P. subulata	AGAACGCTTT	TTACCTCTTC	AAGTAGGGCT	TCCTTGGATA	AAGCTGTGGC	ATCAAGGGAA
P. swinglei	AGAACGCTTT	TTACCTCTTC	AAGTAGGGCT	TCGTTGGATA	AAGCTGTGGC	ATCGAGGGAA
P. tabacum	AGAACGCTTT	TTACCTCTTC	AAGTAGGGCT	TCATTGGATA	AAGCTGTGGC	ATCAAGGGAA
P. tenuifolia	AGAACGCTTT	TTACCTCTTC	AAGTAGGGCT	TCATTGGATA	AAGCTGTGGC	ATCAAGGGAA
P. tenuituba	AGAACGCTTT	TTACCTCTTC	AAGTAGGGCT	TCATTGGATA	AAGCTGTGGC	ATCAAGGGAA
P. tiandengensis	AGAACGCTCT	TTACCTCTTC	AAGTAGGGCT	TCATTGGATA	AAGCTGTGGC	ATCAAGGGAA
P. tribracteata	AGAACGCTTT	TTACCTCTTC	AAGTAGGGCT	TCATTGGATA	AAGCTGTGGC	ATCAAGGGAA
P. villosissima	AGAACGCTTT	TTACCTCTTC	AAGTAGGGCT	TCATTGGATA	AAGCTGTGGC	ATCAAGGGAA
P. wentsaii	AGAACGCTTT	TTACCTCTTC	AAGTAGGGCT	TCATTGGATA	AAGCTGTGGC	ATCAAGGGAA
P. xiuningensis	AGGACGCTTT	TTACCTCTTC	GAGTAGGGCT	TCATTGGATA	AAGCTGTGGC	ATCAAGGGAA
P. xizii	AGAACGCTTT	TTACCTCTTC	AAGTAGGGCT	TCATTGGATA	AAGCTGTGGC	ATCAAGGGAA
P. yangchunensis	AGAACGCTTT	TTACCTCTTC	AAGTAGGGCT	TCATTGGATA	AAGCTGTGGC	ATCAAGGGAA
P. yongxingensis	AGAACGCTTT	TTACCTCTTC	GAGCAGGGCT	TCATTGGATA	AAGCTGTGGC	ATCAAGGGAA
P. yungfuensis	AGAACGCTTT	TTACCTCTTC	AAGTAGGGCT	TCGTTGGATA	AAGCTGTGGC	ATCAAGAGAA
P. cordifolia	AGAACACTTT	TTACCTCTTC	AAGTAGGGCT	TCATTGGATA	AAGCTGTGGC	ATCAAGGGAA
P. danxiaensis	AGAACGCTTT	TTACCTCTTC	AAGTAGGGCT	TCATTGGATA	AAGCTGTGGC	ATCAAGGGAA
P. leiophylla & P. napoensis	AGAACGCTTT	TTACCTCTTC	AAGTAGGGCT	TCATTGGATA	AAGCTGTGGC	ATCAAGGGAA
P. verecunda	AGAACGCTTT	TTACCTCTTC	AAGTAGGGCT	TCATTGGATA	AAGCTGTGGC	ATCAAGGGAA
sp. nov. 6	AGAACGCTTT	TTACCTCTTC	AAGTAGGGCT	TCATTGGATA	AAGCTGTGGC	ATCAAGGGAA
sp. nov. 8	AGAACGCTTT	TTACCTCTTC	AAGTAGGGCT	TCATTGGATA	AAGCTGTGGC	ATCAAGGGAA
sp. nov. 9	AGAACGCTTT	TTACCTCTTC	AAGTAGGGCT	TCATTGGATA	AAGCTGTGGC	ATCAAGGGAA
sp. nov. 10	AGAACGCTTT	TTACCTCTTC	AAGTAGGGCT	TCATTGGATA	AAGCTGTGGC	ATCAAGGGAA
sp. nov. 12	AGGACGCTTT	TTACCTCTTC	GAGTAGGGCT	TCATTGGATA	AAGCTGTGGC	ATCAAGGGAA
sp. nov. 13	AGGACGCTTT	TTACCTCTTC	AAGTAGGGCT	TCATTGGATA	AAGCTGTGGC	ATCAAGGGAA
sp. nov. 16	AGAACACTTT	TTACCTCTTC	AAGTAGGGCT	TCATTGGATA	AAGCTGTGGC	ATCAAGGGAA
sp. nov. 18	AGAACGCTTT	TTACCTCTTC	AAGTAGGGCT	TCCTTGGATA	AAGCTGTGGC	ATCAAGGGAA
sp. nov. 19	AGAACGCTTT	TTACCTCTTC	AAGTAGGGCT	TCCTTGGATA	AAGCTGTGGC	ATCAAGGGAA
Didymocarpus hancei	AGAACCCTTT	TTACCTCTTC	ATGTAGGGCT	TCATTGGATA	AAGCTGTGGC	ATCAAGGGAA
Petrocodon dealbatus	AGAACCCTTT	TTACCTCTTC	ATGTAGGGCT	TCATTGGATA	AAGCTGTGGC	ATCAAGGGAA

	370	380	390	400	410	420
P. bicolor	ATTTCATTTT	TGAACCCGAT	TTGGGTTCAT	TCTTGCACCA	CCCACAAACC	ATTTTATGCT
P. bipinnatifida	ATTTCATTTT	TGAACCCGAT	TTGGGTTCAT	TCTTGCACCA	CCCACAAACC	ATTTTATGCT
P. chizhouensis	ATTTCATTTT	TGAACCCGAT	TTGGGTTCAT	TCTTGCACCA	CCCACAAACC	ATTTTATGCT
P. cordata	ATTTCATTTT	TGAACCCGAT	TTGGGTTCAT	TCTTGCACCA	CCCACAAACC	ATTTTATGCT
P. depressa	ATTTCATTTT	TGAACCCGAT	TTGGGTTCAT	TCTTGCACCA	CCCACAAACC	ATTTTATGCT
P. dongguanica	ATTTCATTTT	TGAACCCGAT	TTGGGTTCAT	TCTTGCACCA	CCCACAAACC	ATTTTATGCT
P. eburnea	ATTTCATTTT	TGAACCCGAT	TTGGGTTCAT	TCTTGCACCA	CCCACAAACC	ATTTTATGCT
P. fimbrisepala	ATTTCATTTT	TGAACCCGAT	TTGGGTTCAT	TCTTGCACCA	CCCACAAACC	ATTTTATGCT
P. glandulosa var. yangshuoens	ATTTCATTTT	TGAACCCGAT	TTGGGTTCAT	TCTTGCACCA	CCCACAAACC	ATTTTATGCT
P. gueilinensis var. brachycar	ATTTCATTTT	TGAACCCTAT	TTGGGTTCAT	TCTTGCACCA	CCCACAAACC	ATTTTATGCT
P.guihaiensis	ATTTCATTTT	TGAACCCGAT	TTGGGTTCAT	TCTTGCACCA	CCCACAAACC	ATTTTATGCT
P. heterotricha & P. pterppoda	ATTTCATTTT	TGAACCCGAT	TTGGGTTCAT	TCTTGCACCA	CCCACAAACC	ATTTTATGCT
P. hochiensis	ATTTCATTTT	TGAACCCGAT	TTGGGTTCAT	TCTTGCACCA	CCCACAAACC	ATTTTATGCT
P. huaijiensis	ATTTCATTTT	TGAACCCGAT	TTGGGTTCAT	TCTTACACCA	CCCACAAACC	ATTTTATGCT
P. langshanica	ATTTCATTTT	TGAACCCGAT	TTGGGTTCAT	TCTTGCACCA	CCCACAAACC	ATTTTATGCT
P. latinervis	ATTTCATTTT	TGAACCCGAT	TTGGGTTCAT	TCTTGCACCA	CCCACAAACC	ATTTTATGCT
P. laxiflora	ATTTCATTTT	TGAACCCGAT	TTGGGTTCAT	TCTTGCACCA	CCCACAAACC	ATTTTATGCT
P. leprosa	ATTTCATTTT	TGAACCCGAT	TTGGGTTCAT	TCTTGCACCA	CCCACAAACC	ATTTTATGCT
P. liguliformis	ATTTCATTTT	TGAACCCGAT	TTGGGTTCAT	TCTTGCACCA	ACCACAAACC	ATTTTATGCT
P. lijiangensis	ATTTCATTTT	TGAACCCGAT	TTGGGTTCAT	TCTTGCACCA	CCCACAAACC	ATTTTATGCT
P. linearifolia	ATTTCATTTT	TGAACCCGAT	TTGGGTTCAT	TCTTGCACCA	CCCACAAACC	ATTTTATGCT
P. lobulata	ATTTCATTTT	TGAACCCGAT	TTGGGTTCAT	TCTTGCACCA	CCCACAAACC	ATTTTATGCT
P. longii	ATTTCATTTT	TGAACCCGAT	TTGGGTTCAT	TCTTGCACCA	CCCACAAACC	ATTTTATGCT
P. lunglinensis	ATTTCATTTT	TGAACCCGAT	TTGGGTTCAC	TCTTGCACCA	CCCACAAACC	ATTTTATGCT
P. lungzhouensis	ATTTCATTTT	TGAACCCGAT	TTGGGTTCAT	TCTTGCACCA	CCCACAAACC	ATTCTATGCT
P. luochengensis	ATTTCATTTT	TGAACCCGAT	TTGGGTTCAT	TCTTGCACCA	CCCACAAACC	ATTCTATGCT
P. lutea	ATTTCATTTT	TGAACCCGAT	TTGGGTTCAT	TCTTGCACCA	CCCACAAACC	ATTTTATGCT
P. mabaensis	ATTTCATTTT	TGAACCCGAT	TTGGGTTCAT	TCTTGCACCA	CCCACAAACC	ATTTTATGCT
P. macrodonta	ATTTCATTTT	TGAACCCGAT	TTGGGTTCAT	TCTTGCACCA	ACCACAAACC	ATTTTATGCT
P. medica	ATTTCATTTT	TGAACCCGAT	TTGGGTTCAT	TCTTGCACCA	CCCACAAACC	ATTTTATGCT
P. moii	ATTTCATTTT	TGAACCCGAT	TTGGGTTCAT	TCTTGCACCA	CCCACAAACC	ATTTTATGCT
P. mollifolia	ATTTCATTTT	TGAACCCGAT	TTGGGTTCAT	TCTTGCACCA	ACCACAAACC	ATTTTATGCT
P. obtusidentata	ATTTCGTTTT	TGAACCCGAT	TTGGGTTCAT	TCTTGCACCA	CCCACAAACC	ATTTTATGCT
P. orthandra	ATTTCATTTT	TGAACCCGAT	TTGGGTTCAT	TCTTGCACCA	CCCACAAACC	ATTTTATGCT
P. parvifolia	ATTTCATTTT	TGAACCCGAT	TTGGGTTCAT	TCTTGCACTA	ACCACAAACC	ATTTTATGCT
P. pinnatifida	ATTTCATTTT	TGAACCCGAT	TTGGGTTCAT	TCTTGCACCA	CCCACAAACC	ATTTTATGCT
P. pulchurfolia	ATTTCATTTT	TGAACCCGAT	TTGGGTTCAT	TCTTGCACCA	CCCACAAACC	ATTTTATGCT
P. renifolia	ATTTCATTTT	TGAACCCGAT	TTGGGTTCAT	TCTTGCACTA	ACCACAAACC	ATTTTATGCT
P. repanda	ATTTCATTTT	TGAACCCGAT	TTGGGTTCAT	TCTTGCACTA	ACCATAAACC	ATTTTATGCT
P. ronganensis	ATTTCATTTT	TGAACCCGAT	TTGGGTTCAT	TCTTGCACCA	ACCACAAACC	ATTTTATGCT
P. sclerophylla	ATTTCATTTT	TGAACCCGAT	TTGGGTTCAT	TCTTGCACCA	ACCACAAACC	ATTTTATGCT
P. sinensis	ATTTCATTTT	TGAACCCGAT	TTGGGTTCAT	TCTTGCACCA	CCCACAAACC	ATTTTATGCT
P. spinulosa	ATTTCATTTT	TGAACCCGAT	TTGGGTTCAT	TCTTGCACCA	CCCACAAACC	ATTTTATGCT
P. subrhomboidea	ATTTCATTTT	TGAACCCGAT	TTGGGTTCAT	TCTTGCACCA	CCCACAAACC	ATTTTATGCT
P. subrhomboidea var. tribract	ATTTCATTTT	TGAACCCGAT	CTGGGTTCAT	TCTTGCACCA	CCCACAAACC	ATTTTATGCT
P. subulata	ATTTCATTTT	GGAACCCGAT	TTGGGTTCAT	TCTTGCACCA	CCCACAAACC	ATTTTATGCT
P. swinglei	ATTTCATTTT	TGAACCCGAT	TTGGGTTCAT	TCTTGCACCA	CCCACAAACC	ATTTTATGCT
P. tabacum	ATTTCATTTT	TGAACCCGAT	TTGGGTTCAT	TCTTGCACCA	CCCACAAACC	ATTTTATGCT
P. tenuifolia	ATTTCATTTT	TGAACCCGAT	TTGGGTTCAC	TCTTGCACCA	CCCACAAACC	ATTTTATGCT
P. tenuituba	ATTTCATTTT	TGAACCCGAT	TTGGGTTCAT	TCTTGCACCA	CCCACAAACC	ATTTTATGCT
P. tiandengensis	ATTTCATTTT	TGAACCCGAT	TTGGGTTCAT	TCTTGCACCA	ACCACAAACC	ATTTTATGCT
P. tribracteata	ATTTCATTTT	TGAACCCGAT	TTGGGTTCAT	TCTTGCACCA	ACCACAAACC	ATTTTATGCT
P. villosissima	ATTTCATTTT	TGAACCCGAT	TTGGGTTCAT	TCTTGCACCA	CCCACAAACC	ATTTTATGCT
P. wentsaii	ATTTCATTTT	TGAACCCGAT	TTGGGTTCAT	TCTTGCACCA	CCCACAAACC	ATTTTATGCT
P. xiuningensis	ATTTCATTTT	TGAACCCGAT	TTGGGTTCAT	TCTTGCACCA	CCCACAAACC	ATTTTATGCT
P. xizii	ATTTCATTTT	TGAACCCGAT	TTGGGTTCAT	TCTTGCACCA	CCCACAAACC	ATTTTATGCT
P. yangchunensis	ATTTCATTTT	TGAACCCGAT	TTGGGTTCAT	TCTTGCACCA	CCCACAAACC	ATTTTATGCT
P. yongxingensis	ATTTCATTTT	TGAACCCGAT	TTGGGTTCAT	TCTTGCACCA	CCCACAAACC	ATTTTATGCT
P. yungfuensis	ATTTCATTTT	TGAACCCGAT	TTGGGTTCAT	TCTTGCACCA	CCCACAAACC	ATTTTATGCT
P. cordifolia	ATTTCATTTT	TGAACCCGAT	TTGGGTTCAT	TCTTGCACTA	TCCACAAACC	ATTTTATGCT
P. danxiaensis	ATTTCATTTT	TGAACCCTAT	TTGGGTTCAT	TCTTGCACCA	CCCACAAACC	ATTTTATGCT
P. leiophylla & P. napoensis	ATTTCATTTT	TGAACCCGAT	TTGGGTTCAT	TCTTGCACCA	ACCACAAACC	ATTTTATGCT
P. verecunda	ATTTCATTTT	TGAACCCGAT	TTGGGTTCAT	TCTTGCACCA	CCCACAAACC	ATTTTATGCT
sp. nov. 6	ATTTCATTTT	TGAACCCGAT	TTGGGTTCAT	TCTTGCACCA	CCCACAAACC	ATTTTATGCT
sp. nov. 8	ATTTCATTTT	TGAACCCGAT	TTGGGTTCAT	TCTTGCACCA	CCCACAAACC	ATTTTATGCT
sp. nov. 9	ATTTCATTTT	TGAACCCGAT	TTGGGTTCAT	TCTTGCACCA	CCCACAAACC	ATTTTATGCT
sp. nov. 10	ATTTCATTTT	TGAACCCGAT	TTGGGTTCAT	TCTTGCACCA	CCCACAAACC	ATTTTATGCT
sp. nov. 12	ATTTCATTTT	TGAACCCGAT	TTGGGTTCAT	TCTTGCACCA	CCCACAAACC	ATTTTATGCT
sp. nov. 13	ATTTCATTTT	TGAACCCTAT	TTGGGTTCAT	TCTTGCACCA	CCCACAAACC	ATTTTATGCT
sp. nov. 16	ATTTCATTTT	TGAACCCGAT	TTGGGTTCAT	TCTTGCACCA	CCCACAAACC	ATTTTATGCT
sp. nov. 18	ATTTCATTTT	TGAACCCGAT	TTGGGTTCAT	TCTTGCACCA	CCCACAAACC	ATTTTATGCT
sp. nov. 19	ATTTCATTTT	TGAACCCGAT	TTGGGTTCAT	TCTTGCACCA	CCCACAAACC	ATTTTATGCT
Didymocarpus hancei	ATTTCATTTT	TGAACCCGAT	TTGGGTTCAT	TCTTGCACCA	CCCACAAACC	ATTTTATGCT
Petrocodon dealbatus	ATTTCATTTT	TGAACCCGAT	TTGGGTTCAT	TCTTGCACCA	CCCACAAACC	ATTTTATGCT

	430	440	450	460	470	480
P. bicolor	ATATTACACA	GAATTGATGT	GGGCATTGTG	ATTGATCTGG	AGCCAGCTCG	TGCTTGTGAT
P. bipinnatifida	ATATTACACA	GAATTGATGT	GGGCATTGTG	ATTGATCTGG	AGCCAGCTCG	TGCTTGTGAT
P. chizhouensis	ATATTGCACA	GAATTGACGT	GGGCATTGTG	GTTGATCTGG	AGCCAGCTCG	TGCTTGTGAT
P. cordata	ATATTACACA	GAATTGATGT	GGGCATTGTG	ATTGATCTGG	AGCCAGCTCG	TGCTTGTGAT
P. depressa	ATATTACACA	GAATTGACGT	GGGCATTGTG	GTTGATCTGG	AGCCAGCTCG	TGCTTGTGAT
P. dongguanica	ATATTACACA	GAATTGATGT	GGGCATTGTG	ATTGATCTGG	AGCCAGCTCG	TGCTTGCGAT
P. eburnea	ATATTACACA	GAATTGACGT	GGGCATTGTG	GTTGATCTGG	AGCCAGCTCG	TGCTTGTGAT
P. fimbrisepala	ATATTACACA	GAATTGATGT	GGGCATTGTG	ATTGATCTGG	AGCCAGCTCG	TGCTTGCGAT
P. glandulosa var. yangshuoens	ATATTACACA	GAATTGATGT	GGGCATTGTG	ATTGATCTGG	AGCCAGCTCG	TGCTTGTGAT
P. gueilinensis var. brachycar	ATATTACACA	GAATTGACGT	GGGCATTGTG	GTTGATCTGG	AGCCAGCTCG	TGCTTGTGAT
P.guihaiensis	ATATTACACA	GAATTGATGT	GGGCATTGTG	ATTGATCTGG	AGCCAGCTCG	TGCTTGTGAT
P. heterotricha & P. pterppoda	ATATTACACA	GAATTGATGT	GGGCATTGTG	ATTGATCTGG	AGCCAGCTCG	TGCTTGTGAT
P. hochiensis	ATATTACACA	GAATTGACGT	GGGCATTGTG	ATTGATCTGG	AGCCAGCTCG	TGCTTGCGAT
P. huaijiensis	ATATTACACA	GAATTGACGT	GGGCATTGTG	GTTGATCTGG	AGCCAGCTCG	TGCTTGTGAT
P. langshanica	ATATTACACA	GAATTGACGT	GGGCATTGTG	GTTGATCTGG	AACCAGCTCG	TGCTTGTGAT
P. latinervis	ATATTACACA	GAATTGATGT	GGGCATTGTG	GTTGATCTGG	AGCCAGCTCG	TGCTTGTGAT
P. laxiflora	ATTTTACACA	GAATTGATGT	GGGCATTGTG	ATTGATCTGG	AGCCAGCTCG	TGCTTGTGAT
P. leprosa	ATATTACACA	GAATTGATGT	GGGCATTGTG	ATTGATCTGG	AGCCAGCTCG	TGCTTGTGAT
P. liguliformis	ATATTACACA	GAATTGACGT	GGGCATTGTG	ATTGATCTGG	AGCCAGCTCG	TGCTTGTGAT
P. lijiangensis	ATATTACACA	GAATTGATGT	GGGCATTGTG	ATTGATCTGG	AGCCAGCTCG	TGCTTGTGAT
P. linearifolia	ATATTACACA	GAATTGATGT	GGGCATTGTG	ATTGATCTGG	AGCCAGCTCT	TGCTTGTGAT
P. lobulata	ATATTACACA	GAATTGACGT	GGGCATTGTG	GTTGATCTGG	AGCCAGCTCG	TGCTTGTGAT
P. longii	ATATTACACA	GAATTGATGT	GGGCATTGTG	ATTGATCTGG	AGCCAGCTCG	TGCTTGTGAT
P. lunglinensis	ATATTACACA	GAATTGATGT	GGGCATTGTG	ATTGATCTGG	AGCCAGCTCG	TGCTTGTGAT
P. lungzhouensis	ATATTACACA	GAATTGATGT	GGGCATTGTG	ATTGATCTGG	AGCCAGCTCG	TGCTTGTGAT
P. luochengensis	ATATTACACA	GAATTGATGT	GGGCATTGTG	ATTGATCTGG	AGCCAGCTCG	TGCTTGTGAT
P. lutea	ATATTACACA	GAATTGACGT	GGGCATTGTG	GTTGATCTGG	AGCCAGCTCG	TGCTTGTGAT
P. mabaensis	ATATTACACA	GAATTGACGT	GGGCATTGTG	GTTGATCTGG	AGCCAGCTCG	TGCTTGTGAT
P. macrodonta	ATATTACACA	GAATTGACGT	GGGCATTGTG	ATTGATCTGG	AGCCAGCTCG	TGCTTGTGAT
P. medica	ATATTACACA	GAATTGATGT	GGGCATTGTG	ATTGATCTGG	AGCCAGCTCG	TGCTTGTGAT
P. moii	ATATTACACA	GAATTGATGT	GGGCATTGTG	ATTGATCTGG	AGCCAGCTCG	TGCTTGTGAT
P. mollifolia	ATATTACACA	GAATTGACGT	GGGCATTGTG	ATTGATCTGG	AGCCAGCTCG	TGCTTGTGAT
P. obtusidentata	ATATTACACA	GAATTGATGT	GGGCATTGTG	ATTGATCTGG	AGCCAGCTCG	TGCTTGTGAT
P. orthandra	ATATTACACA	GAATTGACGT	GGGCATTGTG	GTTGATCTGG	AGCCAGCTCG	TGCTTGTGAT
P. parvifolia	ATATTACACA	GAATTGACGT	GGGCATTGTG	ATTGATCTGG	AGCCAGCTCG	TGCTTGTGAT
P. pinnatifida	ATATTACACA	GAATTGATGT	GGGCATTGTG	ATTGATCTGG	AGCCAGCTCG	TGCTTGTGAT
P. pulchurfolia	ATATTACACA	GAATTGATGT	GGGCATTGTG	ATTGATCTGG	AGCCAGCTCG	TGCTTGTGAC
P. renifolia	ATATTACACA	GAATTGACGT	GGGCATTGTG	ATTGATCTGG	AGCCAGCTCG	TGCTTGTGAT
P. repanda	ATATTACACA	GAATTGACGT	GGGCATTGTG	ATTGATCTGG	AGCCAGCTCG	TGCTTGTGAT
P. ronganensis	ATATTACACA	GAATTGACGT	GGGCATTGTG	ATTGATCTGG	AGCCAGCTCG	TGCTTGTGAT
P. sclerophylla	ATATTACACA	GAATTGACGT	GGGCATTGTG	ATTGATCTGG	AGCCAGCTCG	TGCTTGTGAT
P. sinensis	ATATTACACA	GAATTGATGT	GGGCATTGTG	ATTGATCTGG	AGCCAGCTCG	TGCTTGTGAT
P. spinulosa	ATATTACACA	GAATTGATGT	GGGCATTGTG	ATTGATCTGG	AGCCAGCTCG	TGCTTGTGAT
P. subrhomboidea	ATATTACACA	GAATTGATGT	GGGCATTGTG	ATTGATCTGG	AGCCAGCTCG	TGCTTGTGAT
P. subrhomboidea var. tribract	ATATTACACA	GAATTGATGT	GGGCATTGTG	ATTGATCTGG	AGCCAGCTCG	TGCTTGTGAT
P. subulata	ATATTACACA	GAATTGATGT	GGGCATTGTG	ATTGATCTGG	AGCCAGCTCG	TGCTTGCGAT
P. swinglei	ATATTACACA	GAATTGATGT	GGGCATTGTG	ATTGATCTGG	AGCCAGCTCG	TGCTTGTGAT
P. tabacum	ATATTACACA	GAATTGACGT	GGGCATTGTG	GTTGACCTGG	AGCCAGCTCG	TGCTTGTGAT
P. tenuifolia	ATATTACACA	GAATTGATGT	GGGCATTGTG	ATTGATCTGG	AGCCAGCTCG	TGCTTGTGAT
P. tenuituba	ATATTGCACA	GAATTGACGT	GGGCATTGTG	GTTGATCTGG	AGCCAGCTCG	TGCTTGTGAT
P. tiandengensis	ATATTACACA	GAATTGACGT	GGGCATTGTG	ATTGATCTGG	AGCCAGCTCG	TGCTTGTGAT
P. tribracteata	ATATTACACA	GAATTGACGT	GGGCATTGTG	ATTGATCTGG	AGCCAGCTCG	TGCTTGTGAT
P. villosissima	ATATTACACA	GAATTGATGT	GGGCATTGTG	ATTGATCTGG	AGCCAGCTCG	TGCTTGTGAT
P. wentsaii	ATATTACACA	GAATTGATGT	GGGCATTGTG	ATTGATCTGG	AGCCAGCTCG	TGCTTGTGAT
P. xiuningensis	ATATTACACA	GAATTGACGT	GGGCATTGTG	GTTGATCTGG	AGCCAGCTCG	TGCTTGTGAT
P. xizii	ATATTACACA	GAATTGACGT	GGGCATTGTG	GTTGATCTGG	AGCCAGCTCG	TGCTTGTGAT
P. yangchunensis	ATATTACACA	GAATTGATGT	GGGCATTGTG	ATTGATCTGG	AGCCAGCTCG	TGCTTGTGAT
P. yongxingensis	ATATTGCACA	GAATTGACGT	GGGCATTGTG	GTTGATCTGG	AGCCAGCTCG	TGCTTGTGAT
P. yungfuensis	ATATTACACA	GAATTGATGT	GGGCATTGTG	ATTGATCTGG	AGCCAGCTCG	TGCTTGTGAT
P. cordifolia	ATATTACACA	GAATTGACGT	GGGCATTGTG	ATTGATCTGG	AGCCAGCTCG	TGCTTGTGAT
P. danxiaensis	ATATTACACA	GAATTGACGT	GGGCATTGTG	GTTGATCTAG	AGCCAGCTCG	TGCTTGTGAT
P. leiophylla & P. napoensis	ATATTACACA	GAATTGACGT	GGGCATTGTG	ATTGATCTGG	AGCCAGCTCG	TGCTTGTGAT
P. verecunda	ATATTACACA	GAATTGATGT	GGGCATTGTG	ATTGATCTGG	AGCCAGCTCG	TGCTTGCGAT
sp. nov. 6	ATATTACACA	GAATTGATGT	GGGCATTGTG	ATTGATCTGG	AGCCAGCTCG	TGCTTGTGAT
sp. nov. 8	ATATTACACA	GAATTGACGT	GGGCATTGTG	GTTGATCTGG	AGCCAGCTCG	TGCTTGTGAT
sp. nov. 9	ATATTACACA	GAATTGACGT	GGGCATTGTG	GTTGATCTGG	AGCCAGCTCG	TGCTTGTGAT
sp. nov. 10	ATATTACACA	GAATTGACGT	GGGCATTGTG	GTTGATCTGG	AGCCAGCTCG	TGCTTGTGAT
sp. nov. 12	ATATTACACA	GAATTGACGT	GGGCATTGTG	GTTGATCTGG	AGCCAGCTCG	TGCTTGTGAT
sp. nov. 13	ATATTACACA	GAATTGACGT	GGGCATTGTG	GTTGATCTAG	AGCCAGCTCG	TGCTTGTGAT
sp. nov. 16	ATATTACACA	GAATTGACGT	GGGCATTGTG	GTTGATCTGG	AGCCAGCTCG	TGCTTGTGAT
sp. nov. 18	ATATTACACA	GAATTGACGT	GGGCATTGTG	ATTGATCTGG	AGCCAGCTCG	TGCTTGCGAT
sp. nov. 19	ATATTACACC	GAATTGATGT	GGGCATTGTG	ATTGATCTGG	AGCCAGCTCG	TGCTTGTGAT
Didymocarpus hancei	ATATTACACA	GAATTGATGT	GGGCATTGTG	ATTGATCTGG	AGCCAGCTCG	TGCTGGTGAT
Petrocodon dealbatus	ATATTACACA	GAATTGATGT	GGGCATTGTG	ATTGATCTGG	AGCCAGCTCG	TGCTGGTGAT

	490	500	510	520	530	540
P. bicolor	CCTGCTATGT	TGCATGCTAG	TGCTGTGCAA	TCGCAGAAAC	TAGCTGTGAG	AGCCATATCT
P. bipinnatifida	CCTGCGATGT	TGCATGCTAG	TGCTGTGCAA	TCGCAGAAAC	TAGCTGTGAG	AGCCATATCT
P. chizhouensis	CCTGCTATGT	TGCATGCTAG	TGCTGTGCAA	TCGCAGAAAC	TAGCTGTGAG	AGCCATATCT
P. cordata	CCTGCTATGT	TGCATGCTAG	TGCTGTGCAA	TCGCAGAAAC	TAGCTGTGAG	AGCCATATCT
P. depressa	CCTGGTATGT	TGCATGCTAG	TGCTGTGCAA	TCGCAGAAAT	TAGCTGTGAG	AGCCATATCT
P. dongguanica	CCTGCTATGT	TGCATGCTAG	TGCTGTGCAA	TCGCAGAAAC	TAGCTGTGAG	AGCCATATCT
P. eburnea	CCTGCTATGT	TGCATGCTAG	TGCTGTGCAA	TCGCAGAAAC	TAGCTGTGAG	AGCCATATCT
P. fimbrisepala	CCTGCTATGT	TGCATGCTAG	TGCTGTGCAA	TCGCAGAAAC	TAGCTGTGAG	AGCCATATCT
P. glandulosa var. yangshuoens	CCTGCTATGT	TGCATGCTAG	TGCTGTGCAA	TCGCAGAAAC	TAGCTGTGAG	AGCCATATCT
P. gueilinensis var. brachycar	CCTGCTATGT	TGCATGCTAG	TGCTGTGCAA	TCGCAGAAAC	TAGCTGTGAG	AGCCATATCT
P.guihaiensis	CCTGCTATGT	TGCATGCTAG	TGCTGTGCAA	TCGCAGAAAC	TAGCTGTGAG	AGCCATATCT
P. heterotricha & P. pterppoda	CCTGCTATGT	TGCATGCTAG	TGCTGTGCAA	TCGCAGAAAC	TAGCCGTGAG	AGCCATATCT
P. hochiensis	CCTGCTATGT	TGCATGCTAG	TGCTGTGCAA	TCGCAGAAAC	TAGCTGTGAG	AGCCATATCT
P. huaijiensis	CCTGCTATGT	TGCATGCTAG	TGCTGTGCAA	TCGCAGAAAC	TAGCTGTGAG	AGCCATATCT
P. langshanica	CCTGCTATGT	TGCATGCTAG	TGCTGTGCAA	TCGCAGAAAC	TAGCTGTGAG	AGCCATATCT
P. latinervis	CCTGCTATGT	TGCATGCTAG	TGCTGTGCAA	TCGCAGAAAC	TAGCTGTGAG	AGCCATATCT
P. laxiflora	CCCGCTATGT	TGCATGCTAG	TGCTGTGCAA	TCGCAGAAAC	TAGCTGTGAG	AGCCATATCT
P. leprosa	CCTGCTATGT	TGCATGCTAG	TGCTGTGCAA	TCGCAGAAAC	TAGCTGTGAG	AGCCATATCT
P. liguliformis	CCTGCTATGT	TGCATGCTAG	TGCTGTGCAA	TCGCAGAAAC	TAGCTGTGAG	AGCCATATCT
P. lijiangensis	CCTGCTATGT	TGCATGCTAG	TGCTGTGCAA	TCGCAGAAAC	TAGCTGTGAG	AGCCATATCT
P. linearifolia	CCTTCTATGT	TGCATGCTAG	TGCTGTGCAA	TCGCAGAAAC	TAGCTGTGAG	AGCCATATCT
P. lobulata	CCTGCTATGT	TGCATGCTAG	TGCTGTGCAA	TCGCAGAAAC	TAGCTGTGAG	AGCCATATCT
P. longii	CCTGCTATGT	TGCATGCTAG	TGCTGTGCAA	TCGCAGAAAC	TAGCTGTGAG	AGCCATATCT
P. lunglinensis	CCTGCTATGT	TGCATGCTAG	TGCTGTGCAA	TCGCAGAAAC	TAGCTGTGAG	AGCCATATCT
P. lungzhouensis	CCTGCTATGT	TGCATGCTAG	TGCTGTGCAA	TCGCAGAAAC	TAGCTGTGAG	AGCCATATCT
P. luochengensis	CCTGCTATGT	TGCATGCTAG	TGCTGTGCAA	TCGCAGAAAC	TAGCTGTGAG	AGCCATATCT
P. lutea	CCTGCTATGT	TGCATGCTAG	TGCTGTGCAA	TCGCAGAAAC	TAGCTGTGAG	AGCCATATCT
P. mabaensis	CCTGCTATGT	TGCATGCTAG	TGCTGTGCAA	TCGCAGAAAT	TAGCTGTGAG	AGCCATATCT
P. macrodonta	CCTGCTATGT	TGCATGCTAG	TGCTGTGCAA	TCGCAGAAAC	TAGCTGTGAG	AGCCATATCT
P. medica	CCTGCTATGT	TGCATGCTAG	TGCTGTGCAA	TCGCAGAAAC	TAGCTGTGAG	AGCCATATCT
P. moii	CCTGCTATGT	TGCATGCTAG	TGCTGTGCAA	TCGCAGAAAC	TAGCTGTGAG	AGCCATATCT
P. mollifolia	CCTGCTATGT	TGCATGCTAG	TGCTGTGCAA	TCGCAGAAAC	TAGCTGTGAG	AGCCATATCT
P. obtusidentata	CCTGCTATGT	TGCATGCTAG	TGCTGTGCAA	TCGCAGAAAC	TAGCTGTGAG	AGCCATATCT
P. orthandra	CCTGCTATGT	TGCATGCTAG	TGCTGTGCAA	TCGCAGAAAC	TAGCTGTGAG	AGCCATATCT
P. parvifolia	CCTGCTATGT	TGCATGCTAG	TGCTGTGCAA	TCGCAGAAAC	TAGCTGTGAG	AGCCATATCT
P. pinnatifida	CCTGCTATGT	TGCATGCTAG	TGCTGTACAA	TCGCAGAAAC	TAGCTGTGAG	AGCCATATCT
P. pulchurfolia	CCTTCTATGT	TGCATGCTAG	TGCTGTGCAA	TCGCAGAAAC	TAGCTGTGAG	AGCCATATCT
P. renifolia	CCTGCTATGT	TGCATTCTAG	TGCTGTGCAA	TCGCAGAAAC	TAGCTGTGAG	AGCCATATCT
P. repanda	CCTGCGATGT	TGCATGCTAG	TGCTGTGCAA	TCGCAGAAAC	TAGCTGTGAG	AGCCATATCT
P. ronganensis	CCTGCTATGT	TGCATGCTAG	TGCTGTGCAA	TCGCAGAAAC	TAGCTGTGAG	AGCCATATCT
P. sclerophylla	CCTGCTATGT	TGCATGCTAG	TGCTGTGCAA	TCGCAGAAAC	TAGCTGTGAG	AGCCATATCT
P. sinensis	CCTGCTATGT	TGCATGCTAG	TGCTGTACAA	TCGCAGAAAC	TAGCTGTGAG	AGCCATATCT
P. spinulosa	CCTGCTATGT	TGCATGCTAG	TGCTGTGCAA	TCGCAGAAAC	TAGCTGTGAG	AGCCATATCT
P. subrhomboidea	CCTGCTATGT	TGCATGCTAG	TGCTGTGCAA	TCGCAGAAAC	TAGCTGTGAG	AGCCATATCT
P. subrhomboidea var. tribract	CCTGCTATGT	TGCATGCTAG	TGCTGTGCAA	TCGCAGAAAC	TAGCTGTGAG	AGCCATATCT
P. subulata	CCTGCTATGT	TGCATGCTAG	TGCTGTGCAA	TCGCAGAAAC	TAGCTGTGAG	AGCCATATCT
P. swinglei	CCTTCTATGT	TGCATGCTAG	TGCTGTGCAA	TCGCAGAAAC	TAGCTGTGAG	AGCCATATCT
P. tabacum	CCTGCTATGT	TGCATGCTAG	TGCTGTGCAA	TCGCAGAAAC	TAGCTGTGAG	AGCCATATCT
P. tenuifolia	CCTGCTATGT	TGCATGCTAG	TGCTGTGCAA	TCGCAGAAAC	TAGCTGTGAG	AGCCATATCT
P. tenuituba	CCTGCTATGT	TGCATGCTAG	TGCTGTGCAA	TCGCAGAAAC	TAGCTGTGAG	AGCCATATCT
P. tiandengensis	CCTGCTATGT	TGCATGCTAG	TGCTGTGCAA	TCGCAGAAAC	TAGCTGTGAG	AGCCATATCT
P. tribracteata	CCTGCTATGT	TGCATGCCAG	TGCTGTGCAA	TCGCAGAAAC	TAGCTGTGAG	AGCCATATCT
P. villosissima	CCTGCTATGT	TGCATGCTAG	TGCTGTGCAA	TCGCAGAAAC	TAGCTGTGAG	AGCCATATCT
P. wentsaii	CCTGCTATGT	TGCATGCTAG	TGCTGTGCAA	TCGCAGAAAC	TAGCTGTGAG	AGCCATATCT
P. xiuningensis	CCTGCTATGT	TGCATGCTAG	TGCTGTGCAA	TCGCAGAAAC	TAGCTGTGAG	AGCCATATCT
P. xizii	CCTGCTATGT	TGCATGCTAG	TGCTGTGCAA	TCGCAGAAAC	TAGCTGTGAG	AGCCATATCT
P. yangchunensis	CCTGCTATGT	TGCATGCTAG	TGCTGTGCAA	TCGCAGAAAC	TAGCTGTGAG	AGCCATATCT
P. yongxingensis	CCTGCTATGT	TGCATGCTAG	TGCTGTGCAA	TCGCAGAAAC	TAGCTGTGAG	AGCCATATCT
P. yungfuensis	CCTGCTATGT	TGCATGCTAG	TGCTGTACAA	TCGCAGAAAC	TAGCTGTGAG	AGCCATATCT
P. cordifolia	CCTGCTATGT	TGCATGCTAG	TGCTGTGCAA	TCGCAGAAAC	TAGCTGTGAG	AGCCATATCT
P. danxiaensis	CCTGCTATGT	TGCATGCTAG	TGCTGTGCAA	TCGCAGAAAT	TAGCTGTGAG	AGCCATATCT
P. leiophylla & P. napoensis	CCTGCTATGT	TGCATGCTAG	TGCTGTGCAA	TCGCAGAAAC	TAGCTGTGAG	AGCCATATCT
P. verecunda	CCTGCTATGT	TGCATGCTAG	TGCTGTGCAA	TCGCAGAAGC	TAGCTGTGAG	AGCCATATCT
sp. nov. 6	CCTGCTATGT	TGCATGCTAG	TGCTGTGCAA	TCGCAGAAAC	TAGCTGTGAG	AGCCATATCT
sp. nov. 8	CCTGCTATGT	TGCATGCTAG	TGCTGTGCAA	TCGCAGAAAC	TAGCTGTGAG	AGCCATATCT
sp. nov. 9	CCTGCTATGT	TGCATGCTAG	TGCTGTGCAA	TCGCAGAAAC	TAGCTGTGAG	AGCCATATCT
sp. nov. 10	CCTGCTATGT	TGCATGCTAG	TGCTGTGCAA	TCGCAGAAAC	TAGCTGTGAG	AGCCATATCT
sp. nov. 12	CCTGCTATGT	TGCATGCTAG	TGCTGTGCAA	TCGCAGAAAC	TAGCTGTGAG	AGCCATATCT
sp. nov. 13	CCTGCTATGT	TGCATGCTAG	TGCTGTGCAA	TCGCAGAAAT	TAGCTGTGAG	AGCCATATCT
sp. nov. 16	CCTGCTATGT	TGCATGCTAG	TGCTGTGCAA	TCGCAGAAAC	TAGCTGTGAG	AGCCATATCT
sp. nov. 18	CCTGCTATGT	TGCATGCTAG	TGCTGTGCAA	TCGCAGAAAC	TAGCTGTGAG	AGCCATATCT
sp. nov. 19	CCTGCTATGT	TGCATGCTAG	TGCTGTGCAA	TCGCAGAAAC	TAGCTGTGAG	AGCCATATCT
Didymocarpus hancei	CCTGCTATGT	TGCATGCTAG	TGCTGTGCAA	TCGCAGAAAC	TAGCTGTGAG	AGCCATATCT
Petrocodon dealbatus	CCTGCTATGT	TGCATGCTAG	TGCTGTGCAA	TCGCAGAAAC	TAGCTGTGAG	AGCCATATCT

	541–550	551–560	561–570	571–580	581–590	591–600
	550	560	570	580	590	600
P. bicolor	AGGCTGCAGT	CTCTTCCTGG	GGGCGATGTA	GGAGTTTTGT	GTGATACAGT	CGTAGAAGAT
P. bipinnatifida	AGGCTGCAGT	CTCTTCCTGG	GGGCGATGTA	GGAGTTTTGT	GTGATACAGT	CGTAGAAGAT
P. chizhouensis	AGGCTGCAGT	CTCTTCCTGG	GGGCGATGTA	GGAGTTTTGT	GTGATACAGT	CGTAGAAGAT
P. cordata	AGGCTGCAGT	CTCTTCCTGG	GGGCGATGTT	GGAGTTTTGT	GTGATACAGT	CGTAGAAGAT
P. depressa	AGGCTGCAGT	CTCTTCCTGG	GGGCGATGTA	GGAGTTTTGT	GTGACACAGT	CGTAGAAGAT
P. dongguanica	AGGCTGCAGT	CTCTTCCTGG	GGGCGATGTA	GGAGTTTTGT	GTGATACAGT	CGTTGAAGAT
P. eburnea	AGGCTGCAGT	CTCTTCCTGG	GGGCGATGTA	GGAGTTTTGT	GTGATACAGT	CGTAGAAGAT
P. fimbrisepala	AGGCTGCAGT	CTCTTCCTGG	GGGCGATGTT	GGAGTTTTGT	GTGATACAGT	CGTAGAAGAT
P. glandulosa var. yangshuoens	AGGCTGCAGT	CTCTTCCTGG	GGGCGATGTA	GGAGTTTTGT	GTGATACAGT	CGTAGAAGAT
P. gueilinensis var. brachycar	AGGCTGCAGT	CTCTTCCTGG	GGGCGATGTA	GGAGTTTTGT	GTGATACAGT	CGTAGAAGAT
P.guihaiensis	AGGCTGCAGT	CTCTTCCTGG	GGGCGATGTT	GGAGTTTTGT	GTGATACAGT	CGTAGAAGAT
P. heterotricha & P. pterppoda	AGGCTGCAGT	CTCTTCCTGG	GGGCGATGTA	GGAGTTTTGT	GTGATACAGT	CGTAGAAGAT
P. hochiensis	AGGCTGCAGT	CTCTTCCTGG	GGGTGATGTT	GGAGTTTTGT	GTGATACAGT	CGTAGAAGAT
P. huaijiensis	AGGCTGCAGG	CTCTTCCTGG	GGGCGATGTA	GGAGTTTTGT	GTGATACAGT	CGTAGAAGAT
P. langshanica	AGGCTGCAGT	CTCTTCCTGG	GGGCGATGTA	GGAGTTTTGT	GTGATACAGT	CGTAGAAGAT
P. latinervis	AGGCTGCAGT	CTCTTCCTGG	GGGCGATGTA	GGAGTTTTGT	GTGATACAGT	TGTAGAAGAT
P. laxiflora	AGGCTGCAGT	CTCTCCCTGG	GGGCGATGTA	GGAGTTTTGT	GTGATACAGT	CGTAGAAGAT
P. leprosa	AGGCTGCAGT	CTCTTCCTGG	GGGCGATGTA	GGAGTTTTGT	GTGATACAGT	CGTAGAAGAT
P. liguliformis	AGGCTGCAGT	CTCTTCCTGG	GGGCGATGTA	GGAGTTTTGT	GTGATACAGT	CGTAGAAGAT
P. lijiangensis	AGGCTGCAGT	CTCTTCCTGG	GGGCGATGTT	GGAGTTTTGT	GTGATACAGT	CGTAGAAGAT
P. linearifolia	AGGCTGCAGT	CTCTTCCTGG	GGGCGATGTA	GGAGTTTTGT	GTGATACAGT	CGTAGAAGAT
P. lobulata	AGGCTGCAGT	CTCTTCCTGG	GGGCGATGTA	GGAGTTTTGT	GTGATACAGT	CGTAGAAGAT
P. longii	AGGCTGCAGT	CTCTTCCTGG	GGGCGATGTT	GGAGTTTTGT	GTGATACAGT	CGTAGAAGAT
P. lunglinensis	AGGCTGCAGT	CTCTTCCTGG	GGGCGATGTA	GGAGTTTTGT	GTGATACAGT	CGTAGAAGAT
P. lungzhouensis	AGGCTGCAGT	CTCTTCCTGG	GGGCGATGTA	GGAGTTTTGT	GTGATACAGT	CGTAGAAGAT
P. luochengensis	AGGCTGCAGT	CTCTTCCTGG	GGGCGATGTA	GGAGTTTTGT	GTGATACAGT	CGTAGAAGAT
P. lutea	AGGCTGCAGT	CTCTTCCTGG	GGGCGATGTA	GGAGTTTTGT	GTGATACAGT	CGTAGAAGAT
P. mabaensis	AGGCTGCAGT	CTCTTCCTGG	GGGCGATGTA	GGAGTTTTGT	GTGACACAGT	CGTAGAAGAT
P. macrodonta	AGGCTGCAGT	CTCTTCCTGG	GGGCGATGTA	GGAGTTTTGT	GTGATACAGT	CGTAGAAGAT
P. medica	AGGCTGCAGT	CTCTTCCTGG	GGGCGATGTA	GGAGTTTTGT	GCGATACAGT	CGTAGAAGAT
P. moii	AGGCTGCAGT	CTCTTCCTGG	GGGCGATGTA	GGAGTTTTGT	GTGATACAGT	CGTAGAAGAT
P. mollifolia	AGGCTGCAGT	CTCTTCCTGG	GGGCGATGTA	GGAGTTTTGT	GTGATACAGT	CGTAGAAGAT
P. obtusidentata	AGGCTGCAGT	CTCTTCCTGG	GGGCGACGTA	GGAGTTTTGT	GTGATACAGT	CGTAGAAGAT
P. orthandra	AGGCTGCAGT	CTCTTCCTGG	GGGCGATGTA	GGAGTTTTGT	GTGATACAGT	CGTAGAAGAT
P. parvifolia	AGGCTGCAGT	CTCTTCCTGG	GGGCGATGTT	GGAGTTTTGT	GTGATACAGT	CGTAGAAGAT
P. pinnatifida	AGGCTGCAGT	CTCTTCCTGG	GGGCGATGTA	GGAGTTTTGT	GTGATACAGT	CGTAGAAGAT
P. pulchurfolia	AGGCTGCAGT	CTCTTCCTGG	GGGCGATGTA	GGAGTTTTGT	GTGATACAGT	CGTAGAAGAT
P. renifolia	AGGCTGCAGT	CTCTTCCTGG	GGGCGATGTT	GGAGTTTTGT	GTGATACAGT	CGTAGAAGAT
P. repanda	AGGCTGCAGT	CTCTTCCTGG	GGGCGATGTT	GGAGTTTTGT	GTGATACAGT	CGTAGAAGAT
P. ronganensis	AGGCTGCAGT	CTCTTCCTGG	GGGCGATGTA	GGAGTTTTGT	GTGATACAGT	CGTAGAAGAT
P. sclerophylla	AGGCTGCAGT	CTCTTCCTGG	GGGCGATGTA	GGAGTTCTGT	GTGATACAGT	CGTAGAAGAT
P. sinensis	AGGCTGCAGT	CTCTTCCTGG	GGGCGATGTA	GGAGTTTTGT	GTGATACAGT	CGTAGAAGAT
P. spinulosa	AGGCTGCAGT	CTCTTCCTGG	GGGCGATGTA	GGAGTTTTGT	GTGACACAGT	CGTAGAAGAT
P. subrhomboidea	AGGCTGCAGT	CTCTTCCTGG	GGGCGATGTT	GGAGTTTTGT	GTGATACAGT	CGTAGAAGAT
P. subrhomboidea var. tribract	AGGCTGCAGT	CTCTTCCTGG	GGGCGATGTA	GGAGTTTTGT	GTGATACAGT	CGTAGAAGAT
P. subulata	AGGCTGCAGT	CTCTTCCTGG	GGGCGATGTA	GGAGTTTTGT	GTGATACAGT	CGTAGAAGAT
P. swinglei	AGGCTGCAGT	CTCTTCCTGG	GGGCGATGTA	GGAGTTTTGT	GTGATACAGT	CGTAGAAGAT
P. tabacum	AGGCTGCAGT	CTCTTCCTGG	GGGCGATGTA	GGAGTTTTGT	GTGATACAGT	CGTAGAAGAT
P. tenuifolia	AGGCTGCAGT	CTCTTCCTGG	GGGCGATGTT	GGAGTTCTGT	GTGATACAGT	CGTAGAAGAT
P. tenuituba	AGGCTGCAGT	CTCTTCCTGG	GGGCGATGTA	GGAGTTTTGT	GTGATACAGT	CGTAGAAGAT
P. tiandengensis	AGGCTGCAGT	CTCTTCCTGG	GGGCGATGTA	GGAGTTTTGT	GTGATACAGT	CGTAGAAGAT
P. tribracteata	AGGCTGCAGT	CTCTTCCTGG	GGGCGATATA	GGAGTTTTGT	GTGATACAGT	CGTAGAAGAT
P. villosissima	AGGCTGCAGT	CTCTTCCTGG	GGGCGATGTA	GGAGTTTTGT	GTGATACAGT	CGTAGAAGAT
P. wentsaii	AAGCTGCAGT	CTCTTCCTGG	GGGCGATGTA	GGAGTTTTGT	GTGATACAGT	CGTAGAAGAT
P. xiuningensis	AGGCTGCAGT	CTCTTCCTGG	GGGCGATGTA	GGAATTTTGT	GTGATACAGT	CGTAGAAGAT
P. xizii	AGGCTGCAGT	CTCTTCCTGG	GGGCGATGTA	GGAGTTTTGT	GTGATACAGT	CGTAGAAGAT
P. yangchunensis	AGGCTGCAGT	CTCTTCCTGG	GGGCGATGTA	GGAGTTTTGT	GTGATACAGT	CGTAGAAGAT
P. yongxingensis	AGGCTGCAGT	CTCTTCCTGG	GGGCGATGTA	GGAGTTTTGT	GTGATACAGT	CGTAGAAGAT
P. yungfuensis	AGGCTGCAGT	CTCTTCCTGG	GGGCGATGTA	GGAGTTTTGT	GTGATACAGT	CGTAGAAGAT
P. cordifolia	AGGCTGCAGT	CTCTTCCTGG	GGGCGATGTT	GGAGTTTTGT	GTGATACAGT	CGTAGAAGAT
P. danxiaensis	AGGCTGCAGT	CTCTTCCTGG	GGGCGATGTA	GGAGTTTTGT	GTGACACAGT	CGTAGAAGAT
P. leiophylla & P. napoensis	AGGCTGCAGT	CTCTTCCTGG	GGGCGATGTA	GGAGTTTTGT	GTGATACAGT	CGTAGAAGAT
P. verecunda	AGGCTGCAGT	CTCTTCCTGG	GGGCGATGTA	GGAGTTTTGT	GTGATACAGT	CGTAGAAGAT
sp. nov. 6	AGGCTGCAGT	CTCTTCCTGG	GGGAGATGTA	GGAGTTTTGT	GTGATACAGT	CGTAGAAGAT
sp. nov. 8	AGGCTGCAGT	CTCTTCCTGG	GGGCGATGTA	GGAGTTTTGT	GTGATACAGT	CGTAGAAGAT
sp. nov. 9	AGGCTGCAGT	CTCTTCCTGG	GGGCGATGTA	GGAGTTTTGT	GTGATACAGT	CGTAGAAGAT
sp. nov. 10	AGGCTGCAGT	CTCTTCCTGG	GGGCGATGTA	GGAGTTTTGT	GTGATACAGT	CGTAGAAGAT
sp. nov. 12	AGGCTGCAGT	CTCTTCCTGG	GGGCGATGTA	GGAGTTTTGT	GTGATACTGT	CGTAGAAGAT
sp. nov. 13	AGGCTGCAGT	CTCTTCCTGG	GGGCGATGTA	GGAGTTTTGT	GTGACACAGT	CGTAGAAGAT
sp. nov. 16	AGGCTGCAGT	CTCTTCCTGG	GGGCGATGTA	GGAGTTTTGT	GTGATACCGT	CGTAGAAGAT
sp. nov. 18	AGGCTGCAGT	CTCTTCCTGG	GGGCGATGTA	GGAGTTTTGT	GTGATACAGT	CGTAGAAGAT
sp. nov. 19	AGGCTGCAGT	CTCTTCCTGG	GGGCGATGTA	GGAGTTTTGT	GTGATACAGT	CGTAGAAGAT
Didymocarpus hancei	AGGCTGCAGT	CTCTTCCAGG	GGGCGATGTA	GGAGTTTTGT	GTGACACAGT	CGTAGAAGAT
Petrocodon dealbatus	AGGCTGCAGT	CTCTTCCAGG	GGGCGATGTA	GGAGTTTTGT	GTGATACAGT	CGTAGAAGAT

Taxon	610	620	630	640	650	660
P. bicolor	GTCCAAAAAC	TTACTGGTTA	TGATAGGGTT	ATGGTGTATA	AGTTCCATGA	AGATAATCAT
P. bipinnatifida	GTCCAAAAAC	TTACTGGTTA	TGATAGGGTT	ATGGTGTATA	AGTTCCATGA	AGATAATCAT
P. chizhouensis	GTCCAAAAAC	TTACTGGTTA	TGATAGGGTT	ATGGTGTATA	AGTTCCATGA	AGATAATCAT
P. cordata	GTCCAAAAAC	TAACTGGTTA	TGATAGGGTT	ATGGTGTATA	AGTTCCATGA	AGATAATCAT
P. depressa	GTCCAAAAAC	TTACTGGTTA	TGATAGGGTT	ATGGTGTATA	AGTTCCATGA	AGATAATCAT
P. dongguanica	GTCCAAAAAC	TAACTGGTTA	TGATAGGGTT	ATGGTGTATA	AGTTCCATGA	AGATAATCAT
P. eburnea	GTCCAAAAAC	TTACTGGTTA	TGATAGGGTT	ATGGTGTATA	AGTTCCATGA	AGATAATCAT
P. fimbrisepala	GTCCAAAAAC	TAACTGGTTA	TGATAGGGTT	ATGGTGTATA	AGTTCCATGA	AGATAATCAT
P. glandulosa var. yangshuoens	GTCCAAAAAC	TAACTGGTTA	TGATAGGGTT	ATGGTGTATA	AGTTCCATGA	AGATAATCAT
P. gueilinensis var. brachycar	GTCCAAAAAC	TTACTGGTTA	TGATAGGGTT	ATGGTGTATA	AGTTCCATGA	AGATAATCAT
P.guihaiensis	GTCCAAAAAC	TAACTGGTTA	TGATAGGGTT	ATGGTGTATA	AGTTCCATGA	AGATAATCAT
P. heterotricha & P. pterppoda	ATCCAAAAAC	TTACTGGTTA	TGATAGGGTT	ATGGTGTATA	AGTTCCATGA	AGATAATCAT
P. hochiensis	GTCCAAAAAC	TAACTGGCTA	TGATAGGGTT	ATGGTATATA	AGTTCCATGA	AGATAATCAT
P. huaijiensis	GTCCAAAAAC	TTACTGGTTA	TGATAGGGTT	ATGGTGTATA	AGTTCCATGA	AGATAATCAT
P. langshanica	GTCCAAAAAC	TTACTGGTTA	TGATAGGGTT	ATGGTGTATA	AGTTTCATGA	AGATAATCAT
P. latinervis	GTCCAAAAAC	TTACTGGTTA	TGATAGGGTT	ATGGTGTATA	AGTTCCATGA	AGATAATCAT
P. laxiflora	GTCCAAAAAC	TTACTGGTTA	TGACAGGGTT	ATGGTGTATA	AGTTCCATGA	AGATAATCAT
P. leprosa	GTCCAAAAAC	TTACTGGTTA	TGATAGGGTT	ATGGTGTATA	AGTTCCATGA	AGATAATCAT
P. liguliformis	GTCCAAAAAC	TTACTGGTTA	TGATAGGGTT	ATGGTGTATA	AGTTCCATGA	AGATAATCAT
P. lijiangensis	GTCCAAAAAC	TAACTGGTTA	TGATAGGGTT	ATGGTGTATA	AGTTCCATGA	AGATAATCAT
P. linearifolia	GTCCAAAAAC	TTACTGGTTA	TGATAGGGTT	ATGGTGTATA	AGTTCCACGA	AGATAATCAT
P. lobulata	GTCCAAAAAC	TTACTGGTTA	TGATAGGGTT	ATGGTGTATA	AGTTCCATGA	AGATAATCAT
P. longii	GTCCAAAAAC	TAACTGGTTA	TGATAGGGTT	ATGGTGTATA	AGTTCCATGA	AGATAATCAT
P. lunglinensis	GTCCAAAAAC	TTACTGGTTA	TGATAGGGTT	ATGGTGTATA	AGTTCCATGA	AGATAATCAT
P. lungzhouensis	GTCCAAAAAC	TAACTGGTTA	TGATAGGGTT	ATGGTGTATA	AGTTCCATGA	AGATAATCAT
P. luochengensis	GTCCAAAAAC	TTACTGGTTA	TGATAGGGTT	ATGGTGTATA	AGTTCCACGA	AGATAATCAT
P. lutea	GTCCAAAAAC	TTACTGGTTA	TGATAGGGTT	ATGGTGTATA	AGTTCCATGA	AGATAATCAT
P. mabaensis	GTCCAAAAAC	TTACTGGTTA	TGATAGGGTT	ATGGTGTATA	AGTTCCATGA	AGATAATCAT
P. macrodonta	GTCCAAAAAC	TTACTGGTTA	TGATAGGGTT	ATGGTGTATA	AGTTCCATGA	AGATAATCAT
P. medica	GTCCAAAAAC	TAACTGGTTA	TGATAGGGTT	ATGGTGTATA	AGTTCCATGA	AGATAATCAT
P. moii	GTCCAAAAAC	TTACTGGTTA	TGATAGGGTT	ATGGTGTATA	AGTTCCATGA	AGATAATCAT
P. mollifolia	GTCCAAAAAC	TTACTGGTTA	TGATAGGGTT	ATGGTGTATA	AGTTCCATGA	AGATAATCAT
P. obtusidentata	GTCCAAAAAC	TCACTGGTTA	TGATAGGGTT	ATGGTGTATA	AGTTCCATGA	AGATAATCAT
P. orthandra	GTCCAAAAAC	TTACTGGTTA	TGATAGGGTT	ATGGTGTATA	AGTTCCATGA	AGATAATCAT
P. parvifolia	GTCCAAAAAC	TTACTGGTTA	TGATAGGGTT	ATGGTGTATA	AGTTCCATGA	AGATAATCAT
P. pinnatifida	GTCCAAAAAC	TTACTGGTTA	TGATAGGGTT	ATGGTGTATA	AGTTCCATGA	AGACAATCAT
P. pulchurfolia	GTCCAAAAAC	TTACTGGTTA	TGATAGGGTT	ATGGTGTATA	AGTTCCACGA	AGATAATCAT
P. renifolia	GTCCAAAAAC	TTACTGGTTA	TGATAGGGTT	ATGGTGTATA	AGTTCCATGA	AGATAATCAT
P. repanda	GTCCAAAAAC	TTACTGGTTA	TGATAGGGTT	ATGGTGTATA	AGTTCCATGA	GGATAATCAT
P. ronganensis	GTCCAAAAAC	TTACTGGTTA	TGATAGGGTT	ATGGTGTATA	AGTTCCATGA	AGATAATCAT
P. sclerophylla	GTCCAAAAAC	TTACTGGTTA	TGATAGGGTT	ATGGTGTATA	AGTTCCATGA	AGATAATCAT
P. sinensis	GTCCAAAAAC	TTACTGGTTA	TGATAGGGTT	ATGGTGTATA	AGTTCCATGA	AGATAATCAT
P. spinulosa	GTCCAAAAAC	TTACTGGTTA	TGATAGGGTT	ATGGTGTATA	AGTTCCATGA	AGATAATCAT
P. subrhomboidea	GTCCAAAAAC	TAACTGGTTA	TGATAGGGTT	ATGGTGTATA	AGTTCCATGA	AGATAATCAT
P. subrhomboidea var. tribract	GTCCAAAAAC	TTACTGGTTA	TGATAGGGTT	ATGGTGTATA	AGTTCCACGA	AGATAATCAT
P. subulata	GTCCAAAAAC	TAACTGGTTA	TGATAGGGTT	ATGGTGTATA	AGTTCCATGA	AGATAATCAT
P. swinglei	GTCCAAAAAC	TTACTGGTTA	TGACAGGGTT	ATGGTGTATA	AGTTCCATGA	AGATAATCAT
P. tabacum	GTCCAAAAAC	TTACTGGTTA	TGATAGGGTT	ATGGTGTATA	AGTTCCATGA	AGATAATCAT
P. tenuifolia	GTCCAAAAAC	TAACTGGTTA	TGATAGGGTT	ATGGTGTATA	AGTTCCATGA	AGATAATCAT
P. tenuituba	GTCCAAAAAC	TTACTGGTTA	TGATAGGGTT	ATGGTGTATA	AGTTCCATGA	AGATAATCAT
P. tiandengensis	GTCCAAAAAC	TTACTGGTTA	TGATAGGGTT	ATGGTGTATA	AGTTCCATGA	AGATAATCAT
P. tribracteata	GTCCAAAAAC	TTACTGGTTA	TGATAGGGTT	ATGGTGTATA	AGTTCCATGA	AGATAATCAT
P. villosissima	GTCCAAAAAC	TTACTGGTTA	TGATAGGGTT	ATGGTGTATA	AGTTCCATGA	AGATAATCAT
P. wentsaii	GTCCAAAAAC	TTACTGGTTA	TGATAGGGTT	ATGGTGTATA	AGTTCCATGA	AGATAATCAT
P. xiuningensis	GTCCAAAAAC	TTACTGGTTA	TGATAGGGTT	ATGGTGTATA	AGTTCCATGA	AGATAATCAT
P. xizii	GTCCAAAAAC	TTACTGGTTA	TGATAGGGTT	ATGGTGTATA	AGTTCCATGA	AGATAATCAT
P. yangchunensis	GTCCAAAAAC	TTACTGGGTA	TGATAGGGTT	ATGGTGTATA	AGTTCCATGA	AGATAATCAT
P. yongxingensis	GTCCAAAAAC	TTACTGGTTA	TGATAGGGTT	ATGGTGTATA	AGTTCCATGA	AGATAATCAT
P. yungfuensis	GTCCAAAAAC	TTACTGGTTA	TGATAGGGTT	ATGGTGTATA	AGTTCCATGA	AGATAATCAT
P. cordifolia	GTCCAAAAAC	TTACTGGTTA	TGATAGGGTT	ATGGTGTATA	AGTTCCATGA	AGATAATCAT
P. danxiaensis	GTCCAAAAAC	TTACTGGTTA	TGATAGGGTT	ATGGTGTATA	AGTTCCATGA	AGATAATCAT
P. leiophylla & P. napoensis	GTCCAAAAAC	TTACTGGTTA	TGATAGGGTT	ATGGTGTATA	AGTTCCATGA	AGATAATCAT
P. verecunda	GTCCAAAAAC	TAACTGGTTA	TGATAGGGTT	ATGGTGTATA	AGTTCCATGA	AGATAATCAT
sp. nov. 6	GTTCAAAAAC	TTACTGGTTA	TGATAGGGTT	ATGGTGTATA	AGTTCCATGA	AGATAATCAT
sp. nov. 8	GTCCAAAAAC	TTACTGGTTA	TGATAGGGTT	ATGGTGTATA	AGTTCCATGA	AGATAATCAT
sp. nov. 9	GTCCAAAAAC	TTACTGGTTA	TGATAGGGTT	ATGGTGTATA	AGTTCCATGA	AGATAATCAT
sp. nov. 10	GTCCAAAAAC	TTACTGGTTA	TGATAGGGTT	ATGGTGTATA	AGTTCCACGA	AGATAATCAT
sp. nov. 12	GTCCAAAAAC	TTACTGGTTA	TGATAGGGTT	ATGGTGTATA	AGTTCCATGA	AGATAATCAT
sp. nov. 13	GTCCAAAAAC	TTACTGGTTA	TGATAGGGTT	ATGGTGTATA	AGTTCCATGA	AGATAATCAT
sp. nov. 16	GTCCAAAAAC	TTACTGGTTA	TGATAGGGTT	ATGGTGTATA	AGTTCCATGA	AGATAATCAT
sp. nov. 18	GTCCAAAAAC	TTACTGGTTA	TGATAGGGTT	ATGGTGTATA	AGTTCCATGA	AGATAATCAT
sp. nov. 19	GTCCAAAAAC	TTACTGGTTA	TGATAGGGTT	ATGGTGTACA	AGTTCCATGA	AGATAATCAT
Didymocarpus hancei	GTCCAAAAAC	TTACTGGTTA	TGATAGGGTT	ATGGTGTATA	AGTTCCATGA	AGATAATCAT
Petrocodon dealbatus	GTCCAAAAAC	TCACTGGTTA	TGATAGGGTT	ATGGTGTATA	AGTTCCATGA	AGATAATCAT

	670	680	690	700	710	720
P. bicolor	GGAGAGGTAG	TGTCAGAAAT	TAGAAGGTCC	GACTTAGAAC	CTTATTTGGG	GCTGCACTAT
P. bipinnatifida	GGAGAGGTAG	TGTCAGAAAT	TAGAAGGTCC	GACTTAGAAC	CTTATTTGGG	GCTGCACTAT
P. chizhouensis	GGAGAGGTAG	TGTCAGAAAT	TAGAAGGTCC	GACTTGGAAC	CTTATTTGGG	GCTGCACTAT
P. cordata	GGAGAGGTAC	TGTCAGAAAT	TAGAAGGTCC	GACTTAGAGC	CTTATTTGGG	GCTGCACTAT
P. depressa	GGAGAGGTAG	TGTCAGAAAT	TAGAAGGTCC	GACTTAGAAC	CTTATTTGGG	GCTGCACTAT
P. dongguanica	GGAGAGGTAG	TGTCAGAAAT	TAGAAGGTCC	GACTTAGAGC	CTTATTTGGG	GCTGCACTAT
P. eburnea	GGAGAGGTAG	TGTCAGAAAT	TAGAAGGTCC	GACTTAGAAC	CTTATTTGGG	GCTGCACTAT
P. fimbrisepala	GGAGAGGTAG	TGTCAGAAAT	TAGAAGGTCC	GACTTAGAGC	CTTATTTGGG	GCTGCATTAT
P. glandulosa var. yangshuoens	GGAGAGGTAG	TGTCAGAAAT	TAGAAGGTCC	GACTTAGAGC	CTTATTTGGG	GCTGCACTAT
P. gueilinensis var. brachycar	GGAGAGGTAG	TGTCAGAAAT	TAGAAGGTCC	GACTTAGAAC	CTTATTTGGG	GCTGCACTAT
P.guihaiensis	GGAGAGGTAG	TGTCAGAAAT	TAGAAGGTCC	GACTTAGAGC	CTTATTTGGG	GCTGCACTAT
P. heterotricha & P. pterppoda	GGAGAGGTAG	TGTCAGAAAT	TAGAAGGTCC	GACTTAGAAC	CTTATTTGGG	ACTGCACTAT
P. hochiensis	GGAGAGGTAG	TGTCAGAAAT	TAGAAGGTCC	GACTTAGAGC	CTTATTTGGG	GCTGCACTAT
P. huaijiensis	GGAGAGGTAG	TGTCAGAAAT	TAGAAGGTCC	GACTTAGAAC	CTTATTTGGG	GCTGCACTAT
P. langshanica	GGAGAGGTAG	TGTCAGAAAT	TAGAAGGTCC	GACTTAGAAC	CTTATTTGGG	GCTGCACTAT
P. latinervis	GGAGAGGTAG	TGTCAGAAAT	TAGAAGGTCC	GACTTAGAAC	CTTATTTGGG	GCTGCACTAT
P. laxiflora	GGAGAGGTAG	TGTCAGAGAT	TAGAAGGTCC	GACTTAGAAC	CTTATTTGGG	GCTGCACTAT
P. leprosa	GGAGAGGTAG	TGTCAGAAAT	TAGAAGGTCC	GACTTAGAAC	CTTATTTGGG	GCTGCACTAT
P. liguliformis	GGAGAGGTAG	TGTCAGAAAT	TAGAAGGTCC	GACTTAGAAC	CTTATTTGGG	GCTGCACTAT
P. lijiangensis	GGAGAGGTAG	TGTCAGAAAT	TAGAAGGTCC	GACTTAGAGC	CTTATTTGGG	GCTGCACTAT
P. linearifolia	GGAGAGGTGG	TGTCAGAAAT	TAGAAGGTCC	GACTTAGAAC	CTTATTTGGG	GCTGCACTAT
P. lobulata	GGAGAGGTAG	TGTCAGAAAT	TAGAAGGTCC	GACTTAGAAC	CTTATTTGGG	GCTGCACTAT
P. longii	GGAGAGGTAG	TGTCAGAAAT	TAGAAGGTCC	GACTTAGAAC	CTTATTTGGG	GCTGCACTAT
P. lunglinensis	GGAGAGGTGG	TGTCAGAAAT	TAGAAGGTCC	GACTTAGAAC	CTTATTTGGG	GCTGCACTAT
P. lungzhouensis	GGAGAGGTAG	TGTCAGAAAT	TAGAAGGTCC	GACTTAGAGC	CTTATTTGGG	GCTGCACTAT
P. luochengensis	GGAGAGGTAG	TGTCAGAAAT	TAGAAGGTCC	GACTTAGAAC	CTTATTTGGG	GCTGCACTAT
P. lutea	GGAGAGGTAG	TGTCAGAAAT	TAGAAGGTCC	GACTTAGAAC	CTTATTTGGG	GCTGCACTAT
P. mabaensis	GGAGAGGTAG	TGTCAGAAAT	TAGAAGGTCC	GACTTAGAAC	CTTATTTGGG	GCTGCACTAT
P. macrodonta	GGAGAGGTAG	TGTCAGAAAT	TAGAAGGTCC	GACTTAGAAC	CTTATTTGGG	GCTGCACTAT
P. medica	GGAGAGGTAG	TGTCAGAAAT	TAGAAGGTCC	GACTTAGAGC	CTTATTTGGG	GCTGCACTAT
P. moii	GGAGAGGTAG	TGTCAGAAAT	TAGAAGGTCC	GACTTAGAAC	CTTATTTGGG	GCTGCACTAT
P. mollifolia	GGAGAGGTAG	TGTCAGAAAT	TAGAAGGTCT	GACTTAGAAC	CTTATTTGGG	GCTGCACTAT
P. obtusidentata	GGAGAGGTAG	TGTCGGAAAT	TAGAAGGTCC	GACTTAGAAC	CTTATTTGGG	GCTGCACTAT
P. orthandra	GGAGAGGTAG	TGTCAGAAAT	TAGAAGGTCC	GACTTAGAAC	CTTATTTGGG	GCTGCACTAT
P. parvifolia	GGAGAGGTAG	TGTCAGAAAT	TAGAAGGTCC	GACTTAGAAC	CTTATTTGGG	GCTGCACTAT
P. pinnatifida	GGAGAGGTAG	TGTCAGAAAT	TAGAAGGTCC	GACTTAGAAC	CTTATTTGGG	GCTGCACTAT
P. pulchurfolia	GGAGAGGTAG	TGTCAGAAAT	TAGAAGGTCC	GACTTAGAAC	CTTATTTGGG	GCTGCACTAT
P. renifolia	GGAGAGGTAG	TGTCAGAAAT	TAGAAGGTCT	GACTTAGAAC	CTTATTTGGG	GCTGCACTAT
P. repanda	GGAGAGGTAG	TGTCAGAAAT	TAGAAGGTCC	GACTTAGAAC	CTTATTTGGG	GCTGCACTAT
P. ronganensis	GGAGAGGTAG	TGTCAGAAAT	TAGAAGGTCT	GACTTAGAAC	CTTATTTGGG	GCTGCACTAT
P. sclerophylla	GGAGAGGTAG	TGTCAGAAAT	TAGAAGGTCT	GACTTAGAAC	CTTATTTGGG	GCTGCACTAT
P. sinensis	GGAGAGGTAG	TGTCAGAAAT	CAGAAGGTCC	GACTTAGAAC	CTTATTTGGG	GCTGCACTAT
P. spinulosa	GGAGAGGTAG	TGTCAGAAAT	TAGAAGGTCC	GACTTAGAAC	CTTATTTGGG	GCTGCACTAT
P. subrhomboidea	GGAGAGGTAG	TGTCAGAAAT	TAGAAGGTCC	GACTTAGAGC	CTTATTTGGG	GCTGCACTAT
P. subrhomboidea var. tribract	GGAGAGGTAG	TGTCAGAAAT	TAGAAGGTCC	GACTTAGAAC	CTTATTTGGG	GCTGCACTAT
P. subulata	GGAGAGGTAG	TGTCAGAAAT	TAGAAGGTCC	GACTTAGAAC	CTTATTTGGG	GCTGCACTAT
P. swinglei	GGAGAGGTAG	TGTCAGAGAT	TAGAAGGTCC	GACTTAGAAC	CTTATTTGGG	GCTGCACTAT
P. tabacum	GGAGAGGTAG	TGTCAGAAAT	TAGAAGGTCC	GACTTAGAAC	CTTATTTGGG	GCTGCACTAT
P. tenuifolia	GGAGAGGTAG	TGTCAGAAAT	TAGAAGGTCC	GACTTAGAGC	CTTATTTGGG	GCTGCACTAT
P. tenuituba	GGAGAGGTAG	TGTCAGAAAT	TAGAAGGTCC	GACTTAGAAC	CTTATTTGGG	GCTGCACTAT
P. tiandengensis	GGAGAGGTAG	TGTCAGAAAT	TAGAAGGTCT	GACTTAGAAC	CTTATTTGGG	GCTGCACTAT
P. tribracteata	GGAGAGGTAG	TGTCAGAAAT	TAGAAGGTCT	GACTTAGAAC	CTTATTTGGG	GCTGCACTAT
P. villosissima	GGAGAGGTGG	TGTCAGAAAT	TAGAAGGTCC	GACTTAGAAC	CTTATTTGGG	GCTGCACTAT
P. wentsaii	GGAGAGGTAG	TGTCAGAAAT	TAGAAGGTCC	GACTTAGAAC	CTTATTTGGG	GCTGCACTAT
P. xiuningensis	GGAGAGGTAG	TGTCAGAAAT	TAGAAGGTCC	GACTTAGAAC	CTTATTTGGG	GCTGCACTAT
P. xizii	GGAGAGGTAG	TGTCAGAAAT	TAGAAGGTCC	GACTTAGAAC	CTTATTTGGG	GCTGCACTAT
P. yangchunensis	GGAGAGGTGG	TGTCAGAAAT	TAGAAGGTCC	GACTTAGAAC	CTTATTTGGG	GCTGCACTAT
P. yongxingensis	GGAGAGGTAG	TGTCAGAAAT	TAGAAGGTCC	GACTTAGAAC	CTTATTTGGG	GCTGCACTAT
P. yungfuensis	GGAGAGGTAG	TGTCAGAAAT	TAGAAGGTCC	GACTTAGAAC	CTTATTTGGG	GCTGCACTAT
P. cordifolia	GGAGAGGTAG	TGTCAGAAAT	TAGAAGGTCC	GACTTAGAAC	CTTATTTAGG	GCTGCACTAT
P. danxiaensis	GGAGAGGTAG	TGTCAGAAAT	TAGAAGGTCC	GACTTAGAAC	CTTATTTGGG	GCTGCACTAT
P. leiophylla & P. napoensis	GGAGAGGTAG	TGTCAGAAAT	TAGAAGGTCT	GACTTAGAAC	CTTATTTGGG	GCTGCACTAT
P. verecunda	GGAGAGGTAG	TGTCAGAAAT	TAGAAGGTCC	GACTTAGAGC	CTTATTTGGG	GCTGCACTAT
sp. nov. 6	GGAGAGGTAG	TGTCAGAAAT	TAGAAGGTCT	GACTTAGAAC	CTTATTTGGG	GCTGCACTAT
sp. nov. 8	GGAGAGGTAG	TGTCAGAAAT	TAGAAGGTCC	GACTTAGAAC	CTTATTTGGG	GCTGCACTAT
sp. nov. 9	GGAGAGGTAG	TGTCAGAAAT	TAGAAGGTCC	GACTTAGAAC	CTTATTTGGG	GCTGCACTAT
sp. nov. 10	GGAGAGGTAG	TGTCAGAAAT	TAGAAGGTCC	GACTTAGAAC	CTTATTTGGG	GCTGCACTAT
sp. nov. 12	GGAGAGGTAG	TGTCAGAAAT	TAGAAGGTCC	GACTTAGAAC	CTTATTTGGG	GCTGCACTAT
sp. nov. 13	GGAGAGGTAG	TGTCAGAAAT	TAGAAGGTCC	GACTTAGAAC	CTTATTTGGG	GCTGCACTAT
sp. nov. 16	GGAGAGGTAG	TGTCAGAAAT	TAGAAGGTCC	GACTTAGAAC	CTTATTTGGG	GCTGCACTAT
sp. nov. 18	GGAGAGGTGG	TGTCAGAAAT	TAGAAGGTCC	GACTTAGAAC	CTTATTTGGG	GCTGCACTAT
sp. nov. 19	GGAGAGGTAG	TGTCAGAAAT	TAGAAGGTCC	GACTTAGAAC	CTTATTTGGG	GCTGCACTAT
Didymocarpus hancei	GGAGAGGTAG	TGTCAGAAAT	TAGAAGGTCC	GACTTAGAAC	CTTATTTGGG	GCTGCACTAT
Petrocodon dealbatus	GGAGAGGTAG	TGTCAGAAAT	TAGAAGGTCC	GACTTAGAAC	CTTATTTGGG	GCTGCACTAT

Taxon	730	740	750	760	770	780
P. bicolor	CCATCTACTG	ATATCCCTCA	AGCAGCCCGT	TTCTTGTTCA	TGCAGAACCG	TGTCAGAATG
P. bipinnatifida	CCATCTACTG	ATATCCCTCA	AGCAGCCCGT	TTCTTGTTCA	TGCAGAACCG	TGTCAGAATG
P. chizhouensis	CCATCTACTG	ATATCCCTCA	AGCAGCCCGT	TTCTTGTTCA	TGCAGAACCG	TGTCAGAATG
P. cordata	CCATCTACTG	ATATCCCTCA	AGCAGCCCGT	TTCTTGTTCA	TGCAGAACCG	TGTCAGAATG
P. depressa	CCATCTACTG	ATATCCCTCA	AGCAGCCCGT	TTCTTGTTCA	TGCAGAACCG	TGTCAGAATG
P. dongguanica	CCATCTACTG	ATATCCCTCA	AGCAGCCCGT	TTCTTGTTCA	TGCAGAACCG	TGTCAGAATG
P. eburnea	CCATCTACTG	ATATCCCTCA	AGCAGCCCGT	TTCTTGTTCA	TGCAGAACCG	TGTCAGAATG
P. fimbrisepala	CCATCTACTG	ATATCCCTCA	AGCAGCCCGT	TTCTTGTTCA	TGCAGAACCG	TGTCAGAATG
P. glandulosa var. yangshuoens	CCATCTACTG	ATATCCCTCA	AGCAGCCCGT	TTCTTGTTCA	TGCAGAACCG	TGTCAGAATG
P. gueilinensis var. brachycar	CCATCTACTG	ATATCCCTCA	AGCAGCCCGT	TTCTTGTTCA	TGCAGAACCG	TGTCAGAATG
P.guihaiensis	CCATCTACTG	ATATCCCTCA	AGCAGCCCGT	TTCTTGTTCA	TGCAGAACCG	TGTCAGAATG
P. heterotricha & P. pterppoda	CCATCTACTG	ATATCCCTCA	AACAGCCCGT	TTCTTGTTCA	TGCAGAACCG	TGTCAGAATG
P. hochiensis	CCATCTACTG	ATATCCCTCA	AGCAGCCCGT	TTCTTGTTCA	TGCAGAACCG	TGTCAGAATG
P. huaijiensis	CCATCTACTG	ATATCCCTCA	AGCAGCCCGT	TTCTTGTTCA	TGCAGAACCG	TGTCAGAATG
P. langshanica	CCATCTACTG	ATATCCCTCA	GGCAGCCCGT	TTCCTGTTCA	TGCAGAACCG	TGTCAGAATG
P. latinervis	CCATCTACTG	ATATCCCTCA	AGCAGCCCGT	TTCTTGTTCA	TGCAGAACCG	TGTCAGAATG
P. laxiflora	CCATCTACTG	ATATCCCTCA	AGCAGCCCGT	TTCTTGTTCA	TGCAGAACCG	TGTCAGAATG
P. leprosa	CCATCTACTG	ATATCCCTCA	AGCAGCCCGT	TTCTTGTTCA	TGCAGAACCG	TGTCAGAATG
P. liguliformis	CCATCTACTG	ATATCCCTCA	AGCAGCCCGT	TTCTTGTTCA	TGCAGAACCG	TGTCAGAATG
P. lijiangensis	CCATCTACTG	ATATCCCTCA	AGCAGCCCGT	TTCTTGTTCA	TGCAGAACCG	TGTCAGAATG
P. linearifolia	CCATCTACTG	ATATCCCTCA	AGCAGCCCGT	TTCTTGTTCA	TGCAGAACCG	TGTCAGAATG
P. lobulata	CCATCTACTG	ATATCCCTCA	AGCAGCCCGT	TTCTTGTTCA	TGCAGAACCG	TGTCAGAATG
P. longii	CCATCTACTG	ATATCCCTCA	AGCAGCCCGT	TTCTTGTTCA	TGCAGAACCG	TGTCAGAATG
P. lunglinensis	CCATCTACTG	ATATCCCTCA	AGCAGCCCGT	TTCTTGTTCA	TGCAGAACCG	TGTCAGAATG
P. lungzhouensis	CCATCTACTG	ATATCCCTCA	AGCAGCCCGT	TTCTTGTTCA	TGCAGAACCG	TGTCAGAATG
P. luochengensis	CCATCTACTG	ATATCCCTCA	AGCAGCCCGT	TTCTTGTTCA	TGCAGAACCG	TGTCAGAATG
P. lutea	CCATCTACTG	ATATCCCTCA	AGCAGCCCGT	TTCTTGTTCA	TGCAGAACCG	TGTCAGAATG
P. mabaensis	CCATCTACTG	ATATCCCTCA	AGCAGCCCGT	TTCTTGTTCA	TGCAGAACCG	TGTCAGAATG
P. macrodonta	CCATCTACTG	ATATCCCTCA	AGCAGCCCGT	TTCTTGTTCA	TGCAGAACCG	TGTCAGAATG
P. medica	CCATCTACTG	ATATCCCTCA	AGCAGCCCGT	TTCTTGTTCA	TGCAGAACCG	TGTCAGAATG
P. moii	CCATCCACTG	ATATCCCTCA	AGCAGCCCGT	TTCTTGTTCA	TGCAGAACCG	TGTCAGAATG
P. mollifolia	CCATCTACTG	ATATCCCTCA	AGCAGCCCGT	TTCTTGTTCA	TGCAGAACCG	TGTCAGAATG
P. obtusidentata	CCATCTACTG	ATATCCCTCA	AGCAGCCCGT	TTCTTGTTCA	TGCAGAACCG	TGTCAGAATG
P. orthandra	CCATCTACTG	ATATCCCTCA	AGCAGCCCGT	TTCTTGTTCA	TGCAGAACCG	TGTCAGAATG
P. parvifolia	CCATCTACTG	ATATCCCTCA	AGCAGCCCGT	TTCTTGTTCA	TGCAGAACCG	TGTCAGAATG
P. pinnatifida	CCATCTACTG	ATATCCCTCA	AGCAGCCCGT	TTCTTGTTCA	TGCAGAACCG	TGTCAGAATG
P. pulchurfolia	CCATCTACTG	ATATCCCTCA	AGCAGCCCGT	TTCTTGTTCA	TGCAGAACCG	TGTCAGAATG
P. renifolia	CCATCTACTG	ATATCCCTCA	AGCAGCCCGT	TTCTTGTTCA	TGCAGAACCG	TGTCAGATTG
P. repanda	CCATCTACCG	ATATCCCTCA	AGCAGCCCGT	TTCTTGTTCA	TGCAGAACCG	TGTCAGAATG
P. ronganensis	CCATCTACTG	ATATCCCTCA	AGCAGCCCGT	TTCTTGTTCA	TGCAGAACCG	TGTCAGAATG
P. sclerophylla	CCATCTACTG	ATATCCCTCA	AGCAGCCCGT	TTCTTGTTCA	TGCAGAACCG	TGTCAGAATG
P. sinensis	CCATCTACTG	ATATCCCTCA	AGCAGCCCGT	TTCTTGTTCA	TGCAGAACCG	TGTCAGAATG
P. spinulosa	CCATCTACTG	ATATCCCTCA	AGCAGCCCGT	TTCTTGTTCA	TGCAAAACCG	TGTCAGAATG
P. subrhomboidea	CCATCTACTG	ATATCCCTCA	AGCAGCCCGT	TTCTTGTTCA	TGCAGAACCG	TGTCAGAATG
P. subrhomboidea var. tribract	CCATCTACTG	ATATCCCTCA	AGCAGCCCGT	TTCTTGTTCA	TGCAGAACCG	TGTCAGAATG
P. subulata	CCATCTACTG	ATATCCCTCA	AGCAGCCCGT	TTCTTGTTCA	TGCAGAACCG	TGTCAGAATG
P. swinglei	CCATCTACTG	ATATCCCTCA	AGCAGCCCGT	TTCTTGTTCA	TGCAGAACCG	TGTCAGAATG
P. tabacum	CCATCTACTG	ATATCCCTCA	AGCAGCCCGT	TTCTTGTTCA	TGCAGAACCG	TGTCAGAATG
P. tenuifolia	CCATCCACTG	ATATCCCTCA	AGCAGCCCGT	TTCTTGTTCA	TGCAGAACCG	TGTCAGAATG
P. tenuituba	CCATCTACTG	ATATCCCTCA	AGCAGCCCGT	TTCTTGTTCA	TGCAGAACCG	TGTCAGAATG
P. tiandengensis	CCATCTACTG	ATATCCCTCA	AGCAGCCCGT	TTCTTGTTCA	TGCAGAACCG	TGTCAGAATG
P. tribracteata	CCATCTACTG	ATATCCCTCA	AGCAGCCCGT	TTCTTGTTCA	TGCAGAACCG	TGTCAGAATG
P. villosissima	CCATCTACTG	ATATCCCTCA	AGCAGCCCGT	TTCTTGTTCA	TGCAGAACCG	TGTCAGAATG
P. wentsaii	CCATCTACTG	ATATCCCTCA	AGCAGCCCGT	TTCTTGTTCA	TGCAGAACCG	TGTCAGAATG
P. xiuningensis	CCATCTACTG	ATATCCCTCA	AGCAGCCCGT	TTCTTGTTCA	TGCAGAACCG	TGTCAGAATG
P. xizii	CCATCTACTG	ATATCCCTCA	AGCAGCCCGT	TTCTTGTTCA	TGCAGAACCG	TGTTAGAATG
P. yangchunensis	CCATCTACTG	ATATCCCTCA	AGCAGCCCGT	TTCTTGTTCA	TGCAGAACCG	TGTCAGAATG
P. yongxingensis	CCATCTACTG	ATATCCCTCA	AGCAGCCCGT	TTCTTGTTCA	TGCAGAACCG	TGTCAGAATG
P. yungfuensis	CCATCTACTG	ATATCCCTCA	AGCAGCCCGT	TTCTTGTTCA	TGCAGAACCG	TGTCAGAATG
P. cordifolia	CCATCTACCG	ATATCCCTCA	AGCAGCCCGT	TTCTTGTTCA	TGCAGAACCG	TGTCAGAATG
P. danxiaensis	CCATCTACTG	ATATCCCTCA	AGCAGCCCGT	TTCTTGTTCA	TGCAGAACCG	TGTCAGAATG
P. leiophylla & P. napoensis	CCATCTACTG	ATATCCCTCA	AGCAGCCCGT	TTCTTGTTCA	TGCAGAACCG	TGTCAGAATG
P. verecunda	CCATCTACTG	ATATCCCTCA	AGCAGCCCGT	TTCTTGTTCA	TGCAGAACCG	TGTCAGAATG
sp. nov. 6	CCATCTACTG	ATATCCCTCA	AGCAGCCCGT	TTCTTGTTCA	TGCAGAACCG	TGTCAGAATG
sp. nov. 8	CCATCTACTG	ATATCCCTCA	AGCAGCCCGT	TTCTTGTTCA	TGCAGAACCG	TGTCAGAATG
sp. nov. 9	CCATCTACTG	ATATCCCTCA	AGCAGCCCGT	TTCTTGTTCA	TGCAGAACCG	TGTCAGAATG
sp. nov. 10	CCATCTACTG	ATATCCCTCA	AGCAGCCCGT	TTCTTGTTCA	TGCAGAACCG	TGTCAGAATG
sp. nov. 12	CCATCTACTG	ATATCCCTCA	AGCAGCCCGT	TTCTTGTTCA	TGCAGAACCG	TGTCAGAATG
sp. nov. 13	CCATCTACTG	ATATCCCTCA	AGCAGCCCGT	TTCTTGTTCA	TGCAGAACCG	TGTCAGAATG
sp. nov. 16	CCATCTACTG	ATATCCCTCA	AGCAGCCCGT	TTCTTGTTCA	TGCAGAACCG	TGTTAGAATG
sp. nov. 18	CCATCTACTG	ATATCCCTCA	AGCAGCCCGT	TTCTTGTTCA	TGCAGAACCG	TGTCAGAATG
sp. nov. 19	CCATCTACTG	ATATCCCTCA	AGCAGCCCGT	TTCTTGTTCA	TGCAGAACCG	TGTCAGAATG
Didymocarpus hancei	CCATCCACTG	ATATCCCTCA	AGCAGCCCGT	TTCTTGTTCA	TGCAGAACCG	TGTCAGAATG
Petrocodon dealbatus	CCATCCACTG	ATATCCCTCA	AGCAGCCCGT	TTCTTGTTCA	TGCAGAACCG	TGTCAGAATG

	790	800	810	820	830	840
P. bicolor	ATTTGCGATT	GCAGGGCAAA	GCCAGTTAAG	ATAATCCAAA	GTAAAGAACT	AAAGCAGCCT
P. bipinnatifida	ATTTGCGATT	GCAGGGCAAA	GCCAGTTAAG	ATAATCCAAA	GTAAAGAACT	AAAGCAGCCT
P. chizhouensis	ATTTGCGATT	GCAGGGCAAA	GCCAGTTAAG	ATAATCCAAA	GTAAAGAACT	AAAGCAGCCT
P. cordata	ATTTGCGATT	GCAGGGCAAA	GCCAGTTAAG	ATAATCCAAA	GTAAAGAACT	AAAGCAGCCT
P. depressa	ATTTGCGATT	GCAGGGCAAA	GCCAGTTAAG	ATAATCCAAA	GTAAAGAACT	AAAGCAGCAT
P. dongguanica	ATTTGCGATT	GCAGGGCAAA	GCCAGTTAAG	ATAATCCAAA	GTAAAGAACT	AAAGCAGCCT
P. eburnea	ATTTGCGATT	GCAGGGCAAA	GCCAGTTAAG	ATAATCCAAA	GTAAAGAACT	AAAGCAGCCT
P. fimbrisepala	ATTTGCGATT	GCAGGGCAAA	GCCAGTTAAG	ATAATCCAAA	GTAAAGAACT	AAAGCAGCCT
P. glandulosa var. yangshuoens	ATTTGCGACT	GCAGGGCAAA	GCCAGTTAAG	ATAATCCAAA	GTAAAGAACT	AAAGCAGCCT
P. gueilinensis var. brachycar	ATTTGCGATT	GCAGGGCAAA	GCCAGTTAAG	ATAATCCAAA	GTAAAGAACT	AAAGCAGCCT
P.guihaiensis	ATTTGCGATT	GCAGGGCAAA	GCCAGTTAAG	ATAATCCAAA	GTAAAGAACT	AAAGCAGCCT
P. heterotricha & P. pterppoda	ATTTGCGATT	GCAGGGCAAA	GCCAGTTAAG	ATAATCCAAA	GTAAAGAACT	AAAGCAGCCT
P. hochiensis	ATTTGCGATT	GCAGGGCAAA	GCCAGTTAAG	ATAATCCAAA	GTAAAGAACT	AAAGCAGCCT
P. huaijiensis	ATTTGCGATT	GCAGGGCAAA	GCCAGTTAAG	ATAATCCAAA	GTAAAGAACT	AAAGCAGCCT
P. langshanica	ATTTGCGATT	GCAGGGCAAA	GCCAGTTAAG	ATAATCCAAA	GTAAAGCATT	AAAGCAGCCT
P. latinervis	ATTTGCGATT	GCAGGGCAAA	GCCAGTTAAG	ATAATCCAAA	GTAAAGAACT	AAAGCAGCCT
P. laxiflora	ATTTGCGATT	GCAGGGCAAA	GCCAGTTAAG	ATAATCCAAA	GTAATGAACT	AAAGCAGCCT
P. leprosa	ATTTGTGATT	GCAGGGCAAA	GCCAGTTAAG	ATAATCCAAA	GTAAAGAACT	AAAGCAGCCT
P. liguliformis	ATTTGCGATT	GCAGGGCAAA	GCCAGTTAAG	ATAATCCAAA	GTAAAGAACT	AAAGCAGCCT
P. lijiangensis	ATTTGCGATT	GCAGGGCAAA	GCCAGTTAAG	ATAATCCAAA	GTAAAGAACT	AAAGCAGCCT
P. linearifolia	ATTTGCGATT	GCAGGGCAAA	GCCAGTTAAG	ATAATCCAAA	GTAAAGAGCT	AAAGCAGCCT
P. lobulata	ATTTGCGATT	GCAGGGCAAA	GCCAGTTAAG	ATAATCCAAA	GTAAAGAACT	AAAGCAGCCT
P. longii	ATTTGCGATT	GCAGGGCAAA	GCCAGTTAAG	ATAATCCAAA	GTAAAGAACT	AAAGCAGCCT
P. lunglinensis	ATTTGCGATT	GCAGGGCAAA	GCCAGTTAAG	ATAATCCAAA	GTAAAGAACT	AAAGCAGCCT
P. lungzhouensis	ATTTGCGATT	GCAGGGCAAA	GCCAGTTAAG	ATAATCCAAA	GTAAAGAACT	AAAGCAGCCT
P. luochengensis	ATTTGCGATT	GCAGGGCAAA	GCCAGTTAAG	ATAATCCAAA	GTAAAGAACT	AAAGCAGCCT
P. lutea	ATTTGTGATT	GCAGGGCAAA	GCCAGTTAAG	ATAATCCAAA	GTAAAGAACT	AAAGCAGCCT
P. mabaensis	ATTTGCGATT	GCAGGGCAAA	GCCAGTTAAG	ATAATCCAAA	GTAAAGAACT	AAAGCAGCCT
P. macrodonta	ATTTGCGATT	GCAGGGCAAA	GCCAGTTAAG	ATAATCCAAA	GTAAAGAACT	AAAGCAGCCT
P. medica	ATTTGCGATT	GCAGGGCAAA	GCCAGTTAAG	ATAATCCAAA	GTAAAGAACT	AAAGCAGCCT
P. moii	ATTTCCGATT	GCAGGGCAAA	GCCAGTTAAG	ATAATCCAAA	GTAAAGAACT	AAATCAGCCT
P. mollifolia	ATTTGCGATT	GCAGGGCAAA	ACCAGTTAAG	ATAATCCAAA	GTAAAGAACT	AAAGCAGCCT
P. obtusidentata	ATCTGCGATT	GCAGGGCAAA	GCCAGTTAAG	ATAATCCAAA	GTAAAGAACT	AAAGCAGCCT
P. orthandra	ATTTGCGATT	GCAGGGCAAA	GCCAGTTAAG	ATAATCCAAA	GTAAAGAACT	AAAGCAGCCT
P. parvifolia	ATTTGCGATT	GCAGGGCAAA	GCCAGTTAAG	ATAATCCAAA	GTAAAGAACT	AAAGCAGCCT
P. pinnatifida	ATTTGCGATT	GCAGGGCGAA	GCCAGTTAAG	ATAATCCAAA	GTAAAGAACT	AAAGCAGCCT
P. pulchurfolia	ATTTGCGATT	GCAGGGCAAA	GCCAGTTAAG	ATAATCCAAG	GTAAAGAACT	AAAGCAGCCT
P. renifolia	ATTTGCGATT	GCAGGGCAAA	GCCAGTTAAG	ATAATCCAAA	GTAAAGAACT	AAAGCAGCCT
P. repanda	ATTTGCGATT	GCAGGGCAAA	GCCAGTTAAG	ATAATCCAAA	GTAAAGAACT	AAAGCAGCCT
P. ronganensis	ATTTGCGATT	GCAGGGCAAA	GCCAGTTAAG	ATAATCCAAA	GTAAAGAACT	AAAGCAGCCT
P. sclerophylla	ATTTGCGATT	GCAGGGCAAA	GCCAGTTAAG	ATAATCCAAA	GTAAAGAACT	AAAGCAGCCT
P. sinensis	ATTTGCGATT	GCAGGGCAAA	GCCAGTTAAG	ATAATCCAAA	GTAAAGAACT	AAAGCAGCCT
P. spinulosa	ATTTGCGATT	GCAGGGCAAA	GCCAGTTAAG	ATAATCCAAA	GTAAAGAACT	AAAGCAGCCT
P. subrhomboidea	ATTTGCGATT	GCAGGGCAAA	GCCAGTTAAG	ATAATCCAAA	GTAAAGAACT	AAAGCAGCCT
P. subrhomboidea var. tribract	ATTTGCGATT	GCAGGGCAAA	GCCAGTTAAG	ATAATCCAAA	GTAAAGAACT	AAAGCAGCCT
P. subulata	ATTTGCGATT	GCAGGGCAAA	GCCAGTTAAG	ATAATCCAAA	GTAAAGAACT	AAAGCAGCCT
P. swinglei	ATTTGCGATT	GCAGGGCAAA	GCCAGTTAAG	ATAATCCAAA	GTAAAGAACT	AAAGCAGCCT
P. tabacum	ATTTGTGATT	GCAGGGCAAA	GCCAGTTAAG	ATAATCCAAA	GTAAAGAACT	AAAGCAGCCT
P. tenuifolia	ATTTGCGATT	GCAGGGCAAA	GCCAGTTAAG	ATAATCCAAA	GTAAAGAACT	AAAGCAGCCT
P. tenuituba	ATTTGCGATT	GCAGGGCAAA	GCCAGTTAAG	ATAATCCAAA	GTAAAGAACT	AAAGCAGCCT
P. tiandengensis	ATTTGCGATT	GCAGGGCAAA	GCCAGTTAAG	ATAATCCAAA	GTAAAGAACT	AAAGCAGCCT
P. tribracteata	ATTTGCGATT	GCAGGGCAAA	GCCAGTTAAG	ATAATCCAAA	GTAAAGAACT	AAAGCAGCCT
P. villosissima	ATTTGCGATT	GCAGGGCAAA	GCCAGTTAAG	ATAATCCAAA	GTAAAGAACT	AAAGCAGCCT
P. wentsaii	ATTTGCGATT	GCAGGGCAAA	GCCAGTTAAG	ATAATCCAAA	GTAAAGAACT	AAAGCAGCCT
P. xiuningensis	ATTTGCGATT	GCAGGGCAAA	GCCAGTTATG	ATAATCCAAA	GTAAAGAACT	AAAGCAGCCT
P. xizii	ATTTGCGATT	GCAGGGCAAA	GCCAGTTAAG	ATAATCCAAA	GTAAAGAACT	AAAGCAGCCT
P. yangchunensis	ATTTGCGATT	GCAGGGCAAA	GCCAGTTAAG	ATAATCCAAA	GTAAAGAACT	AAAGCAGCCT
P. yongxingensis	ATTTGCGATT	GCAGGGCAAA	GCCAGTTAAG	ATAATCCAAA	GCAAAGAACT	AAAGCAGCCT
P. yungfuensis	ATTTGCGATT	GCAGGGCGAA	GCCAGTTAAG	ATAATCCAAA	GTAAAGAACT	AAAGCAGCCT
P. cordifolia	ATTTGCGATT	GCAGGGCAAA	GCCAGTTAAG	ATAATCCAAA	GTAAAGAACT	AAAGCAGCCT
P. danxiaensis	ATTTGCGATT	GCAGGGCAAA	GCCAGTTAAG	ATAATCCAAA	GTAAAGAACT	AAAGCAGCCT
P. leiophylla & P. napoensis	ATTTGCGATT	GCAGGGCAAA	GCCAGTTAAG	ATAATCCAAA	GTAAAGAACT	AAAGCAGCCT
P. verecunda	ATTTGCGATT	GCAGGGCAAA	GCCAGTTAAG	ATAATTCAAA	GTAAAGAACT	AAAGCAGCCT
sp. nov. 6	ATTTGCGATT	GCAGGGCAAA	GCCAGTTAAG	ATAATCCAAA	GTAAAGAACT	AAAGCAGCCT
sp. nov. 8	ATTTGCGATT	GCAGGGCAAA	GCCAGTTAAG	ATAATCCAAA	GTAAAGAACT	AAAGCAGCCT
sp. nov. 9	ATTTGCGATT	GCAGGGCAAA	GCCAGTTAAG	ATAATCCAAA	GTAAAGAACT	AAAGCAGCCT
sp. nov. 10	ATTTGCGATT	GCAGGGCAAA	GCCAGTTAAG	ATAATCCAAA	GTAAAGAACT	AAAGCAGCCT
sp. nov. 12	ATTTGCGATT	GCAGGGCAAA	GCCAGTTAAG	ATAATCCAAA	GTAAAGAACT	AAAGCAGCCT
sp. nov. 13	ATTTGCGATT	GCAGGGCAAA	GCCAGTTAAG	ATAATCCAAA	GTAAAGAACT	AAAGCAGCCT
sp. nov. 16	ATTTGCGATT	GCAGGGCAAA	GCCAGTTAAG	ATAATCCAAA	GTAAAGAACT	AAAGCAGCCT
sp. nov. 18	ATTTGCGATT	GCAGGGCAAA	GCCAGTTAAG	ATAATCCAAA	GTAAAGAACT	AAAGCAGCCT
sp. nov. 19	ATTTGCGATT	GCAGGGCAAA	GCCAGTTAAG	ATAATCCAAA	GTAAAGAACT	AAAGCAGCCT
Didymocarpus hancei	ATTTGCGATT	GCAGGGCAAA	GTCAGTTAAG	ATAATCCAAA	GTAAAGAACT	AAAGCAGCCT
Petrocodon dealbatus	ATTTGCGATT	GCAGGGCAAA	GTCTGTTAAG	ATAATCCAAA	GTAAAGAACT	AAAGCAGCCT

	850	860	
P. bicolor	CTCTGCTTGG	TTAATTCTAC	C
P. bipinnatifida	CTTTGCTTGG	GTAATTCTAC	C
P. chizhouensis	CTTTGCTTGG	TTAATTCTAC	C
P. cordata	CTTTGCTTGG	TTAATTCTAC	C
P. depressa	CTTTGCTTGG	TTAATTCTAC	C
P. dongguanica	CTTTGCTTGG	TTAATTCTAC	C
P. eburnea	CTTTGCTTGG	TTAATTCTAC	C
P. fimbrisepala	CTTTGCTTGG	TTAATTCTAC	C
P. glandulosa var. yangshuoens	CTTTGCTTGG	TTAATTCTAC	C
P. gueilinensis var. brachycar	CTTTGCTTGG	TTAATTCTAC	C
P.guihaiensis	CTTTGCTTGG	TTAATTCTAC	C
P. heterotricha & P. pterppoda	CTTTGCTTGA	TTAATTCTAC	C
P. hochiensis	CTTTGCTTGG	TTAATTCTAC	C
P. huaijiensis	CTTTGCTTGG	TTAATTCTAC	C
P. langshanica	CTTTGCTTGG	TTAATTCTAC	C
P. latinervis	CTTTGCTTGG	TTAATTCTAC	C
P. laxiflora	CTTTGCTTGG	TTAATTCTAC	C
P. leprosa	CTTTGCTTGG	TTAATTCTAC	C
P. liguliformis	CTTTGCTTGG	TTAATTCTAC	C
P. lijiangensis	CTTTGCTTGG	TTAATTCTAC	C
P. linearifolia	CTTTGCTTGG	TTAATTCTAC	C
P. lobulata	CTTTGCTTGG	TTAATTCTAC	C
P. longii	CTTTGCTTGG	TTAATTCTAC	C
P. lunglinensis	CTTTGCTTGG	TTAATTCTAC	C
P. lungzhouensis	CTTTGCTTGG	TTAATTCTAC	C
P. luochengensis	CTTTGCTTGG	TTAATTCTAC	C
P. lutea	CTTTGCTTGG	TTAATTCTAC	C
P. mabaensis	CTTTGCTTGG	TTAATTCTAC	C
P. macrodonta	CTTTGCTTGG	TTAATTCTAC	C
P. medica	CTTTGCTTGG	TTAATTCTAC	C
P. moii	CTTTGCTTGG	TTAATTCTAC	C
P. mollifolia	CTTTGCTTGG	TTAATTCTAC	C
P. obtusidentata	CTTTGCTTGG	TTAATTCTAC	C
P. orthandra	CTTTGCTTGG	TTAATTCTAC	C
P. parvifolia	CTTTGCTTGG	TTAATTCTAC	C
P. pinnatifida	CTTTGCTTGG	TTAATTCTAC	C
P. pulchurfolia	CTTTGCTTGG	TTAATTCTAC	C
P. renifolia	CTTTGCTTGG	TTAATTCTAC	C
P. repanda	CTTTGCTTGG	TTAATTCTAC	C
P. ronganensis	CTTTGCTTGG	TTAATTCTAC	C
P. sclerophylla	CTTTGCTTGG	TTAATTCTAC	C
P. sinensis	CTTTGCTTGG	TTAATTCTAC	C
P. spinulosa	CTTTGCTTGG	TTAATTCTAC	C
P. subrhomboidea	CTTTGCTTGG	TTAATTCTAC	C
P. subrhomboidea var. tribract	CTTTGCTTGG	TTAATTCTAC	C
P. subulata	CTTTGCTTGG	TTAATTCTAC	C
P. swinglei	CTTTGCTTGG	TTAATTCTAC	C
P. tabacum	CTTTGCTTGG	TTAATTCTAC	C
P. tenuifolia	CTTTGCTTGG	TTAATTCTAC	C
P. tenuituba	CTTTGCTTGG	TTAATTCTAC	C
P. tiandengensis	CTTTGCTTGG	TTAATTCTAC	C
P. tribracteata	CTTTGCTTGG	TTAATTCTAC	C
P. villosissima	CTTTGCTTGG	TTAATTCTAC	C
P. wentsaii	CTTTGCTTGG	TTAATTCTAC	C
P. xiuningensis	CTTTGCTTGG	TTAATTCTAC	C
P. xizii	CTTTGCTTGG	TTAATTCTAC	C
P. yangchunensis	CTTTGCTTGG	TTAATTCTAC	C
P. yongxingensis	CTTTGCTTGG	TTAATTCTAC	C
P. yungfuensis	CTTTGCTTGG	TTAATTCTAC	C
P. cordifolia	CTTTGCTTGG	TTAATTCTAC	C
P. danxiaensis	CTTTGCTTGG	TTAATTCTAC	C
P. leiophylla & P. napoensis	CTTTGCTTGG	TTAATTCTAC	C
P. verecunda	CTTTGCTTGG	TTAATTCTAC	C
sp. nov. 6	CTTTGCTTGG	TTAATTCTAC	C
sp. nov. 8	CTTTGCTTGG	TTAATTCTAC	C
sp. nov. 9	CTTTGCTTGG	TTAATTCTAC	C
sp. nov. 10	CTTTGCTTGG	TTAATTCTAC	C
sp. nov. 12	CTTTGCTTGG	TTAATTCTAC	C
sp. nov. 13	CTTTGCTTGG	TTAATTCTAC	C
sp. nov. 16	CTTTGCTTGG	TTAATTCTAC	C
sp. nov. 18	CTTTGCTTGG	TTAATTCTAC	C
sp. nov. 19	CTTTGCTTGG	TTAATTCTAC	C
Didymocarpus hancei	CTTTGCTTGG	TTAATTCTAC	C
Petrocodon dealbatus	CTTTGCTTAG	TTAATTCTAC	C
