# Supplementary material for: Adaptive Molecular Evolution of PHYE in Primulina, a Karst Cave Plant
Source: PLoS One. 2015 Jun 1;10(6):e0127821. doi: 10.1371/journal.pone.0127821 (PMC4452542; doi:10.1371/journal.pone.0127821)
Supplement: S2 File — (PDF) [file pone.0127821.s003.pdf]

S3. Alignment of 10 full-length sequences of *PHYE*.

	10	20	30	40	50
P.eburnea	ATGGAGTTAG	AAACCGAATA	TGGTAAAAAC	TCGACAGCCA	CAAATCGAAA
P.fimbrisepala	ATGGAGTTAG	AAACCGAATA	TGGTAAAAAC	TCGACAGCCA	CAAATCGAAA
P.heterotricha	ATGGAATTAG	AAACCGAAAA	TGGTAAAAAC	CCGACAGCCA	CAAATCGAAA
P.huaijiensis	ATGGAGTTAG	AAACCGAATA	TGGTAAAAAC	TCGACAGCCA	CAAATCGAAA
P.lutea	ATGGAGTTAG	AAACCGAATA	TGGTAAAAAC	TCGACAGCCA	CAAATCGAAA
P.pteropoda	ATGGAGTTAG	AAACCGAAAA	TGGTAAAAAC	CCGACAGCCA	CAAATCGAAA
P.sinensis	ATGGAGCTAG	AAACCGAATA	TGGTCAGAAC	TCGACAGCCA	CAAATCGAAA
P.swinglei	ATGGAGTTAG	AAACCGAAAA	TGGTAAAAAC	CCGACAGCCA	CAAATCGAAA
P.tabacum	ATGGAGTTAG	AAACCGAATA	TGGTAAAAAC	TCGACAGCCA	CAAATCGAAA
P.villosissima	ATGGAGTTAG	AAACCGAATA	TGGTAAAAAC	TCGACAGCCA	CAAATCGAAA

	60	70	80	90	100
P.eburnea	ATTGGACGAA	AGCAAGGGAA	AAAATAACGC	CGCCAACACT	GTTTTGTCAT
P.fimbrisepala	ATTGGACGAA	AGCACGGGAA	AAAATAACGC	CTCCAACACT	GTTTTGTCAT
P.heterotricha	ATTCGACGAA	AGCAAGGGAA	AAAATAACGA	CGCCAACACT	GTTTTGTCAT
P.huaijiensis	ATTGCACGAA	AGCAAGGGAA	AAAATAACGC	CGCCAACACT	GTTTTGGCAT
P.lutea	ATTGGACGAA	AGCAAGGGAA	AAAATAACGC	CGCCAACACT	GTTTTGTCAT
P.pteropoda	ATTCGACGAA	AGCAAGGGAA	AAAATAACGT	CGCCAACACT	GTTTTGTCAT
P.sinensis	ATTGCACGAA	AGCAAGGGAA	AAAATAACGC	CACCAACACT	GTTTTGTCAT
P.swinglei	ATTGGACGAA	AGCAAGGGAA	AAAATAGCGT	CGCCAACACT	GTTTTGTCAT
P.tabacum	ATTGGACGAA	AGCAAGGGAA	AAAATAACGC	TACCAACGCT	GTTTTGTCAT
P.villosissima	ATTAGACGAA	AGCAAGGGAA	AAAATAACGC	CTCCAACACT	GTTTTGTCAT

	110	120	130	140	150
P.eburnea	CCTCTGCTGC	TAGCAACATG	AACAACAACA	GAGCTATGGC	TCAGTACAAT
P.fimbrisepala	CCTCTGCTGC	TAGCAACATG	AACACCAACA	GAGCTATGGC	TCAATACAAT
P.heterotricha	CCTCTGCTGC	TAGCAACATG	AACACCAACA	GAGCCATGGT	TCAGTACAAT
P.huaijiensis	CCTCTGCTGC	TAGCAACATG	AACACCAACA	GAGCTATGGC	TCAGTACAAT
P.lutea	TCTCTGCTGC	TAGCAACATG	AACACCAACA	GAGCTATGGC	TCAGTACAAT
P.pteropoda	CCTCTGCTGC	TAGCAACATG	AACACCAACA	GAACCATGGT	TCAGTACAAT
P.sinensis	CCTCTGCTGC	TAGCAACATG	AACACCAACA	GAGCTATGGC	TCAGTACAAT
P.swinglei	CCTCTGCTGC	AAGCAACATG	AACACCAACA	GAGCTATGGC	TCAGTACAAT
P.tabacum	CCTCTGCTGC	TAGCAACATG	AACACCAACA	GAGCTATGGC	TCAGTACAAT
P.villosissima	CCTCTGCTGC	TAGCAACATG	AACACCAACA	GAGCTATGGC	TCAGTACAAT

	160	170	180	190	200
P.eburnea	GCCGATGCGA	GGCTGATGGC	TGAATTTGAG	CAGTCCGGTA	AGTCTGGTAA
P.fimbrisepala	GCCGATGCGA	GGCTGATGGC	TGAATTTGAG	CAGTCCGGTA	AGTCTGGTAA
P.heterotricha	GCCGATGCGA	GGCTGATGGC	TGAATTTGAG	CAGTCCGGTA	AGTCGGGTAA
P.huaijiensis	GCCGATGCGA	GGCTGATGGC	TGAATTTGAG	CAGTCCGGTA	AGTCTGGTAA
P.lutea	GCCGATGCGA	GGCTGATGGC	TGAATTTGAG	CAGTCTGGTA	AGTCTGGTAA
P.pteropoda	GCCGATGCGA	GGCTGATGGC	TGAATTTGAG	CAGTCCGGTA	AGTCGGGTAA
P.sinensis	GCCGATGCGA	GGCTGATGGC	TGAATTTGAG	CAGTCCGGTA	AGTCTGGTAA
P.swinglei	GCCGATGCGA	GGCTGATGGC	TGAATTTGAG	CAGTCCGGTA	AGTCAGGTAA
P.tabacum	GCCGATGCGA	GGCTGATGGC	TGAATTTGAG	CAGTCTGGTA	AGTCTGGTAA
P.villosissima	GCCGATGCGA	GGCTGATGGC	TGAATTTGAG	CAGTCCGGTA	AGTCTGGTAA

	210	220	230	240	250
P.eburnea	GTTCTTTAAC	TACTCAAAGT	CAGTTTCTCA	TGCTCCGAAT	ACTTTGAGCA
P.fimbrisepala	GTTCTTTAAC	TACTCAAAGT	CAGTTTCTCA	TGCTCCGAAT	ACTTTGAGCA
P.heterotricha	GTTCTTTAAC	TACTCGAAGT	CAGTTTCTCA	TGCTCCGAAT	ACTTTGAGCA
P.huaijiensis	GTTCTTTAAC	TACTCAAAGT	CAGTTTCTCA	TGCTCCGAAT	ACTTTGAGCA
P.lutea	GTTCTTTAAC	TACTCAAAGT	CAGTTTCTCA	TGCTCCGAAT	ACTTTGAGCA
P.pteropoda	GTTCTTTAAC	TACTCAAAGT	CAGTTTCTCA	TGCTCCGAAT	ACTTTGAGCA
P.sinensis	GTTCTTTAAC	TACTCTAAGT	CAGTTTCTCA	TGCTCCGAAT	ACTTTGAGCA
P.swinglei	GTTCTTTAAC	TACTCGAAGT	CAGTTTCTCA	TGCTCCCAAT	ACTTTGAGCA
P.tabacum	GTTCTTTAAC	TACTCAAAGT	CAGTTTCTCA	TGCTCCGAAT	ACTTTGAGCA
P.villosissima	GTTCTTTAAC	TACTCAAAGT	CAGTTTCTCA	TGCTCCGAAT	ACTTTGAGCA

	260	270	280	290	300
P.eburnea	CTGAGGAGGA	GATGACTGCT	TATTTGTCGA	AAATCCAGAG	GGGGAGTCTT
P.fimbrisepala	CTGAGGAGGA	GATGATTGCT	TATTTGTCGA	AAATCCAGAG	GGGGAGTCTT
P.heterotricha	CTGAGGAGGA	GATGACTGCT	TATTTGTCGA	AAATCCAGAG	GGGGAGTCTT
P.huaijiensis	CTGAGGAGGA	GATGACTGCT	TATTTGTCGA	AAATCCAGAG	GGGGAGTCTT
P.lutea	CTGAGGAGGA	GATGACTGCT	TATTTGTCGA	AAATCCAGAG	GGGGAGTCTT
P.pteropoda	CTGAGGAGGA	GATGACTGCT	TATTTGTCGA	AAATCCAGAG	GGGGAGTCTT
P.sinensis	CTGAGGAGGA	GATGACTGCT	TATTTGTCGA	AAATCCAGAG	GGGGAGTCTT
P.swinglei	CTGAGGAGGA	GATGACTGCT	TATTTGTCGA	AAATCCAGAG	GGGAAGTCTT
P.tabacum	CTGAGGAGGA	GATGACTGCT	TATTTGTCGA	AAATCCAGAG	GGGGAGTCTT
P.villosissima	CTGAGGAGGA	GATGATTGCT	TATTTGTCGA	AAATCCAGAG	GGGGAGTCTT

	310	320	330	340	350
P.eburnea	GTGCAGGCCT	TTGGTTGTAT	GCTTGCGGTC	GAGGAGCCCA	GTTTGAAGAT
P.fimbrisepala	GTGCAGGCCT	TTGGTTGTAT	GCTTGCGGTC	GAGGAGCCCA	GTTTGAAGAT
P.heterotricha	GTGCAGGCCT	TTGGTTGTAT	GCTTACGGTC	GAGGAGCCCA	GTTTGAAGAT
P.huaijiensis	GTGCAGGCCT	TTGGTTGTAT	GCTTGCGGTC	GAGGAGCCCA	GTTTGAAGAT
P.lutea	GTGCAGGCCT	TTGGTTGTAT	GCTTGCGGTC	GAGGAGCCCA	GTTTGAAGAT
P.pteropoda	GTGCAGGCCT	TTGGTTGTAT	GCTTGCGGTC	GAGGAGCCCA	GTTTGAAGAT
P.sinensis	GTGCAGGCCT	TTGGTTGTAT	GCTTGCGGTC	GAGGAGCCCA	GTTTGAAGAT
P.swinglei	GTTCAGGCCA	TTGGTTGTAT	GCTTGCGGTC	GAGGAGCCCA	GTTTAAAGAT
P.tabacum	GTGCAGGCCT	TTGGTTGTAT	GCTTGCGGTC	GAGGAGCCCA	GTTTGAAGAT
P.villosissima	GTGCAGGCCT	TTGGTTGTAT	GCTTGCGGTC	GAGGAGCCCA	GTTTGAAGAT

	360	370	380	390	400
P.eburnea	CATAGGTTAT	AGTGAGAATT	GCTTTGATAT	GCTGGGCTTG	AAGAGTGTCG
P.fimbrisepala	CATAGGTTAT	AGTGAGAATT	GCTTTGATAT	GCTGGGTTTG	AAGAGCGTCG
P.heterotricha	CATAGGTTAT	AGTGAGAATT	GCTTTGATAT	GCTGGGTTTG	AAGAGTGTCG
P.huaijiensis	CATAGGTTAT	AGTGAGAATT	GCTTTGATAT	GCTGGGTTTG	AAGAGCGTCG
P.lutea	CATAGGTTAT	AGTGAGAATT	GCTTTGATAT	GCTGGGCTTG	AAGAGTGTCG
P.pteropoda	CATAGGTTAT	AGTGAGAATT	GCTTTGATAT	GCTGGGTTTG	AAGAGTGTCG
P.sinensis	CATAGGTTAT	AGTGAGAATT	GCTTTGATAT	GCTGGGTTTG	AAGAGCGTCG
P.swinglei	CATAGGTTAT	AGTGAGAATT	GCTTTGATAT	GCTGGGTTTG	AAGAGTGTTG
P.tabacum	TATAGGTTAT	AGTGAGAATT	GCTTTGATAT	GCTGGGGTTG	AAGAGTGTCG
P.villosissima	CATAGGTTAT	AGTGAGAATT	GCTTTGATAT	GCTGGGTTTG	AAGAGCGTCG

	410	420	430	440	450
P.eburnea	TTGAGCCAAA	AAAATTGATG	GGTCTTATTG	GGGTTGATGC	AAGAACGCTT
P.fimbrisepala	TCGAGCCAAA	AAAATTGATG	GGTCTTATTG	GGGTTGATGC	AAGAACGCTT
P.heterotricha	TTGAGCCAAA	AAAATTGATG	GGTCTTCTTG	GGGTTGATGC	AAGAACGCTT
P.huaijiensis	TTGAGCCAAA	AAAATTGATG	GGTCTTATTG	GGGTTGATGC	AAGAACGCTT
P.lutea	TTGAGCCAAA	AAAATTGATG	GGTCTTATTG	GGGTTGATGC	AAGAACGCTT
P.pteropoda	TTGAGCCAAA	AAAATTGATG	GGTCTTCTTG	GGGTTGATGC	AAGAACGCTT
P.sinensis	TTGAGCCAAA	AAAATTGATG	GGTCTTATTG	GGGTTGATGC	AAGAACGCTT
P.swinglei	TTGAGCCAAA	AAAATTGATG	GGTCTTATTG	GGGTTGATGC	AAGAACGCTT
P.tabacum	TTGAGCCAAA	AAAATTGATG	GGTCTTATTG	GGGTTGATGC	AAGAACGCTT
P.villosissima	TTGAGCCAAA	AAAATTGATG	GGTCTTATTG	GGGTTGATGC	AAGAACGCTT

	460	470	480	490	500
P.eburnea	TTTACCTCTT	CAAGTAGGGC	TTCATTGGAT	AAAGCTGTGG	CATCAAGGGA
P.fimbrisepala	TTTACCTCTT	CAAGTAGGGC	TTCATTGGAT	AAAGCTGTGG	CATCAAGGGA
P.heterotricha	TTTACCTCTT	CAAGTAGGGA	TTCATTGGAT	AAAGCTGTGG	CATCAAGGGA
P.huaijiensis	TTTACCTCTT	CAAGTAGGGC	TTCATTGGAT	AAAGCTGTGG	CATCAAGGGA
P.lutea	TTTACCTCTT	CAAGTAGGGC	TTCATTGGAT	AAAGCTGTGG	CATCAAGGGA
P.pteropoda	TTTACCTCTT	CAAGTAGGGA	TTCATTGGAT	AAAGCTGTGG	CATCAAGGGA
P.sinensis	TTTACCTCTT	CAAGTAGGGC	TTCGTTGGAT	AAAGCTGTGG	CATCAAGGGA
P.swinglei	TTTACCTCTT	CAAGTAGGGC	TTCGTTGGAT	AAAGCTGTGG	CATCGAGGGA
P.tabacum	TTTACCTCTT	CAAGTAGGGC	TTCATTGGAT	AAAGCTGTGG	CATCAAGGGA
P.villosissima	TTTACCTCTT	CAAGTAGGGC	TTCATTGGAT	AAAGCTGTGG	CATCAAGGGA

	510	520	530	540	550
P.eburnea	AATTTCATTT	TTGAACCCGA	TTTGGGTTCA	TTCTTGCACC	ACCCACAAAC
P.fimbrisepala	AATTTCATTT	TTGAACCCGA	TTTGGGTTCA	TTCTTGCACC	ACCCACAAAC
P.heterotricha	AATTTCATTT	TTGAACCCGA	TTTGGGTTCA	TTCTTGCACC	ACCCACAAAC
P.huaijiensis	AATTTCATTT	TTGAACCCGA	TTTGGGTTCA	TTCTTGCACC	ACCCACAAAC
P.lutea	AATTTCATTT	TTGAACCCGA	TTTGGGTTCA	TTCTTGCACC	ACCCACAAAC
P.pteropoda	AATTTCATTT	TTGAACCCGA	TTTGGGTTCA	TTCTTGCACC	ACCCACAAAC
P.sinensis	AATTTCATTT	TTGAACCCGA	TTTGGGTTCA	TTCTTGCACC	ACCCACAAAC
P.swinglei	AATTTCATTT	TTGAACCCGA	TTTGGGTTCA	TTCTTGCACC	ACCCACAAAC
P.tabacum	AATTTCATTT	TTGAACCCGA	TTTGGGTTCA	TTCTTGCACC	ACCCACAAAC
P.villosissima	AATTTCATTT	TTGAACCCGA	TTTGGGTTCA	TTCTTGCACC	ACCCACAAAC

	560	570	580	590	600
P.eburnea	CATTTTATGC	TATATTACAC	AGAATTGACG	TGGGCATTGT	GGTTGATCTG
P.fimbrisepala	CATTTTATGC	TATATTACAC	AGAATTGATG	TGGGCATTGT	GATTGATCTG
P.heterotricha	CATTTTATGC	TATATTACAC	AGAATTGATG	TGGGCATTGT	GATTGATCTG
P.huaijiensis	CATTTTATGC	TATATTACAC	AGAATTGACG	TGGGCATTGT	GGTTGATCTG
P.lutea	CATTTTATGC	TATATTACAC	AGAATTGACG	TGGGCATTGT	GGTTGATCTG
P.pteropoda	CATTTTATGC	TATATTACAC	AGAATTGATG	TGGGCATTGT	GATTGATCTG
P.sinensis	CATTTTATGC	TATATTACAC	AGAATTGATG	TGGGCATTGT	GATTGATCTG
P.swinglei	CATTTTATGC	TATATTACAC	AGAATTGATG	TGGGCATTGT	GATTGATCTG
P.tabacum	CATTTTATGC	TATATTACAC	AGAATTGACG	TGGGCATTGT	GGTTGACCTG
P.villosissima	CATTTTATGC	TATATTACAC	AGAATTGATG	TGGGCATTGT	GATTGATCTG

	610	620	630	640	650
P.eburnea	GAGCCAGCTC	GTGCTTGTGA	TCCTGCTATG	TTGCATGCTA	GTGCTGTGCA
P.fimbrisepala	GAGCCAGCTC	GTGCTTGCGA	TCCTGCTATG	TTGCATGCTA	GTGCTGTGCA
P.heterotricha	GAGCCAGCTC	GTGCTTGTGA	TCCTGCTATG	TTGCATGCTA	GTGCTGTGCA
P.huaijiensis	GAGCCAGCTC	GTGCTTGTGA	TCCTGCTATG	TTGCATGCTA	GTGCTGTGCA
P.lutea	GAGCCAGCTC	GTGCTTGTGA	TCCTGCTATG	TTGCATGCTA	GTGCTGTGCA
P.pteropoda	GAGCCAGCTC	GTGCTTGTGA	TCCTGCTATG	TTGCATGCTA	GTGCTGTGCA
P.sinensis	GAGCCAGCTC	GTGCTTGTGA	TCCTGCTATG	TTGCATGCTA	GTGCTGTACA
P.swinglei	GAGCCAGCTC	GTGCTTGTGA	TCCTTCTATG	TTGCATGCTA	GTGCTGTGCA
P.tabacum	GAGCCAGCTC	GTGCTTGTGA	TCCTGCTATG	TTGCATGCTA	GTGCTGTGCA
P.villosissima	GAGCCAGCTC	GTGCTTGTGA	TCCTGCTATG	TTGCATGCTA	GTGCTGTGCA

	660	670	680	690	700
P.eburnea	ATCGCAGAAA	CTAGCTGTGA	GAGCCATATC	TAGGCTGCAG	TCTCTTCCTG
P.fimbrisepala	ATCGCAGAAA	CTAGCTGTGA	GAGCCATATC	TAGGCTGCAG	TCTCTTCCTG
P.heterotricha	ATCGCAGAAA	CTAGCCGTGA	GAGCCATATC	TAGGCTGCAG	TCTCTTCCTG
P.huaijiensis	ATCGCAGAAA	CTAGCTGTGA	GAGCCATATC	TAGGCTGCAG	GCTCTTCCTG
P.lutea	ATCGCAGAAA	CTAGCTGTGA	GAGCCATATC	TAGGCTGCAG	TCTCTTCCTG
P.pteropoda	ATCGCAGAAA	CTAGCCGTGA	GAGCCATATC	TAGGCTGCAG	TCTCTTCCTG
P.sinensis	ATCGCAGAAA	CTAGCTGTGA	GAGCCATATC	TAGGCTGCAG	TCTCTTCCTG
P.swinglei	ATCGCAGAAA	CTAGCTGTGA	GAGCCATATC	TAGGCTGCAG	TCTCTTCCTG
P.tabacum	ATCGCAGAAA	CTAGCTGTGA	GAGCCATATC	TAGGCTGCAG	TCTCTTCCTG
P.villosissima	ATCGCAGAAA	CTAGCTGTGA	GAGCCATATC	TAGGCTGCAG	TCTCTTCCTG

	710	720	730	740	750
P.eburnea	GGGGCGATGT	AGGAGTTTTG	TGTGATACAG	TCGTAGAAGA	TGTCCAAAAA
P.fimbrisepala	GGGGCGATGT	TGGAGTTTTG	TGTGATACAG	TCGTAGAAGA	TGTCCAAAAA
P.heterotricha	GGGGCGATGT	AGGAGTTTTG	TGTGATACAG	TCGTAGAAGA	TATCCAAAAA
P.huaijiensis	GGGGCGATGT	AGGAGTTTTG	TGTGATACAG	TCGTAGAAGA	TGTCCAAAAA
P.lutea	GGGGCGATGT	AGGAGTTTTG	TGTGATACAG	TCGTAGAAGA	TGTCCAAAAA
P.pteropoda	GGGGCGATGT	AGGAGTTTTG	TGTGATACAG	TCGTAGAAGA	TATCCAAAAA
P.sinensis	GGGGCGATGT	AGGAGTTTTG	TGTGATACAG	TCGTAGAAGA	TGTCCAAAAA
P.swinglei	GGGGCGATGT	AGGAGTTTTG	TGTGATACAG	TCGTAGAAGA	TGTCCAAAAA
P.tabacum	GGGGCGATGT	AGGAGTTTTG	TGTGATACAG	TCGTAGAAGA	TGTCCAAAAA
P.villosissima	GGGGCGATGT	AGGAGTTTTG	TGTGATACAG	TCGTAGAAGA	TGTCCAAAAA

	760	770	780	790	800
P.eburnea	CTTACTGGTT	ATGATAGGGT	TATGGTGTAT	AAGTTCCATG	AAGATAATCA
P.fimbrisepala	CTAACTGGTT	ATGATAGGGT	TATGGTGTAT	AAGTTCCATG	AAGATAATCA
P.heterotricha	CTTACTGGTT	ATGATAGGGT	TATGGTGTAT	AAGTTCCATG	AAGATAATCA
P.huaijiensis	CTTACTGGTT	ATGATAGGGT	TATGGTGTAT	AAGTTCCATG	AAGATAATCA
P.lutea	CTTACTGGTT	ATGATAGGGT	TATGGTGTAT	AAGTTCCATG	AAGATAATCA
P.pteropoda	CTTACTGGTT	ATGATAGGGT	TATGGTGTAT	AAGTTCCATG	AAGATAATCA
P.sinensis	CTTACTGGTT	ATGATAGGGT	TATGGTGTAT	AAGTTCCATG	AAGATAATCA
P.swinglei	CTTACTGGTT	ATGACAGGGT	TATGGTGTAT	AAGTTCCATG	AAGATAATCA
P.tabacum	CTTACTGGTT	ATGATAGGGT	TATGGTGTAT	AAGTTCCATG	AAGATAATCA
P.villosissima	CTTACTGGTT	ATGATAGGGT	TATGGTGTAT	AAGTTCCATG	AAGATAATCA

	810	820	830	840	850
P.eburnea	TGGAGAGGTA	GTGTCAGAAA	TTAGAAGGTC	CGACTTAGAA	CCTTATTTGG
P.fimbrisepala	TGGAGAGGTA	GTGTCAGAAA	TTAGAAGGTC	CGACTTAGAG	CCTTATTTGG
P.heterotricha	TGGAGAGGTA	GTGTCAGAAA	TTAGAAGGTC	CGACTTAGAA	CCTTATTTGG
P.huaijiensis	TGGAGAGGTA	GTGTCAGAAA	TTAGAAGGTC	CGACTTAGAA	CCTTATTTGG
P.lutea	TGGAGAGGTA	GTGTCAGAAA	TTCGAAGGTC	CGACTTAGAA	CCTTATTTGG
P.pteropoda	TGGAGAGGTA	GTGTCAGAAA	TTAGAAGGTC	CGACTTAGAA	CCTTATTTGG
P.sinensis	TGGAGAGGTA	GTGTCAGAAA	TCAGAAGGTC	CGACTTAGAA	CCTTATTTGG
P.swinglei	TGGAGAGGTA	GTGTCAGAGA	TTAGAAGGTC	CGACTTAGAA	CCTTATTTGG
P.tabacum	TGGAGAGGTA	GTGTCAGAAA	TTAGAAGGTC	CGACTTAGAA	CCTTATTTGG
P.villosissima	TGGAGAGGTG	GTGTCAGAAA	TTAGAAGGTC	CGACTTAGAA	CCTTATTTGG

	860	870	880	890	900
P.eburnea	GGCTGCACTA	TCCATCTACT	GATATCCCTC	AAGCAGCCCG	TTTCTTGTTC
P.fimbrisepala	GGCTGCACTA	TCCATCTACT	GATATCCCTC	AAGCAGCCCG	TTTCTTGTTC
P.heterotricha	GACTGCACTA	TCCATCTACT	GATATCCCTC	AAACAGCCCG	TTTCTTGTTC
P.huaijiensis	GGCTGCACTA	TCCATCTACT	GATATCCCTC	AAGCAGCCCG	TTTCTTGTTC
P.lutea	GGCTGCACTA	TCCATCTACT	GATATCCCTC	AAGCAGCCCG	TTTCTTGTTC
P.pteropoda	GACTGCACTA	TCCATCTACT	GATATCCCTC	AAACAGCCCG	TTTCTTGTTC
P.sinensis	GGCTGCACTA	TCCATCTACT	GATATCCCTC	AAGCAGCCCG	TTTCTTGTTC
P.swinglei	GGCTGCACTA	TCCATCTACT	GATATCCCTC	AAGCAGCCCG	TTTCTTGTTC
P.tabacum	GGCTGCACTA	TCCATCTACT	GATATCCCTC	AAGCAGCCCG	TTTCTTGTTC
P.villosissima	GGCTGCACTA	TCCATCTACT	GATATCCCTC	AAGCAGCCCG	TTTCTTGTTC

	910	920	930	940	950
P.eburnea	ATGCAGAACC	GTGTCAGAAT	GATTTGCGAT	TGCAGGGCAA	AGCCAGTTAA
P.fimbrisepala	ATGCAGAACC	GTGTCAGAAT	GATTTGCGAT	TGCAGGGCAA	AGCCAGTTAA
P.heterotricha	ATGCAGAACC	GTGTCAGAAT	GATTTGCGAT	TGCAGGGCAA	AGCCAGTTAA
P.huaijiensis	ATGCAGAACC	GTGTCAGAAT	GATTTGCGAT	TGCAGGGCAA	AGCCAGTTAA
P.lutea	ATGCAGAACC	GTGTCAGAAT	GATTTGCGAT	TGCAGGGCAA	AGCCAGTTAA
P.pteropoda	ATGCAGAACC	GTGTCAGAAT	GATTTGCGAT	TGCAGGGCAA	AGCCAGTTAA
P.sinensis	ATGCAGAACC	GTGTCAGAAT	GATTTGCGAT	TGCAGGGCAA	AGCCAGTTAA
P.swinglei	ATGCAGAACC	GTGTCAGAAT	GATTTGCGAT	TGCAGGGCAA	AGCCAGTTAA
P.tabacum	ATGCAGAACC	GTGTCAGAAT	GATTTGTGAT	TGCAGGGCAA	AGCCAGTTAA
P.villosissima	ATACAGAACC	GTGTCAGAAT	GATTTGCGAT	TGCAGGGCAA	AGCCAGTTAA

	960	970	980	990	1000
P.eburnea	GATAATCCAA	AGTAAAGAAC	TAAAGCAGCC	TCTTTGCTTG	GTTAATTCTA
P.fimbrisepala	GATAATCCAA	AGTAAAGAAC	TAAAGCAGCC	TCTTTGCTTG	GTTAATTCTA
P.heterotricha	GATAATCCAA	AGTAAAGAAC	TAAAGCAGCC	TCTTTGCTTG	ATTAATTCTA
P.huaijiensis	GATAATCCAA	AGTAAAGAAC	TAAAGCAGCC	TCTTTGCTTG	GTTAATTCTA
P.lutea	GATAATCCAA	AGTAAAGAAC	TAAAGCAGCC	TCTTTGCTTG	GTTAATTCCA
P.pteropoda	GATAATCCAA	AGTAAAGAAC	TAAAGCAGCC	TCTTTGCTTG	ATTAATTCTA
P.sinensis	GATAATCCAA	AGTAAAGAAC	TAAAGCAGCC	TCTTTGCTTG	GTTAATTCTA
P.swinglei	GATAATCCAA	AGTAAAGAAC	TAAAGCAGCC	TCTTTGCTTG	GTTAATTCTA
P.tabacum	GATAATCCAA	AGTAAAGAAC	TAAAGCAGCC	TCTTTGCTTG	GTTAATTCTA
P.villosissima	GATAATCCAA	AGTAAAGAAC	TAAAGCAGCC	TCTTTGCTTG	GTTAATTCTA

	1010	1020	1030	1040	1050
P.eburnea	CCCTACGTTC	ACCACATGGC	TGCCACACTC	AGTACATGGA	GAACATGGGG
P.fimbrisepala	CCCTACGTTC	ACCGCATGGC	TGCCACACTC	AGTACATGGA	GAACATGGGG
P.heterotricha	CCCTACGTTC	GCCACATGGC	TGCCACACTC	AGTACATGGA	GAACATGGGG
P.huaijiensis	CCCTACGTTC	ACCGCATGGC	TGCCACACTC	AGTACATGGA	AAACATGGGG
P.lutea	CCCTACGTTC	GCCGCATGGC	TGCCACACTC	AGTACATGGA	GAACATGGGG
P.pteropoda	CCCTACGTTC	GCCACATGGC	TGCCACACTC	AGTACATGGA	GAACATGGGG
P.sinensis	CCCTACGTTC	ACCGCATGGC	TGCCACACTC	AGTACATGGA	GAACATGGGG
P.swinglei	CCCTACGTTC	GCCGCATGGC	TGCCACACTC	AGTACATGGA	GAACATGGGG
P.tabacum	CCCTTCGTTC	ACCGCATGGC	TGCCACACTC	AGTACATGGA	GAACATGAGG
P.villosissima	CCCTACGTTC	ACCGCACGGC	TGCCACACTC	AGTACATGGA	GAACATGGGG

	1060	1070	1080	1090	1100
P.eburnea	TCCATGGCTT	CATTGGCGAT	GGCGGTAGTT	GTGAATAGCG	GTGATTCGAT
P.fimbrisepala	TCCATGGCTT	CATTGGCGAT	GGCGGTAGTT	GTGAATAGCG	GTGATTCGAT
P.heterotricha	TCCATGGCTT	CATTGGTGAT	GGCGGTAGTT	GTGAATAGCG	GTGATTCGAT
P.huaijiensis	TCCATGGCTT	CATTGGCGAT	GGCGGTAGTT	GTGAATAGCG	GTGATTCGAT
P.lutea	TCCATGGCTT	CATTGGCGAT	GGCGGTAGTT	GTGAATAGCG	GTGATTCGAT
P.pteropoda	TCCATGGCTT	CATTGGTGAT	GGCGGTAGTT	GTGAATAGCG	GTGATTCGAT
P.sinensis	TCCATGGCTT	CATTGGCGAT	GGCGGTAGTT	GTGAATAGCG	GTGATTCGAT
P.swinglei	TCCATGGCTT	CGTTGGCGAT	GGCGGTAGTT	GTGAATAGTG	GTGATTCGAT
P.tabacum	TCCATGGCTT	CATTGGCGAT	GGCGGTAGTT	GTGAATAGCG	GCGATTCGAT
P.villosissima	TCCATGGCTT	CATTGGCGAT	GGCGGTAGTT	GTGAATAGCG	GTGATTCGAT

	1110	1120	1130	1140	1150
P.eburnea	GAAACTATGG	GGATTGGTGG	TGTGCCACCA	CACTTCATCG	CGCTATGTCC
P.fimbrisepala	GAAACTATGG	GGATTGGTGG	TGTGCCACCA	CACTTCATCA	CGCTATGTCC
P.heterotricha	GAAACTATGG	GGATTGGTGG	TGTGCCACCA	CACTTCATCA	CGCTATGTCC
P.huaijiensis	GAAACTATGG	GGATTGGTGG	TGTGCCACCA	CACTTCATCA	CGCTATGTCC
P.lutea	GAAACTATGG	GGATTGGTGG	TGTGCCACCA	CACTTCATCG	CGCTATGTCC
P.pteropoda	GAAACTATGG	GGATTGGTGG	TGTGCCACCA	CACTTCATCA	CGCTATGTCC
P.sinensis	GAAACTATGG	GGATTGGTGG	TGTGCCACCA	CACTTCATCA	CGCTATGTCC
P.swinglei	GAAACTATGG	GGATTGGTGG	TGTGCCACCA	CACTTCATCA	CGCTATGTCC
P.tabacum	GAAACTATGG	GGATTGGTGG	TGTGCCACCA	CACTTCATCG	CGCTATGTCC
P.villosissima	GAAACTATGG	GGATTGGTGG	TGTGTCACCA	CACTTCATCA	CGCTATGTCC

	1160	1170	1180	1190	1200
P.eburnea	CTTTTCCACT	TCGGTACGCT	TGTGAATTCC	TTATGCAGGC	ATTTGGTCTG
P.fimbrisepala	CTTTTCCACT	TCGGTATGCT	TGTGAGTTCC	TTATGCAGGC	ATTTGGCCTG
P.heterotricha	CTTTTCCACT	TCGGTATGCT	TGTGAGTTCC	TCATGCAGGC	ATTTGGTCTG
P.huaijiensis	CTTTTCCACT	TCGGTATGCT	TGTGAGTTCC	TTATGCAGGC	ATTTGGTCTG
P.lutea	CTTTTCCACT	TCGGTACGCT	TGTGAGTTCC	TTATGCAGGC	ATTTGGTCTG
P.pteropoda	CTTTTCCACT	TCGGTATGCT	TGTGAGTTCC	TCATGCAGGC	ATTTGGTCTG
P.sinensis	CTTTTCCACT	TCGGTATGCT	TGTGAGTTCC	TTATGCAGGC	ATTTGGTCTG
P.swinglei	CTTTTCCACT	TCGGTATGCT	TGTGAGTTCC	TTATGCAGGC	ATTTGGTCTG
P.tabacum	CTTTTCCACT	TCGGTATGCT	TGTGAGTTCC	TTATGCAGGC	ATTTGGTCTG
P.villosissima	CTTTTCCACT	TCGGTATGCT	TGTGAGTTCC	TTATGCAGGC	ATTTGGTCTG

	1210	1220	1230	1240	1250
P.eburnea	CAGCTTAATA	TGGAGCTTCA	ATTAGCATCA	CAGTTGGTGG	AAAAGAAAAC
P.fimbrisepala	CAGCTTAATA	TGGAGCTTCA	ATTAGCATCA	CAGTTGGTGG	AAAAGAAAAC
P.heterotricha	CAGCTTAATA	TGGAGCTTCA	ATTAGCATCA	CAGTTGGTGG	AAAAGAAAAC
P.huaijiensis	CAGCTTAATA	TGGAGCTTCA	ATTAGCATCA	CAGTTGGTGG	AAAAGAAAAC
P.lutea	CAGCTTAATA	TGGAGCTTCA	ATTAGCATCA	CAGTTGGTGG	AAAAGAAAAC
P.pteropoda	CAGCTTAATA	TGGAGCTTCA	ATTAGCATCA	CAGTTGGTGG	AAAAGAAAAC
P.sinensis	CAGCTTAACA	TGGAGCTTCA	ATTAGCATCA	CAGTTGGTGG	AAAAGAAAAC
P.swinglei	CAGCTTAATA	TGGAGCTTCA	ATTAGCATCA	CAGTTGGTGG	AAAAGAAAAC
P.tabacum	CAGCTTAATA	TGGAGCTTCA	ATTAGCATCA	CAGTTGGTGG	AAAAGAAAAC
P.villosissima	CAGCTTAATA	TGGAGCTTCA	ATTAGCATCA	CAGTTGGTGG	AAAAGAAAAC

	1260	1270	1280	1290	1300
P.eburnea	CCTTCGGATG	CAAACCTTAT	TGTGCGAGAT	GCTTCTTCGC	GGAGCTCCGT
P.fimbrisepala	CCTTCGGATG	CAAACCTTAT	TGTGCGAGAT	GCTTCTTCGC	GGAGCTCCGT
P.heterotricha	CCTTCGGATG	CAAACCTTAT	TGTGCGAGAT	GCTTCTTCGT	GGAGCTCCGT
P.huaijiensis	CCTTCGGATG	CAAACCTTAT	TGTGCGAGAT	GCTTCTTCGC	GGAGCTCCGT
P.lutea	CCTTCGGATG	CAAACCTTAT	TGTGCGAGAT	GCTTCTTCGT	GGAGCTCCTT
P.pteropoda	CCTTCGGATG	CAAACCTTAT	TGTGCGAGAT	GCTTCTTCGT	GGAGCTCCGT
P.sinensis	CCTTCGGATG	CAAACCTTAT	TGTGCGAGAT	GCTTCTTCGC	GGAGCTCCGT
P.swinglei	CCTTCGGATG	CAAACCTTAT	TGTGCGAGAT	GCTTCTTCGC	GGAGCTCCGT
P.tabacum	CCTTCGGATG	CAAACCTTAT	TGTGCGAGAT	GCTTCTTCGC	GGAGCTCCGT
P.villosissima	CCTTCGGATG	CAAACCTTAT	TGTGCGAGAT	GCTTCTTCGC	GGAGCTCCGT

	1310	1320	1330	1340	1350
P.eburnea	TTGGGATTGT	GAATCAGTCT	CCCAATATCA	TGGATCTTGT	AAATTGTGAC
P.fimbrisepala	TTGGGATTGT	GAATCAGTCT	CCCAATATCA	TGGATCTTGT	AAATTGTGAC
P.heterotricha	TTGGGATTGT	GAATCAGTCT	CCCAATATCA	TGGATCTTGT	AAATTGTGAC
P.huaijiensis	TTGGGATTGT	GAATCAGTCT	CCCAATATCA	TGGATCTTGT	AAATTGTGAC
P.lutea	TTGGGATTGT	GAATCAGTCT	CCCAATATTA	TGGATCTTGT	AAATTGTGAC
P.pteropoda	TTGGGATTGT	GAATCAGTCT	CCCAATATCA	TGGATCTTGT	AAATTGTGAC
P.sinensis	TTGGGATTGT	GAATCAGTCT	CCCAATATTA	TGGATCTTGT	AAATTGTGAC
P.swinglei	TTGGGATTGT	GAACCAGTCT	CCCAATATCA	TGGATCTTGT	AAATTGTGAC
P.tabacum	TTGGGATTGT	GAATCAGTCT	CCCAATATCA	TGGATCTTGT	AAATTGTGAC
P.villosissima	TTGGGATTGT	GAATCAGTCT	CCCAATATCA	TGGATCTTGT	AAATTGTGAC

	1360	1370	1380	1390	1400
P.eburnea	GGGGCTGCAC	TATATTACCG	TGGGAAATGT	TGGTTTCTTG	GTGTCACACC
P.fimbrisepala	GGGGCTGCAT	TATATTACTG	TGGGAAATGT	TGGTTTCTTG	GTGTCACACC
P.heterotricha	GGGGCAGCAT	TATATTACTG	TGGGAAATGT	TGGTTTCTTG	GTGTCACACC
P.huaijiensis	GGGGCTGCAT	TATATTACTG	TGGGAAATGT	TGGTTTCTTG	GTGTCACACC
P.lutea	GGGGCTGCAT	TATATTACCG	TGGGAAATGT	TGGTTTCTTG	GTGTCACACC
P.pteropoda	GGGGCAGCAT	TATATTACTG	TGGGAAATGT	TGGTTTCTTG	GTGTCACACC
P.sinensis	GGGGCTGCAT	TATATTACTG	TGGTAAATGT	TGGTTTCTTG	GTGTCACACC
P.swinglei	GGGGCTGCAT	TATATTATTG	TGGGAAATGT	TGGTTTCTCG	GTGTCACACC
P.tabacum	GGGGCTGCAT	TATATTACTG	TGGGAAATGT	TGGTTTCTTG	GTGTCACACC
P.villosissima	GGGGCTGCAT	TATATTACTG	TGGGAAATGT	TGGTTTCTTG	GTGTCACACC

	1410	1420	1430	1440	1450
P.eburnea	GACTGAGGCA	CAAATGAGAG	ATATTGCTGA	ATGGCTACTA	AACAGCCTTG
P.fimbrisepala	GACTGAGGCA	CAAGTGAGAG	ATATTGCTGA	ATGGCTACTA	AACAGCCTTG
P.heterotricha	GACTGAGGCA	CAAATGAGAG	ATATTGCTGA	ATGGCTACTA	AACAGCCTTG
P.huaijiensis	GACTGAGGCA	CAAATGAGAG	ATATTGCTGA	ATGGCTACTA	AACAGCCTTG
P.lutea	GACTGAGGCA	CAAATGAGAG	ATATTGCTGA	ATGGCTACTA	AACAGCCTTG
P.pteropoda	GACTGAGGCA	CAAATGAGAG	ATATTGCTGA	ATGGCTACTA	AACAGCCTTG
P.sinensis	GACTGAGGCA	CAAATGAGAG	ATATTGCTGA	ATGGCTACTA	AACAGCCTTG
P.swinglei	GACTGAGGCA	CAACTGAGAG	ATATTGCTGA	ATGGCTACTA	AACAGCCTTG
P.tabacum	GACCGAGGCA	CAAATGAGAG	ATATTGCTGA	ATGGCTACTA	AACAGCCTTG
P.villosissima	GACTGAGGCA	CAAGTGAGAG	ATATTGCTGA	ATGGTTACTA	AACAGCCTTG

	1460	1470	1480	1490	1500
P.eburnea	AAGATTCCAC	AGGGTTAAGT	ACAGATAGTC	TTGCTGATGC	TGGCTATCCA
P.fimbrisepala	AAGATTCCAC	AGGGTTAAGT	ACAGATAGTC	TTGCTGATGC	TGGCTATCCA
P.heterotricha	AAGATTCCAC	AGGGTTAAGT	ACAGATAGTC	TTGCTGATGC	TGGCTATCCA
P.huaijiensis	AAGATTCCAC	AGGGTTAAGT	ACAGATAGTC	TTGCTGGTGC	TGGCTATCCA
P.lutea	AAGATTCCAC	AGGGTTAAGT	ACAGATAGTC	TTGCTGATGC	TGGCTATCCA
P.pteropoda	AAGATTCCAC	AGGGTTAAGT	ACAGATAGTC	TTGCTGATGC	TGGCTATCCA
P.sinensis	AAGATTCCAC	AGGGTTAAGT	ACAGATAGTC	TTGCTGATGC	TGGCTATCCA
P.swinglei	AAGATTCCAC	AGGGTTAAGT	ACAGATAGTC	TTGCTGATGC	TGGCTATCCA
P.tabacum	AAGATTCCAC	AGGGTTAAGT	ACAGATAGTC	TTGCTGACGC	TGGCTATCCA
P.villosissima	AAGATTCCAC	AGGGTTAAGT	ACAGATAGTC	TTGCTGATGC	TGGCTATCCA

	1510	1520	1530	1540	1550
P.eburnea	GGTGCACCAC	TGCTTGGTGA	CGCAGTTTGT	GGTATGATTG	CTGCAAAAAT
P.fimbrisepala	GGTGCAGCAC	TGCTTGGTGA	CGCAGTTTGT	GGCATGATTG	CTGCAAAAAT
P.heterotricha	GGTGCAGCAC	TGCTTGGTGA	CGCAGTTTGT	GGCATGATTG	CTGCAAGAAT
P.huaijiensis	GGTGCAGCAC	TGCTTGGTGA	CGCAGTTTGT	GGCATGATTG	CTGCAAAAAT
P.lutea	GGTGCAGCAC	TGCTTGGTGA	CGCAGTTTGT	GGCATGATTG	CTGCAAAAAT
P.pteropoda	GGTGCAGCAC	TGCTTGGTGA	CGCAGTTTGT	GGCATGATTG	CTGCAAGAAT
P.sinensis	GGTGCAGCAC	TGCTTGGTGA	CGCAGTTTGT	GGCATGATTG	CTGCAAAAAT
P.swinglei	GGTGCAGCAC	TGCTTGGTGA	CGCAGTTTGT	GGCATGATTG	CGGCAAGAAT
P.tabacum	GGTGCAGCAC	TGCTTGGTGA	CACAGTTTGT	GGCATGATTG	CTGCAAGAAT
P.villosissima	GGTGCAGCAC	TGCTTGGTGA	CGCAGTTTGT	GGCATGATTG	CTGCAAAAAT

	1560	1570	1580	1590	1600
P.eburnea	TACATCCACC	GATTTTCTAT	TTTGGTTTAG	GTCTCACACG	GCAAAGGAAA
P.fimbrisepala	TACATCCACC	GATTTTCTAT	TTTGGTTTAG	ATCTCACACG	GCAAAGGAAA
P.heterotricha	TACATCCACC	GATTTTCTAT	TTTGGTTTAG	GTCTCACACG	GCAAAGGAAG
P.huaijiensis	TACATCCACC	GATTTTCTAT	TTTGGTTTAG	GTCTCACACG	GCAAAGGAAA
P.lutea	TACATCCACC	GATTTTCTAT	TTTGGTTTAG	GTCTCACACG	GCAAAGGAAA
P.pteropoda	TACATCCACC	GATTTTCTAT	TTTGGTTTAG	GTCTCACACG	GCAAAGGAAG
P.sinensis	TACATCCACC	GATTTTCTAT	TTTGGTTTAG	GTCTCACACG	GCAAAGGAAG
P.swinglei	TACATCCACC	GATTTTCTAT	TTTGGTTTAG	GTCTCACACG	GCAAAGGAAG
P.tabacum	TACATCCACC	GATTTTCTAT	TTTGGTTTAG	GTCTCACACG	GCAAAGGAAA
P.villosissima	TACATCCACC	GATTTTCTAT	TTTGGTTTAG	GTCTCACACG	GCAAAGGAAA

	1610	1620	1630	1640	1650
P.eburnea	TCAAATGGGG	AGGAGCTAAG	CATCATCCGG	AGGACAAAGA	TGACGGTGGG
P.fimbrisepala	TCAAATGGGG	AGGAGCTAAG	CATCATCCGG	AGGACAAAGA	TGACGGTGGG
P.heterotricha	TCAAATGGGG	AGGAGCTAAG	CATCACCCGG	AGGACAAAGA	TGATGGTGGG
P.huaijiensis	TCAAATGGGG	AGGAGCTAAG	CATCATCCGG	AGGACAAAGA	TGACGGCGGG
P.lutea	TCAAATGGGG	AGGAGCTAAG	CATCATCCAG	AGGACAAAGA	TGACGGTGGG
P.pteropoda	TCAAATGGGG	AGGAGCTAAG	CATCACCCGG	AGGACAAAGA	TGATGGTGGG
P.sinensis	TCAAATGGGG	AGGAGCTAAG	CATCATCCGG	AGGACAAAGA	TGACGGTGGG
P.swinglei	TCAAATGGGG	AGGAGCTAAG	CATCATCCGG	AGGACAAAGA	TGACGGTGGG
P.tabacum	TCAAATGGGG	AGGAGCTAAG	CATCATCCGG	AGGACAAAGA	TGACGGTGGG
P.villosissima	TCAAATGGGG	AGGAGCTAAG	CATCATCCGG	AGGACAAAGA	TGACGGTGGG

	1660	1670	1680	1690	1700
P.eburnea	AAAATGCACC	CCAGATCTTC	ATTCAATACC	TTTCTTGAAG	TAGTGAAAAG
P.fimbrisepala	AAAATGCACC	CTAGATCTTC	ATTCAATACC	TTTCTTGAAG	TAGTAAAAAG
P.heterotricha	AAAATGCACC	CTAGATCTTC	ATTCAATACC	TTTCTTGAAG	TAGTAAAAAG
P.huaijiensis	AAAATGCACC	CTAGATCTTC	ATTCAATACC	TTTCTTGAAG	TAGTAAAAAG
P.lutea	AAAATGCACC	CTAGATCTTC	ATTCAATACC	TTTCTTGAAG	TAGTAAAAAG
P.pteropoda	AAAATGCACC	CTAGATCTTC	ATTCAATACC	TTTCTTGAAG	TAGTAAAAAG
P.sinensis	AAAATGCACC	CTAGATCTTC	ATTCAATACC	TTTCTTGAAG	TAGTAAAAAG
P.swinglei	AAAATGCACC	CTAGATCTTC	ATTCAATACC	TTTCTTGAAG	TAGTAAAAAG
P.tabacum	AAAATGCACC	CTAGATCTTC	ATTCAATACC	TTTCTTGAAG	TAGTAAAAAG
P.villosissima	AAAATGCACC	CTAGATCTTC	ATTCAACACC	TTTCTTGAAG	TAGTAAAAAG

	1710	1720	1730	1740	1750
P.eburnea	CCGGAGTTTG	CAGTGGGAGG	TTGCAGAGAT	TAATGCAATT	CATTCTCTCC
P.fimbrisepala	CCGGAGTTTG	CAGTGGGAGG	TTGCAGAGAT	TAATGCAATT	CATTCTCTCC
P.heterotricha	CCGGAGTTTG	CAGTGGGAGG	TTGCAGAGAT	TAATGCAATT	CATTCTCTCC
P.huaijiensis	CCGGAGTTTG	CAGTGGGAGG	TTGCAGAGAT	TAATGCAATT	CATTCTCTCC
P.lutea	CCGGAGTTTG	CAGTGGGAGG	TTGCAGAGAT	TAATGCAATT	CATTCTCTCC
P.pteropoda	CCGGAGTTTG	CAGTGGGAGG	TTGCAGAGAT	TAATGCAATT	CATTCTCTCC
P.sinensis	CCGGAGCTTG	CAGTGGGAGG	TTGCAGAGAT	TAATGCAATT	CATTCTCTCC
P.swinglei	CCGGAGTTTG	CAGTGGGAGG	TTGCAGAGAT	TAATGCAATT	CATTCTCTCC
P.tabacum	CCGGAGTTTG	CATTGGGAGG	TTGCAGAGAT	TAATGCAATT	CATTCTCTCC
P.villosissima	CCGGAGTTTG	CAGTGGGAGG	TTGCAGAGAT	TAATGCAATT	CATTCTCTCC

	1760	1770	1780	1790	1800
P.eburnea	AACTTATATT	GAGAAATTCA	TTCCTGGAGA	TTGAAGAAGG	CAGTCCTAAA
P.fimbrisepala	AACTTATATT	GAGAAATTCG	TTCCTGGAGA	TTGAAGAAGG	CGGCCCTAAA
P.heterotricha	AACTTATATT	GAGAAATTCA	TTCCTGGAGA	TTGAAGAAAG	CGGTCCTAAA
P.huaijiensis	AACTTATATT	GAGAAATTCA	TTCCTGGAGA	TTGAAGAAGG	CGGTCCTAAA
P.lutea	AACTTATATT	GAGAAATTCG	TTCCTGGAGA	TGGAAGAAGG	CGGTCCTAAA
P.pteropoda	AACTTATATT	GAGAAATTCA	TTCCTGGAGA	TTGAAGAAAG	CGGTCCTAAA
P.sinensis	AACTTATATT	GAGAAATTCG	TTCCTGGAGA	TTGAAGAAAG	CGGTCCTAAA
P.swinglei	AACTTATATT	GAGAAATTCG	TTCCTGGAGA	TTGAAGAAAA	CGGTCCTAAA
P.tabacum	AACTTATATT	GAGAAATTCG	TTCCTGGAGA	TTGAAGAAGG	CGGTCCTAAA
P.villosissima	AACTTATATT	GAGAAATTCG	TTCCTGGAGA	TTGAAGAAGG	CGGTCCTAAA

	1810	1820	1830	1840	1850
P.eburnea	CCTGATATAT	TTTCTCAACA	AAATGATTCT	GAGAACCCAC	AGCTGAATGA
P.fimbrisepala	CCTGATATAT	TTTCTCAACA	AAATGATTCT	GACAACCCAC	AGCTGAATGA
P.heterotricha	CCTGATATAT	TTTCTCAACA	AAATGATTCT	GAGAATCCAC	AGCTGGATGA
P.huaijiensis	CCTGATATAT	TTTCTCAACA	AAATGATTCT	GACAACCCAC	AGCTGAATGA
P.lutea	CCTGATATAT	TTTCTCAACA	AAATGATTCT	GACAACCCAC	AGCTGAATGA
P.pteropoda	CCTGATATAT	TTTCTCAACA	AAATGATTCT	GAGAATCCAC	AGCTGGATGA
P.sinensis	CCTGATATAT	TTTCTCAACA	AAATGATTCT	GACAACCCAC	AGCTGGATGA
P.swinglei	CCTGATATAT	TTTCTCAACA	AAATGATTCT	GACAACCCAC	AGCTGGATGA
P.tabacum	CCTGATATAT	TTTCTCAACA	AAATGATTCT	GACAACCCAC	AGCTGAATGA
P.villosissima	CCTGATATAT	TTTCTCAACA	AAATGATTCT	GACAACCCAC	AGCTGAATGA

	1860	1870	1880	1890	1900
P.eburnea	ACTTACTTCG	GCCGCTGCTG	AAATGGTCCG	GCTGATAGAA	ACAGCTACTG
P.fimbrisepala	ACTTACTTCC	GCTGCTGCTG	AAATGGTCCG	GCTGATAGAA	ACAGCTACTG
P.heterotricha	ACTTACTTCC	GCCGCTGCTG	AAATGGTCCG	GTTGATAGAA	ACAGCTACTG
P.huaijiensis	ACTTACTTCC	GCTGCCGCTG	AAATGGTCCG	GCTGATAGAA	ACAGCTACTG
P.lutea	ACTTACTTCG	GCCGCTGCTG	AAATGGTCCG	GCTGATAGAA	ACAGCTACTG
P.pteropoda	ACTTACTTCC	GCCGCCGCTG	AAATGGTCCG	GTTGATAGAA	ACAGCTACTG
P.sinensis	ACTTACTTCC	GCCGCTGCTG	AAATGGTCCG	GCTGATAGAA	ACAGCTACTG
P.swinglei	ACTAACTTCC	GCCGCTGCTG	AAATGGTCCG	GCTGATCGAA	ACAGCTACTG
P.tabacum	ACTTACTTCC	GCCGCTGCTG	AAATGGTCCG	GCTGATAGAA	ACAGCTACTG
P.villosissima	ACTTACTTCC	GCTGCTGTTG	AAATGGTCCG	GCTGATAGAA	ACAGCTACTG

	1910	1920	1930	1940	1950
P.eburnea	TTCCAATTTT	TGGAGTCGAT	GCATCCGGTT	GGATCAATGG	GTGGAATGCT
P.fimbrisepala	TTCCAATTTT	TGGAGTCGAT	TCATCCGGTT	GGATCAATGG	GTGGAATGCT
P.heterotricha	TTCCAATTTT	TGGAGTAGAT	TCATCCGGTT	GGATCAATGG	GTGGAATGCT
P.huaijiensis	TTCCAATTTT	TGGAGTCGAT	TCATCCGGTT	GGATCAATGG	GTGGAATGCT
P.lutea	TTCCAATTTT	TGGAGTCGAT	GCATCCGGTT	GGATCAATGG	GTGGAATGCT
P.pteropoda	TTCCAATTTT	TGGAGTAGAT	TCATCCGGTT	GGATCAATGG	GTGGAATGCT
P.sinensis	TTCCAATTTT	TGGAGTCGAT	TCATCCGGTT	GGATCAATGG	GTGGAATGCT
P.swinglei	TTCCAATTTT	TGGAGTAGAT	TCATCCGGTT	GGATCAATGG	GTGGAATGCT
P.tabacum	TTCCAATTTT	TGGAGTTGAT	TCATCCGGTT	GGATCAATGG	GTGGAATGCT
P.villosissima	TTCCAATTTT	TGGAGTCGAT	TCATCCGGTT	GGATCAATGG	GTGGAATGCT

	1960	1970	1980	1990	2000
P.eburnea	AAGATGCATG	AGTTGACGGG	GTTGACTCTG	TCCGAAGCTT	TGGGAAAGTC
P.fimbrisepala	AAGATGCATG	AGTTGACGGG	GTTGACTCTG	TCCGAAGCTT	TGGGAAAGTC
P.heterotricha	AAGATGCATG	AGTTGACAGG	GTTGACTCTG	TCCGAAGCTT	TGGGAAAGTC
P.huaijiensis	AAGATGCATG	AGTTGACGGG	GTTGACTCTG	TCCGAAGCTT	TGGGAAAGTC
P.lutea	AAGATGCATG	AGTTGACGGG	GTTGACTCTG	TCCGAAGCTT	TGGGAAAGTC
P.pteropoda	AAGATGCATG	AGTTGACAGG	GTTGACTCTG	TCCGAAGCTT	TGGGAAAGTC
P.sinensis	AAGATGCATG	AGTTGACGGG	GTTGACTCTG	TCCGAAGCTT	TGGGAAAGTC
P.swinglei	AAGATGCATG	AATTGACGGG	GTTGACTCTG	TCCGAAGCTT	TGGGAAAGTC
P.tabacum	AAGATGCATG	AGTTGACGGG	GTTGACTCTG	TCTGAAGCTT	TGGGAAAGTC
P.villosissima	AAGATGCATG	AGTTGACGGG	GTTGACTCTG	TCCGAAGCTT	TGGGAAAGTC

	2010	2020	2030	2040	2050
P.eburnea	TCTGATCAAG	GATGTCATTC	ATGAAGACTC	GCGTGGAGCT	ACTGAAATCC
P.fimbrisepala	TCTGATCAAC	GATGTCATTC	ATGAAGACTC	GCGTGGTGCT	GCTGAAATCC
P.heterotricha	TCTGATCAAC	GATGTCATTC	ATGAAGACTC	GCGTGGAGCT	GCTGAAATCC
P.huaijiensis	TCTGATCAAC	GATGTCATTC	ATGAAGACTC	GCGTGGAGCT	GCTGAAATCC
P.lutea	TCTGATCAAC	GATGTCATTC	ATGAAGACTC	GCGTGGAGCT	ACTGAAATCC
P.pteropoda	TCTGATCAAC	GATGTCATTC	ATGAAGACTC	GCGTGGAGCT	GCTGAAATCC
P.sinensis	TCTGATCAAC	GATGTCATTC	ATGAAGACTC	GCGTGGAGCT	GCTGAAATCC
P.swinglei	TCTGATCAAC	GATGTCATTC	ATGAAGACTC	GCGTGGAGCT	GCTGAAATCC
P.tabacum	TCTGATCAAC	GATGTCATTC	ACGAAGACTC	GCGTGGAGCT	ACTGAAATCC
P.villosissima	TTTGATCAAC	GATGTCATTC	ACGAAGACTC	GCGTGGAGCT	GCTGAAATCC

	2060	2070	2080	2090	2100
P.eburnea	TGCTGCAAAG	AGCTCTGCAC	GGTGAGGAGG	AAAAAAATGT	TGAAGTGAAG
P.fimbrisepala	TGCTGCAAAG	AGCTCTGCAC	GGTGAGGAGC	AAAAAAATGT	TGAAGTGAAG
P.heterotricha	TGCTGCAAAG	AGCTCTGCAC	GGTGAGGAGG	AAAAAAATGT	TGAAGTGAAG
P.huaijiensis	TGTTGCAAAG	AGCTCTGCAC	GGTGAGGAGA	AAAAAAATGT	TGAAGTGAAG
P.lutea	TGCTGCAAAG	AGCTCTGCAC	GGCGAGGAGG	AAAAAAATGT	TGAAGTGAAG
P.pteropoda	TGCTGCAAAG	AGCTCTGCAA	GGTGAGGAGG	AAAAAAATGT	TGAAGTGAAG
P.sinensis	TGCTGCAAAG	AGCTCTGCAC	GGCGAGGAGG	AAAAAAATGT	TGAAGTGAAG
P.swinglei	TGCTGCAAAG	AGCTCTGCAC	GGTGAGGAGG	AAAAAAACGT	TGAAGTGAAG
P.tabacum	TGCTGCAAAG	AGCTCTGCAC	GGTGAGGAGG	AAAAAAATGT	TGAAGTGAAG
P.villosissima	TGCTGCAAAG	AGCTCTGCAC	GGTGAGGAGG	AAAAAAATGT	TGAAGTGAAG

	2110	2120	2130	2140	2150
P.eburnea	CTAATGACTT	TTGGGGGACA	TGTTCCAAAC	GCTGTTATCC	TTGCTAATGC
P.fimbrisepala	CTACTGACTT	TTGGGGGACA	CGTTCCAAAC	GCTGTTATCC	TTGCTAATGC
P.heterotricha	CTACTGACTT	TTGGGGGACA	CGTTCCAAAC	GCTGTTATCC	TTGCTAATGC
P.huaijiensis	CTACTGACTT	TTGGGGGACA	CGTTCCAAAT	GCTGTTATCC	TTGCTAATGC
P.lutea	CTACTGACTT	TTGGGGGACA	AGTTCCAAAC	GCTGTTATCC	TTGCTAATGC
P.pteropoda	CTACTGACTT	TTGGGGGACA	CGTTCCAAAC	GCTGTTATCC	TTGCTAATGC
P.sinensis	CTACTGACTT	TTGGGGGACA	CGTTCCAAAC	GCTGTTATCC	TTGCTAATGC
P.swinglei	CTACTGACTT	TTGGGGGACA	CGTTCCAAAC	GCTGTTATAC	TTGCTAATGC
P.tabacum	CTACTGACTT	TTGGGGGACA	CGTTCCCAAC	GCTGTTATCC	TTGCTAATGC
P.villosissima	CTACTGACTT	TTGGGGGACA	CGTTCCAAAC	GCTGTTATCC	TTGCTAATGC

	2160	2170	2180	2190	2200
P.eburnea	TTGTACAAGT	AGGAACTATA	AAAATGATGT	TGTTGGAGTT	TGCTTTATTG
P.fimbrisepala	TTGTACCAGT	AGGAACTATA	AAAATGATGT	TGTTGGAGTT	TGCTTTATTG
P.heterotricha	TTGTACCAGT	AGGAACTATA	AAAATGATGT	TGTTGGAGTT	TGCTTTATTG
P.huaijiensis	TTGTACCAGT	AGGAACTATA	AAAATGATGT	TGTTGGAGTT	TGCTTTATTG
P.lutea	TTGTACCAGT	AGGAACTATA	AAAATGATGT	TGTAGGAGTT	TGCTTTATTG
P.pteropoda	TTGTACCAGT	AGGAACTATA	AAAATGATGT	TGTTGGAGTT	TGCTTTATTG
P.sinensis	TTGTATCAGT	AGGAACTATA	AAAATGATGT	TGTTGGAGTT	TGCTTTATTG
P.swinglei	TTGTACCAGT	AGGAACTATA	CAAATGATGT	TGTTGGAGTT	TGCTTTATTG
P.tabacum	TTGTACCAGT	AGGAACTATA	AAAATGATGT	TGTTGGAGTT	TGCTTTATTG
P.villosissima	TTGTACCAGT	AGGAACTATA	AAAATGATGT	TGTTGGAGTT	TGCTTTATTG

	2210	2220	2230	2240	2250
P.eburnea	GTCAAGATGT	CACAGCTGAG	AAAATAGTGA	TGGACAAGTT	CATCCGCTTG
P.fimbrisepala	GTCAAGATGT	CACAGCTGAG	AAAATAGTGA	TGGACAAGTT	CATCCGCTTG
P.heterotricha	GTCAAGATGT	CACAGCTGAG	AAAATAGTGA	TGGACAAGTT	CATCCGCTTG
P.huaijiensis	GTCAAGATGT	CACAGCTGAG	AAACTAGTGA	TGGACAAGTT	CATCCGCTTG
P.lutea	GTCAAGATGT	CACAGCTGAG	AAAATAGTGA	TGGACAAGTT	CATCCGCTTG
P.pteropoda	GTCAAGATGT	CACAGCTGAG	AAAATAGTGA	TGGACAAGTT	CATCCGCTTG
P.sinensis	GTCAAGATGT	CACAGCTGAG	AAAATAGTGA	TGGACAAGTT	CATCCGCTTG
P.swinglei	GTCAAGATGT	CACAGCTGAG	AAAATAGTGA	TGGACAAGTT	CATCCGCTTG
P.tabacum	GTCAAGATGT	CACAGCTGAG	AAAATAGTGA	TGGACAAGTT	CATCCGCTTG
P.villosissima	GTCAAGATGT	CACAGCTGAG	AAAATAGTGA	TGGACAAGTT	CATCCGCTTG

	2260	2270	2280	2290	2300
P.eburnea	CAAGGGGACT	ACAAGATTAT	TATGCAAAGC	CTAAGTCCAC	TGATTCCGCC
P.fimbrisepala	CAAGGGGACT	ACAAGATTAT	TATGCAAAGC	CTAAGTCCAC	TGATTCCGCC
P.heterotricha	CAAGGGGACT	ACAAGATTAT	TATGCAAAGC	CTAAGTCCTC	TGATTCCTCC
P.huaijiensis	CAAGGGGACT	ACAAGATTAT	TATGCAAAGC	CTAAGTCCAC	TGATTCCGCC
P.lutea	CAAGGGGACT	ACAAGATTAT	TATGCAAAGC	CTAAGTCCAC	TGATTCCGCC
P.pteropoda	CAAGGGGACT	ACAAGATTAT	TATGCAAAGC	CTAAGTCCAC	TGATTCCTCC
P.sinensis	CAAGGGGACT	ACAAGATTAT	TATGCAAAGC	CTAAGTCCAC	TGATTCCGCC
P.swinglei	CAAGGGGACT	ACAAGATTAT	TATGCAAAGC	CTAAGTCCAC	TGATTCCGCC
P.tabacum	CAAGGGGACT	ACAAGATTAT	TATGCAAAGC	CTAAGTCCAC	TGATTCCGCC
P.villosissima	CAAGGGGACT	ACAAGATTAT	AATGCAAAGC	CTAAGTCCAC	TGATTCCGCC

	2310	2320	2330	2340	2350
P.eburnea	TATTTTTGCT	TCAGACGAGA	ATGCCTGTTG	TTCTGAATGG	AATGCAGCCA
P.fimbrisepala	TATTTTTGCT	TCAGACGAGA	ATGCCTGTTG	TTCTGAATGG	AATGCAGCCA
P.heterotricha	TATTTTTGCT	TCAGACGAGA	ATGCCTGTTG	TTCTGAATGG	AATGCGGCCA
P.huaijiensis	TATTTTTGCT	TCAGACGAGA	ATGCTTGTTG	TTCTGAATGG	AATGCAGCCA
P.lutea	TATTTTTGCT	TCAGACGAGA	ATGCCTGTTG	TTCTGAATGG	AATGCAGCCA
P.pteropoda	TATTTTTGCT	TCAGACGAGA	ATGCCTGTTG	TTCTGAATGG	AATGCAGCCA
P.sinensis	TATTTTTGCT	TCAGACGAGA	ATGCCTGTTG	TTCTGAATGG	AATGCAGCCA
P.swinglei	TATTTTTGCT	TCAGACGAGA	ATGCCTGTTG	TTCTGAATGG	AATGCAGCCA
P.tabacum	TATTTTTGCT	TCAGACGAGA	ATGCCTGTTG	TTCTGAATGG	AATGCGGCCA
P.villosissima	TATTTTTGCT	TCAGACGAGA	ATGCCTGTTG	TTCTGAATGG	AATGCAGCCA

	2360	2370	2380	2390	2400
P.eburnea	TGGAAAAGCT	GACTGGCTGG	ATGAAACACG	AGATTATTGG	AAAGATGTTA
P.fimbrisepala	TGGAAAAGCT	GACTGGCTGG	ATGAAACACG	AGATTATTGG	AAAGATGTTA
P.heterotricha	TGGAAAAGCT	GACTGGCTGG	ACGAAACACG	AGATTAATGG	AAAGATGTTA
P.huaijiensis	TGGAAAAGCT	GACTGGCTGG	ATGAAACACG	AGATTATTGG	AAAGATGTTA
P.lutea	TGGAAAAGTT	GACTGGCTGG	ATGAAACATG	AGATTATTGG	AAAGATGTTA
P.pteropoda	TGGAAAAGCT	GACTGGCTGG	ACGAAACACG	AGATTATTGG	AAAGATGTTA
P.sinensis	TGGAAAAGCT	GACTGGCTGG	ATGAAACACG	AGGTTATTGG	AAAGATGTTA
P.swinglei	TGGAAAAGCT	GACTGGCTGG	ATGAAACACG	AGATTATTGG	AAAGATGTTA
P.tabacum	TGGAAAAGCT	GACTGGCTGG	ATGAAACACG	AGATTATTGG	AAAGATGTTA
P.villosissima	TGGAAAAGCT	GACTGGCTGG	ATGAAACACG	AGATTATCGG	AAAGATGTTA

	2410	2420	2430	2440	2450
P.eburnea	CCTGGTGAAA	TTTTTGGAAG	CTTCTGTCGG	CTGAAAGGTC	AGGACGAGCT
P.fimbrisepala	CCTGGTGAAA	TTTTTGGAAG	CTTCTGTCGG	CTGAAAGGTC	AAGACGAGCT
P.heterotricha	CCTGGTGAAA	TTTTTGGAAG	CTTCTGTCGG	CTGAAAGGTC	AAGACGAGCT
P.huaijiensis	CCTGGTGAAA	TATTTGGAAG	CTTCTGTCGG	CTGAAAGGTC	AAGACGAGCT
P.lutea	CCTGGTGAAA	TTTTTGGAAG	CTTCTGTCGG	CTGAAAGGTC	AAGACGAGCT
P.pteropoda	CCTGGTGAAA	TTTTTGGAAG	CTTCTGTCGG	CTGAGAGGTC	AAGACGAGCT
P.sinensis	CCTGGTGAAA	TTTTTGGAAG	CTTCTGTCGG	CTGAAAGGTC	AAGACGAGCT
P.swinglei	CCTGGTGAAA	TTTTTGGAAG	CTTCTGTAGG	CTGAAAGGTC	AGGACGAGCT
P.tabacum	CCTGGTGAAA	TTTTTGGAAG	CTTCTGTCGG	CTGAAAGGTC	AGGATGAGCT
P.villosissima	CCTGGTGAAA	TTTTTGGAAG	CTTCTGTCGG	CTGAAAGGTC	AAGACGAGCT

	2460	2470	2480	2490	2500
P.eburnea	GACTAAATTC	ATGATTCTTT	TTTACCGAGC	AATTAGCGGC	CAGGACACTC
P.fimbrisepala	GACTAAATTC	ATGATTCTTT	TGTACCGAGC	AATTAGTGGC	CAGGACACTC
P.heterotricha	GACTAAATTC	ATGATTCTTT	TGTACCGAGC	AATTAGTGGC	CAGGACACTC
P.huaijiensis	GACTAAATTC	ATGATTCTTT	TGTACCGAGC	AATTAGTGGC	CAGGACACTC
P.lutea	GACTAAATTC	ATGATTCTTT	TGTACCGAGC	AATTAGTGGC	CAGGACACTC
P.pteropoda	GACTAAATTC	ATGATTCTTT	TGTACCGAGC	AATTAGTGGC	CAGGACACTC
P.sinensis	GACTAAATTC	ATGATTCTTT	TGTACCGAGC	AATTAGTGGC	CAGGACACTC
P.swinglei	GACTAAATTC	ATGATTCTTT	TGTACCGAGC	AATTAGTGGC	CAGGACACTC
P.tabacum	GACTAAATTC	ATGATTCTTT	TGTACCGAGC	AATTAGTGGC	CAGGACAATC
P.villosissima	GACTAAATTC	ATGATTCTTT	TGTACCGAGC	AATTAGTGGC	CAGGACACTC

	2510	2520	2530	2540	2550
P.eburnea	AAACGCTCCC	ATTTGGTTTT	TTTGACCGGA	GTGGTGAGTT	TGTCGATGTG
P.fimbrisepala	AAACGCTCCC	ATTTGGTTTT	TTTGACCGGA	GAGGTGAGTT	TGTCGATGTG
P.heterotricha	AAACGCTCCC	ATTTGGTTTT	TTTGACCGGA	GAGGTGAGTT	TGTCGATGTG
P.huaijiensis	AAACGCTACC	ATTTGGTTTT	TTTGACCGGA	GAGGTGAGTT	TGTCGATGTG
P.lutea	AAACGCTCCC	ATTTGGTTTT	TTTGACCGGA	GAGGTGAGTT	TGTCGATGTG
P.pteropoda	AAACGCTCCC	ATTTGGTTTT	TTTGACCGGA	GAGGTGAGTT	TGTCGATGTG
P.sinensis	AAACGCTCCC	ATTTGGTTTT	TTTGACCGGA	GAGGTGAGTT	TGTCGACGTG
P.swinglei	AAACACTCCC	ATTTGGTTTT	TTTGACCGGA	GAGGTGAGTT	TGTCGATGTG
P.tabacum	AAACGCTCCC	ATTTGGTTTT	TTTGACCGGA	GAGGTGAGTT	TGTCGATGTG
P.villosissima	AAACGCTTCC	ATTTGGTTTT	TTTGACCGGA	GAGGTGAGTT	TGTCGATGTG

	2560	2570	2580	2590	2600
P.eburnea	CTTTTGACAG	CAAATAAGAA	AACAGAGGAG	GGTGGGAATG	TGAACGGGTG
P.fimbrisepala	CTTTTGACAG	CAAATAAGAA	AACAGAGGAG	GGTGGGAATG	TGAACGGGTG
P.heterotricha	CTTTTGACAG	CAAATAAGAA	AACAGAGGAG	GGTGGGAATG	TGAACGGGTG
P.huaijiensis	CTTTTGACAG	CAAATAAGAA	AACAGAGGAG	GGTGGGAATG	TGAACGGGTG
P.lutea	CTTTTGACAG	CAAATAAGAA	AACAGAGGAG	GGTGGGAATG	TGAACGGGTG
P.pteropoda	CTTTTGACAG	CAAATAAGAA	AACAGAGGAG	GGTGGGAATG	TGAACGGGTG
P.sinensis	CTTTTGACAG	CGAATAAGAA	AACAGAGGAG	GGTGGGAATG	TGAACGGGTG
P.swinglei	CTTTTGACAG	CAAATAAGAA	AACAGAGGAG	GGTGGGAATG	TGAACGGGTG
P.tabacum	CTTTTGACAG	CAAATAAGAA	AACAGAGGAG	GGTGGGAATG	TGAACGGGTG
P.villosissima	CTTTTGACAG	CAAATAAGAA	AACAGAGGAG	GGTGGGAATG	TGAACGGGTG

	2610	2620	2630	2640	2650
P.eburnea	TTTCTGCTTC	TTGCAGACTG	TACCGAAACA	GCAACAGTTC	ATGTTAGATA
P.fimbrisepala	TTTCTGCTTC	TTGCAGACTG	TACCAAAACA	GCAACAGTTC	ATGTTAGATA
P.heterotricha	TTTCTGCTTC	TTGCAGACTG	TACCGAAACA	GCAGCAGTTC	GTGTTAAATG
P.huaijiensis	TTTCTGCTTC	TTGCAGACTG	TCCCGAAACA	GCATCAGTTC	ATGTTAGATA
P.lutea	TTTCTGCTTC	TTGCAGACTG	TACCGAAACA	GCAACAGTTC	ATGTTAGATA
P.pteropoda	TTTCTGCTTC	TTGCAGACTG	TACCGAAACA	GCAGCAGTTC	GTGTTAAATA
P.sinensis	TTTCTGCTTC	TTGCAGACTG	TACCGAAACA	GCAGCAGTTC	ATGTCAGATA
P.swinglei	TTTCTGCTTC	TTGCAGACTG	TACCGAAACA	GCAGCAATTC	ATGTTAGATA
P.tabacum	TTTCTGCTTC	TTGCAGACTG	TACCGAAACA	GCAACAGTTC	ATGTTAGATA
P.villosissima	CTTCTGCTTC	TTGCAGACTG	TACCGAAACA	GCAACAGTTC	ATGTTAGATA

	2660	2670	2680	2690	2700
P.eburnea	AGTCGGAAGG	AAAGGAGCAG	CTTTCAAAAC	TTAAAGAGTT	GGCTTACGTT
P.fimbrisepala	AGTCGGAAGG	AAAGGAGCAG	CTTTCAAAAC	TTAAAGAGTT	GGCTTACATA
P.heterotricha	AGTCGGAAGG	AAAGGATCAG	CTTTCAAAAC	TTAAAGAGTT	GGCTTACATT
P.huaijiensis	AGTCGGAAGG	AAAGGAGCAG	CTTTCGAAAC	TTAAAGAGTT	GGCTTATATT
P.lutea	AGTCAGAAGG	AAAGGAGCAG	CTTTCAAAAC	TTAAAGAGTT	GGCTTACATT
P.pteropoda	AGTCGGAAGG	AAACGATCAG	CTTTCAAAAC	TTAAAGAGTT	GGCTTACATT
P.sinensis	AGTCGGAAGG	AAAGGAGCAG	CTTTCAAAAC	TTAAAGAGTT	GGCTTACATT
P.swinglei	AGTCGGAAGG	AAAGGAGCAG	CTTTCAAAAC	TTAAAGAGTT	GGCTTACATT
P.tabacum	AGTCGGAAGG	AAAGGAGCAG	CTTTCAAAAC	TTAAAGAGTT	GGCTTACATT
P.villosissima	AGTCGGAAGG	AAAGGAGCAG	CTTTCAAAAC	TTAAAGAGTT	GGCTTACATT

```
                        2710       2720       2730       2740       2750
                 ....|....| ....|....| ....|....| ....|....| ....|....|
P.eburnea        AGGCAGGAGA TGAAGAATCC TCTAAATGGA ATCCGCTTCA CACATGGACT
P.fimbrisepala   AGGCAGGAGA TGAAGAATCC TCTAAATGGA ATCCGCTTCA CACATGGACT
P.heterotricha   AGGCAGGAGA TGAAGAATCC TCTAAATGGA ATCCGCTTCA CACATGGACT
P.huaijiensis    AGGCAGGAGA TGAAGAATCC TCTAAATGGA ATCCGCTTCA CACATGGACT
P.lutea          AGGCAGGAGA TGAAGAATCC TCTAAATGGA ATCCGCTTCA CACATGGACT
P.pteropoda      AGGCAGGAGA TGAAGAATCC TCTAAATGGA ATCCGCTTCA CACATGGACT
P.sinensis       AGGCAGGAGA TGAAGAATCC TCTAAATGGA ATCCGCTTCA CACATGGACT
P.swinglei       AGGCAGGAGA TGAAGAATCC TCTAAATGGA ATCCGCTTCA CACATGGACT
P.tabacum        AGGCAGGAGA TGAAGAATCC TCTAAATGGA ATCCGCTTCA CACATGGACT
P.villosissima   AGGCAGGAGA TGAAGAATCC TCTAAATGGA ATCCGCTTCA CACATGGACT

                        2760       2770       2780       2790       2800
                 ....|....| ....|....| ....|....| ....|....| ....|....|
P.eburnea        CCTGGGAAAT TCAGCTCTAA CAGAAGACCA GAAACGGTTT ATTGAGACTA
P.fimbrisepala   CCTGGGAAAT TCGGCTCTAA CAGAAGACCA GAAACGGTTT ATTGAGACTA
P.heterotricha   CCTGGGAAAT TCGGCTCTAA CAGAAGACCA GAAACGGTTT ATTGAGACTA
P.huaijiensis    CCTGGGAAAT TCGGCTCTAA CAGAAGACCA GAAACGGTTT ATTGAGACTA
P.lutea          CCTGGGAAAT TCAGCTCTAA CAGAAGACCA GAAACGGTTT ATTGAGACTA
P.pteropoda      CCTGGGAAAT TCGGCTCTAA CAGAAGACCA GAAACGGTTT ATTGAGACCA
P.sinensis       CCTGGGAAAT TCGGCTCTAA CAGAAGACCA GAAACGGTTT ATTGAGACTA
P.swinglei       CATGGGAAAT TCGGCTCTAA CAGAAGACCA GAAACGGTTT ATTGAGACTA
P.tabacum        CCTGGGAAAT TCGGCTCTAA CAGAAGACCA GAAACGGTTT ATTGAGACTA
P.villosissima   CCTGGGAAAT TCGGCTCTGA CAGAAGACCA GAAACGGTTT ATTGAGACTA

                        2810       2820       2830       2840       2850
                 ....|....| ....|....| ....|....| ....|....| ....|....|
P.eburnea        GTGAAGCTTG TGAAAGACAA ATTTTATCTA TTTTAGACGA TCCACATTTT
P.fimbrisepala   GCGAAGCTTG TGAAAGACAA ATTTTATCTA TTTTAGACGA TCCACATTTT
P.heterotricha   GTGAAGCTTG TGAAAGACAA ATTTTATCTA TTTTAGACGA TCCACATTTT
P.huaijiensis    GTGAAGCTTG TGAAAGACAA ATTTTATCTA TTTTAGACGA TCCACATTTT
P.lutea          GCGAAGCTTG TGAAAGACAA ATTTTATCTA TTTTAGACGA TCCACATTTT
P.pteropoda      GTGAAGCTTG TGAAAGACAA ATTTTATCTA TTTTAGACGA TCCACATTTT
P.sinensis       GCGAAGCTTG TGAAAGACAA ATTTTATCTA TTTTAGACGA TCCACATTTT
P.swinglei       GTGAAGCTTG CGAAAGACAA ATTTTATCTA TTTTAGACGA TACACATTTT
P.tabacum        GCGAAGCTTG TGAAAGACAA ATTTTATCTA TTTTAGACGA TCCACATTTT
P.villosissima   GCGAAGCTTG CGAAAGACAA ATTTTATCTA TTTTAGATGA TCCACATTTT

                        2860       2870       2880       2890       2900
                 ....|....| ....|....| ....|....| ....|....| ....|....|
P.eburnea        GGAAGTTCAC AAGAAGGTGA ACTGGAGCTT AAGATGGAAG AGTTTTTCTT
P.fimbrisepala   GGAAGTTCAC AAGAAGGTGA ACTGGAGCTT AAGATGGAAG AGTTTTTCTT
P.heterotricha   GGAAGTTCAC AAGAAGGTGA ACTGGAGCTT AAGATGGAAG AGTTTTTCTT
P.huaijiensis    GGAAGTTCAC AAGAAGGTGA ACTGGAGCTT AAGATGGAAG AGTTTTTCTT
P.lutea          GGAAGTTCAC AAGAAGGTGA ACTGGAGCTT AAGATGGAAG AGTTTTTCTT
P.pteropoda      GGAAGTTCAC AAGAAGGTGA ACTGGAGCTT AAGATGGAAG AGTTTTTCTT
P.sinensis       GGAAGTTCAC AAGAAGGTGA ACTGGAGCTT AAGATGGAAG AGTTTTTCTT
P.swinglei       GGAAGTTCAC AAGAAGGTGA ACCGGAGCTT AAGATGGAAG AGTTTTTCTT
P.tabacum        GGAAGTTCAC AAGAAGGTGA ACTGGAGCTT AAGATGGAAG AGTTTTTCTT
P.villosissima   GGAAGTTCAC AAGAAGGTGA ACTGGAGCTT AAGATGGAAG AGTTTTTCTT

                        2910       2920       2930       2940       2950
                 ....|....| ....|....| ....|....| ....|....| ....|....|
P.eburnea        GGGGAATGTT ATCAACGCTA TTGTTTGTCA GGCTATGATA TTGTTGAATG
P.fimbrisepala   GGGGAATGTT ATCAACGCTA TTGTTTGTCA GGCTATGATA TTGTTGAATG
P.heterotricha   GGGGGATGTT ATCAACGCTA TTGTTTGTCA GGCTATGATA CTGTTGAATG
P.huaijiensis    GGGGAATGTT ATCAACGCTA TTGTTTGTCA GGCTATGATA TTGTTGAATG
P.lutea          GGGGAATGTT ATCAACGCTA TTGTTTGTCA GGCTATGATA TTGTTGAATG
P.pteropoda      GGGGGATGTT ATCAACGCTA TTGTTTGTCA GGCTATGATA CTGTTGAACG
P.sinensis       GGGGAATGTT ATCAACGCTA TTGTTTGTCA GGCTATGATA TTGTTGAATG
P.swinglei       GGGGAATGTT ATCAACGCTA TTGTTTGTCA GGCTATGATA CTGTTGAATG
P.tabacum        GGGGAATGTT ATCAACGCTA TTGTTTGTCA GGCTATGATA TTGTTGAATG
P.villosissima   GGGGAATGTT ATCAACGCTA TTGTTTGTCA GGCTATGATA TTGTTGAATG

                        2960       2970       2980       2990       3000
                 ....|....| ....|....| ....|....| ....|....| ....|....|
P.eburnea        AAAAGAATTT GAAACTAGTA CTGGATGTCT CTGAGCGAAT TAGAACTCTT
P.fimbrisepala   AAAAGAATTT GAAACTAGTA CTGGATGTCT CTGAGCGAAT TAGAACTCTT
P.heterotricha   AAAAGAATTT GAAACTAGTA CTGGATGTCT CTGAGCGAAT TAGAACTCTT
P.huaijiensis    AAAAGAATTT GAAACTAGTA CTGGATGTCT CTGAGCGAAT TAGAACTATT
P.lutea          AAAAGAATTT GAAACTAGTA CTGGATGTCT CTGAGCGAAT TAGAACTCTT
P.pteropoda      AAAAGAATTT GAAACTAGTA CTGGATGTCT CTGAGCGAAT TAGAACTCTT
P.sinensis       AAAAGAATAT GAAACTAGTA CTGGATGTCT CTGAGCGAAT TAGAACTCTT
P.swinglei       AAAAGAATTT GAAACTAGTA CTGGATGTCT CTGAGCGAAT TAGAACTCTT
P.tabacum        AAAAGAATTT GAAACTAGTA CTGGATGTCT CTGAGCGAAT TAGAACTCTT
P.villosissima   AAAAGAATTT GAAACTAGTA CTGGATGTCT CTGAGCGAAT TAGAACTCTT
```

```
                    3010       3020       3030       3040       3050
P.eburnea       TGTTTATATG GGGATCAAAT AAAGCTTCAG TTAGCCTTGT CTGATTTCTT
P.fimbrisepala  TGTTTATATG GGGATCAAAT AAAGCTTCAG TTAGCCTTGT CTGATTTCTT
P.heterotricha  TGTTTATATG GAGATCAAAT AAAGCTTCAG TTAGCCTTGT CTGATTTCTT
P.huaijiensis   TGTTTATATG GGGATCAAAT AAAGCTTCAG TTAGCCTTGT CTGATTTCTT
P.lutea         TGTTTATATG GGGATCAAAT AAAGCTTCAG TTAGCCTTGT CTGATTTCTT
P.pteropoda     TGTTTATATG GAGATCAAAT AAAGCTTCAG TTAGCCTTGT CTGATTTCTT
P.sinensis      TGTTTATATG GGGATCAAAT AAAGCTTCAG TTAGCCTTGT CTGATTTCTT
P.swinglei      TGTTTATATG GAGATCAGAT AAAGCTTCAG TTAGCCTTGT CTGATTTCTT
P.tabacum       TGTTTATATG GGGATCAAAT AAAGCTTCAG TTAGCCTTGT CTGATTTCTT
P.villosissima  TGTTTATATG GGGATCAAAT AAAGCTTCAG TTAGCCTTGT CTGATTTCTT

                    3060       3070       3080       3090       3100
P.eburnea       GCTCAGTGTG GTGGACCATA CACCTTGTCC TGATGGTTGG GTGGAAATTA
P.fimbrisepala  GCTCAGTGTG GTGGACCATA CACCTTGTCC TGATGGTTGG GTGGAAATTA
P.heterotricha  GCTCAGTGTG GTGGACCATA CACATTGTCC CGATGGTTGG GTGGAAATAA
P.huaijiensis   GCTCAGTGTG GTGGACCATA CACCTTGTCC TGATGGTTGG GTGGAAATTA
P.lutea         GCTCAGTGTG GTGGACCATA CACCTTGTCC TGATGGTTGG GTGGAAATTA
P.pteropoda     GCTCAGTGTG GTGGACCATA CACATTGTCC CGATGGTTGG GTGGAAATAA
P.sinensis      GCTCAGTGTG GTGGACCATA CACCTTGTCC TGATGGTTGG GTGGAAATTA
P.swinglei      GCTCAGTGTG GTGGACCATA CACCTTGTCC TGATGGTTGG GTGGAAATTA
P.tabacum       GCTCAGTGTG GTGGACCATA CACCTTGTCC TGATGGTTGG GTGGAAATTA
P.villosissima  GCTCAGTGTG GTGGACCATA CACATTGTCC TGATGGTTGG GTGGAAATTA

                    3110       3120       3130       3140       3150
P.eburnea       TGGTATCCCC TGGTTTGAAA CTGATGCAAG ACGGGAATGA GTCTGTTCAC
P.fimbrisepala  TGGTATCCCC TGGTTTGAAA CTGATGCAAG ACGGGAATGA GTCTGTTCAC
P.heterotricha  TGGTATCCCC TGGTTTGAAA CTGATGCAAG ACGGGAATGA GTCTGTTCAC
P.huaijiensis   TGGTATCCCC TGGTTTGAAA CTGATGCAAG ACGGGAATGA GTCTGTTCAA
P.lutea         TGGTATCCCC TGGTTTGAAA CTGATGCAAG ACGGGAATGA GTCTGTTCAC
P.pteropoda     TGGTATCCCC TGGTTTGAAA CTGATGCAAG ACGGGAATGA GTCTGTTCAC
P.sinensis      TGGTATCCCC TGGTTTGAAA CTGATGCAAG ACGGGCATGA GTCTGTTCAC
P.swinglei      TGGTATCCCC TGGTTTGAAA CTGATGCAAG ACGGGAATGA GTCTGTTCAC
P.tabacum       TGGTATCCCC TGGTTTGAAA CTGATGCAAG ACGGGAATGA GTCTGTTCAC
P.villosissima  TGGTATCCCC TGGTTTGAAA CTGATGCAAG ACGGGAATGA GTCTGTTCAC

                    3160       3170       3180       3190       3200
P.eburnea       CTACAATTCA GAATGGCACA CCCTGGCCAG GGACTTCCCT CTTCTCTTAT
P.fimbrisepala  CTACAATTCA GAATGGCACA CCCTGGCCAG GGCCTTCCCT CTTCTCTTAT
P.heterotricha  CTACAATTCA GAATGGCACA CCCTGGCCAG GGCCTTCCCT CTTCTCTTAT
P.huaijiensis   CTACAAGTCA GAATGGCACA CCCTGGCCAG GGCCTTCCCT CTTCTCTTAT
P.lutea         CTACAATTCA GAATGGCACA CCCTGGCCAG GGCCTTCCCT CTTCTCTTAT
P.pteropoda     CTACAATTCA GAATGGCACA CCCTGGCCAG GGCCTTCCCT CTTCTCTTAT
P.sinensis      CTACAATTCA GAATGGCACA CCCTGGCCAG GGCCTTCCCT CTTCTCTTAT
P.swinglei      CTACAATTCA GAATGGCACA CCCTGGCCAG GGCCTTCCCT CTTCTCTTGT
P.tabacum       CTACAATTCA GAATGGCACA CCCTGGCCAG GGCCTTCCCT CTTCTCTTAT
P.villosissima  CTACAATTCA GAATGGCACA CCCTGGCCAG GGCCTTCCCT CTTCTCTTAT

                    3210       3220       3230       3240       3250
P.eburnea       TGAAGATATG TATGCAGTGA AAAGTAACTG GTTAACTCAG CCAGGAATTG
P.fimbrisepala  TGAAGATATG TTTGCAGTGA AAAGTAACTG GTTAAGTCAG CGAGGAGCTA
P.heterotricha  TGAAGATATG TTTGCGGTGA AAAGTAACTG GTTGACTCAG CGAGGAATTG
P.huaijiensis   TGAAGATATG TTTGCAGTGA AAAGTAACTG GTCAACTCAG CGAGGAATTG
P.lutea         TGAAGATATG TATGCAGTGA AAAGTAACTG GTTAACTCAG CCAGGAATTG
P.pteropoda     TGAAGATATG TTTGCGGTGA AAAGTAACTG GTTGACTCAG CGAGGAATTG
P.sinensis      TGAAGATATG TTTGCAGTGA AAAGTAACTG GTTAAATCAG CGAGGAATTG
P.swinglei      TGAAGATATG TTTGCAGTGA AAAGTAACTG GTTAACTCAG CGAGGAATTG
P.tabacum       TGAAGATATG TATGCAGTGA AAAGTAACTG GTTAACTCAG CGAGGAATTG
P.villosissima  TGAAGATATG TTTGCAGTGA AAAGTAACTG GTTAAGTCAG CGAGGAACTA

                    3260       3270       3280       3290       3300
P.eburnea       CTCTCAACAT TGCTCAGAAA CTTCTCAAAA CGATGAATGG TAATGTCCGG
P.fimbrisepala  CTCTCAACAT TGCTCAGAAA CTTCTCAAAA CGATGAATGG TAATGTCCGG
P.heterotricha  CTCTCAACAT TGCTCAGAAA CTTCTCAAAA CGATGAATGG TAATGTCCGG
P.huaijiensis   CTCTCAACAT TGCTCAGAAA CTTCTCAAAA CGATGAATGG TAATGTCCGG
P.lutea         CTCTCAACAT TGCTCAGAAA CTTCTCAAAA CGATGAATGG TAATGTCCGG
P.pteropoda     CTCTCAACAT TGCTCAGAAA CTTCTCAAAA CGATGAATGG TAATGTCCGG
P.sinensis      CTCTCAACAT GGCTCAGAAA CTTCTCAAAA CGATGAATGG TAATGTCCGG
P.swinglei      CTCTCAACAT TGCTCAGAAA CTTCTCAAAA CGATGAACGG TAATGTCCGG
P.tabacum       CTCTCAACAT TGCTCAGAAA CTTCTTAAAA CGATGAATGG TAATGTCCGG
P.villosissima  CTCTCAACAT TGCTCAGAAA CTTCTCAAAA CGATGAATGG TAATGTCCGG
```

	3310	3320	3330	3340	3350
P.eburnea	TATATCAGAG	ATCAGAGTAG	ATGTTACTTC	TTAGTTGATC	TTGAACTCAT
P.fimbrisepala	TATATCAGAG	ATCAGAGTAG	ATGTTACTTC	TTAGTTGATC	TTGAACTCAT
P.heterotricha	TATATCAGAG	ATCAGAGTAG	ATGTTACTTC	TTAGTTGATC	TTGATCTCAT
P.huaijiensis	TATATCAGAG	ATCAGAGTAG	ATGTTACTTC	TTAGTTGATC	TTGAACTCGT
P.lutea	TATATCAGAG	ATCAGAGTAG	ATGTTACTTC	TTAGTTGATC	TTGAACTCAT
P.pteropoda	TATATCAGAG	ATCAGAGTAG	ATGTTACTTC	TTAGTTGATC	TTGATCTCAT
P.sinensis	TATATCAGAG	ATCAGAGTAG	ATGTTACTTC	TTAGTTGATC	TTGAACTCAT
P.swinglei	TATATCAGAG	ATCAGAGTAG	ATGTTACTTC	CTAGTTGATC	TTGAGCTTAT
P.tabacum	TATATCAGAG	ATCAGAGTAG	ATGTTACTTC	TTAGTTGATC	TTGAACTCAT
P.villosissima	TATATCAGAG	ATCAGAGTAG	ATGTTACTTC	TTAGTTGATC	TTGAACTCAT

	3360	3370	
P.eburnea	TTCACGGAAA	TCAGATCAAT	AA
P.fimbrisepala	TTCACGGAAA	TCAGATCAAT	AA
P.heterotricha	TTCACGGAAA	TCAGATCAAT	AA
P.huaijiensis	TTCACGGAAA	TCAGATCAAT	AA
P.lutea	TTCACGGAAA	TCAGATCAAT	AA
P.pteropoda	TTCACGGAAA	TCAGATCAAT	AA
P.sinensis	TTCACGGAAA	TCAGATCAAT	AA
P.swinglei	TTCGCGGAAA	TCAGATCAAT	AA
P.tabacum	TTCACGGAAA	TCAGTTCAAT	AA
P.villosissima	TTCACGGAAA	TCAGATCAAT	AA
